# Including tree spatial extension in the evaluation of neighborhood competition effects in Bornean rain forest

**DOI:** 10.1002/ece3.7452

**Published:** 2021-05-06

**Authors:** David M. Newbery, Peter Stoll

**Affiliations:** ^1^ Institute of Plant Sciences University of Bern Bern Switzerland; ^2^ Section of Conservation Biology Department of Environmental Sciences University of Basel Basel Switzerland

**Keywords:** conspecific and heterospecific effects, crown and root processes, negative density dependence, neighborhood models, symmetric and asymmetric competition, tree growth and survival

## Abstract

Classical tree neighborhood models use size variables acting at point distances. In a new approach here, trees were spatially extended as a function of their crown sizes, represented impressionistically as points within crown areas. Extension was accompanied by plasticity in the form of crown removal or relocation under the overlap of taller trees. Root systems were supposedly extended in a similar manner. For the 38 most abundant species in the focal size class (10–<100 cm stem girth) in two 4‐ha plots at Danum (Sabah), for periods P_1_ (1986–1996) and P_2_ (1996–2007), stem growth rate and tree survival were individually regressed against stem size, and neighborhood conspecific (CON) and heterospecific (HET) basal areas within incremented steps in radius. Model parameters were critically assessed, and statistical robustness in the modeling was set by randomization testing. Classical and extended models differed importantly in their outcomes. Crown extension weakened the relationship of CON effect on growth versus plot species’ abundance, showing that models without plasticity overestimated negative density dependence. A significant negative trend of difference in CON effects on growth (P_2_−P_1_) versus CON or HET effect on survival in P_1_ was strongest with crown extension. Model outcomes did not then support an explanation of CON and HET effects being due to (asymmetric) competition for light alone. An alternative hypothesis is that changes in CON effects on small trees, largely incurred by a drought phase (relaxing light limitation) in P_2_, and following the more shaded (suppressing) conditions in P_1_, were likely due to species‐specific (symmetric) root competition and mycorrhizal processes. The very high variation in neighborhood composition and abundances led to a strong “neighborhood stochasticity” and hence to largely idiosyncratic species’ responses. A need to much better understand the roles of rooting structure and processes at the individual tree level was highlighted.

## Graphical abstract

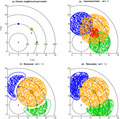

## 1 INTRODUCTION

One of most important advances in estimating and understanding dynamics of trees within forest communities was made when statistical analysis and population modeling moved away from the application of species or guild parameter averages and replaced them with spatially explicit estimates (DeAngelis & Mooij, 2005; DeAngelis & Yurek, 2017). Each and every individual was considered, its growth, survival, and where possible its reproductive output, with reference to its neighbors. Neighbors are other trees close enough to the focal one to affect its resource acquisition and uptake (Pacala et al., 1996; Pacala & Deutschman, 1995; Uriarte et al., 2004). Given that competition is a driving process of change in tree species abundance locally, differences in biomass, architecture, and ecophysiological traits between focal trees and neighbors will in part be determining forest dynamics (Chen et al., 2018, 2019). Mean parameters often obscure differences between species, especially when variables are non‐normally distributed and relationships are nonlinear.

In addition to what can be measured and modeled deterministically, individuals from recruitment onwards are subject to demographic stochasticity affecting their survival (Engen et al., 1998; Lande et al., 2003). Environmental stochasticity in the form of climate variability (particularly rainfall and temperature) is thought to also play an essential role in forest dynamics (Halley, 2007; Vasseur & Yodzis, 2004). This form of stochasticity affects not only individual tree growth and survival directly, but also does so indirectly through its effects on neighbors and hence *their* competitive influence on the individual. Focal trees are simultaneously acting as neighbors to other ones nearby and reciprocal interactions operate. Due to these highly complicated and varying local tree environments, a form of what may be termed “neighborhood stochasticity” is realized. Any understanding of species‐specific effects in neighborhood modeling has, therefore, to cater for this inherently high system variability.

Within this conceptual framework of ongoing temporal and spatial variability, the role of neighbors on the growth and survival of small trees in tropical rain forests is analyzed more closely in the present paper. The data come from a long‐term dynamics study at Danum in Sabah, NE Borneo. One motivation was to resolve better what constitutes conspecific (vs. heterospecific) competition between trees; the other was to get closer to unraveling the role of below‐ground processes, in the search for a mechanism. The new work builds on Stoll and Newbery (2005) and Newbery and Stoll (2013). To introduce the approach, it is first necessary to give the background of the previous Danum studies and modeling results to date, and then second to argue for the proposed extension, hypotheses and tests. As with all sites, data and model are context‐dependent and contingent on site history. The principles behind the analysis, however, should hopefully be relevant to other rain forest sites when making similar considerations.

### 1.1 Current tree neighborhood model

In the 10‐year period of relatively little environmental climatic disturbance (P_1_:1986–1996), large trees of several species among the overstory dipterocarps at Danum showed strong conspecific negative effects on the growth rates of juvenile trees in their immediate neighborhood (Stoll & Newbery, 2005). In the subsequent 11‐year period (P_2_:1996–2007) which included an early moderately strong El Nino Southern Oscillation (ENSO) event (April 1998), conspecific effects relaxed (Newbery & Stoll, 2013). If the effects of the first period were a result of intraspecific competition, perhaps principally for light, then the dry conditions caused by the event in the second, which temporarily thinned the overstory foliage and markedly increased small twig abscission (Walsh & Newbery, 1999), would have allowed more illumination to the understory and hence ameliorated the earlier P_1_ conspecific effects. However, that conspecific effect could be for light presents a problem for two reasons. One is that heterospecific negative effects appeared to be much weaker or nonoperational in P_1_ (Stoll & Newbery, 2005), and the other is the difficulty of explaining a competitive effect for light (i.e., a mechanism of shading) that is species‐specific. Large trees will presumably shade smaller ones regardless of their taxonomic identity, although responses to shading by affected trees might differ between species due to their physiologies. The two periods of measurement may also have differed in other respects besides intensity of drought stress and light changes, and these remain unrecorded or unknown. In terms of succession, the forest at Danum also advanced between P_1_ and P_2_, although still remaining within the late stage of its long‐term recovery from a historically documented period of extensive dryness in Borneo in the late 19th century, with tree basal area continuing to rise and overall tree density decreasing (Newbery et al., 1992, 1999; Newbery & Stoll, 2013).

The hypothesis advanced by Stoll and Newbery (2005) was that interactions below ground may primarily have been causing the conspecific effects for dipterocarps, in the form of competition for nutrients combined with, or enhanced by, host specialist ectomycorrhizal (ECM) linkages between adult and juvenile trees within species. This would be particularly relevant for species of the Dipterocarpaceae, the dominant tree family in these forests, and which accordingly have the highest neighborhood basal areas associated with the strongest conspecific effects. In stem size, focal juveniles were 10–100 cm girth at breast height, gbh (1.3 m above ground, equivalently ~3–30 cm diameter, dbh) and were therefore well‐established small‐to‐medium trees in the understory and lower canopy (Newbery et al., 1992, 1996). Compared to these small trees with their lower‐positioned shaded crowns, the higher demands of the large well‐lit and fast‐growing adults above them may have been making relatively high demands on soil nutrients and thereby drawing these resources away from the juveniles. As a result, the slowed juvenile stem growth may have been due to root competition, enhanced possibly by ECMs. Increased light levels in P_2_, even moderately and temporarily in 1998–99 (Newbery & Lingenfelder, 2004, 2009; Walsh & Newbery, 1999), likely allowed suppressed conspecific juveniles to attain higher growth rates than those in P_1_ (Newbery et al., 2011). It was postulated that that was in part or wholly caused by smaller trees reversing the nutrient flow back from the larger ones, that is, from juvenile to adult (Stoll & Newbery, 2005).

The extent and nature of any ECM linkages and the changing nutrient flows have not been experimentally demonstrated for this forest, so the nutrient hypothesis is tentative. It is difficult to conceive, though, of another mechanism that could explain the results, at least in physical and physiological terms. Against the hypothesis though is broader evidence that the degree of host specialism for ECM fungi in the dipterocarps may be weak because most dipterocarps appear to have many fungal species in common (Alexander & Lee, 2005; Brearley, 2012; Peay et al., 2015). While generalist ECMs were recorded mainly for seedlings and some adults, small‐to‐medium‐sized trees might have been more strongly linked to adults via specialist ECMs, in a period of tree development when dependence on ectomycorrhizas for nutrient supply would be more important than in the earlier ontological stages. This differentiation would be particularly relevant for overstory dipterocarps. While it is quite possible that carbon moves through a general mycorrhizal network linking adults to seedlings when the latter are really very small and in deep shade (Selosse et al., 2006; Simard & Durall, 2004; Simard et al., 2002), it does not mean necessarily that generalist ECMs would function in this same way after the sapling stage, as the small trees became gradually more illuminated. It is also feasible that the element most important for tree interactions changed over time from carbon to phosphorus as the nature of the ECM symbiosis switched from being generalist to specialist.

An alternative hypothesis is that conspecific effects as such were happening “by default” (Newbery & Stoll, 2013). Because, in some species, adults and juveniles tend to be spatially clustered due to the limited distances to which especially dipterocarp seeds are dispersed, conspecifics often made up most of the large‐tree adult neighbor basal area around a focal juvenile. Conversely, some species lacked aggregations possibly because, where more‐scattered juveniles now survive, the parents had recently died. Dipterocarps, and other overstory species, show a wide range of aggregation at different scales (Newbery et al., 1996; Newbery & Ridsdale, 2016; Stoll & Newbery, 2005). Compared with a forest in which trees might theoretically be all distributed at complete randomness, one with aggregations would result in proportionally more trees of the same species (conspecifics) rather than different ones (heterospecifics), occurring at close distances. This fact would tend to an explanation of conspecific effects based on one common mechanism (such as shading), and the effect of the ENSO disturbance in P_2_ was to release understory small trees of all species, to differing degrees depending on each species’ degree of responsiveness to light increases. The role of ECM linkages and nutrient flows would then become secondary, operating as a consequence of light effects (Newbery & Stoll, 2013). Several overstory species in the P_1_‐P_2_ comparison were not dipterocarps, however (presumably they had no ECMs), yet they still showed strong conspecific effects in P_1_, which were relaxed in P_2_ (Newbery & Stoll, 2013). Possibly, these other species with strong CON effects were endomycorrhizal and had similar degrees of specialism like those with ECMs. Strength of conspecific effect was furthermore not convincingly related to degree of spatial clustering within the dipterocarps (Stoll & Newbery, 2005). The two resource‐based hypotheses, “light” versus “nutrients,” were not readily separable, and an extended approach was needed to better distinguish between them.

### 1.2 Extending the neighborhood model

Modeling attempts to date have mostly taken basal areas of neighbors around focal individuals defined by the radial distances between centers of tree stems, normally weighting each neighbor tree's basal area by the inverse of distance (Canham et al., 2004, 2006; Canham & Uriarte, 2006). Whether a tree was inside a circle of a given radius or within a 1‐m annulus, or not, depended solely on the coordinates of its center as a point distribution: Focal and neighbor trees had no spatial extent. Competitive influences and ECM networking might therefore be more realistically represented by the allometric extension of crowns and root systems in the form of a zone of influence, or ZOI (Bella, 1971; Ek & Monserud, 1974; Gates & Westcott, 1978; Pretzsch, 2009). Zones would overlap in ways that simulated better resource allocation and in doing so conspecific effects in P_1_ would be expected to increase and differences in effects between P_1_ and P_2_ to generally strengthen.

The zone of influence concept must be recognized from the outset as a simplistic one in that it assumes that trees in their manner of influencing neighbors were above‐ and below‐ground contiguous matching cylinders (Schwinning & Weiner, 1998; Stoll et al., 2002; Weiner & Damgaard, 2006; Weiner et al., 2001). The notion of similarity of light and nutrient competition strengths is likely not realistic, especially when there are differences between species in root‐shoot allocation ratio and essentially very different mechanisms of competition are involved (Newbery & Lingenfelder, 2017; Newbery et al., 2011).

Crown area has been found to be generally strongly positively correlated with stem diameter in studies of tropical tree architecture and allometry (Antin et al., 2013; Blanchard et al., 2016; Bohlman & O'Brien, 2006; Cano et al., 2019). Zambrano et al. (2019) have recently explored nearest‐neighbor models with crown overlap in relation to functional traits. While above‐ and below‐ground effects will not be independent of one another for structural and physiological reasons, there is no direct evidence in the literature to suggest that lateral spread of root systems mirrors canopy shape and extent. As a start, a ZOI could be envisaged as being made up of many constituent points, symbolizing *plant modules* (branch ends with leaves, coarse and fine roots), so that points within focal trees’ zones, and those of their neighbors, would be at many various distances from one another (Pretzsch et al., 2015; Sorrensen‐Cothern et al., 1993). Crowns would be expected to show some plasticity and to relocate themselves in space to achieve at least maximum light interception (Purves et al., 2007; Strigul et al., 2008). Roots can be also plastic and maybe more so than crowns as they are without mechanical support constraints and are more exploratory in their search for nutrients.

Changing from a “classical” nonspatial to spatial extension models might then be a way to distinguish between the two hypotheses. Spatial extension should lead to the detection of a stronger conspecific effect because any step that let tree size more closely represent the mechanical process of competition would presumably reinforce that effect. This is more immediately obvious when considering crown sizes and light interception: Larger trees with larger crowns would shade larger areas of neighbors than smaller ones. But would this apply in the same way to roots below ground, where root systems of large trees, and their ECMs interlink more often with those of neighbors than do the root systems of smaller trees? Indeed, areas occupied by roots are usually quite heterogeneous in shape, and roots of difference sizes at different distances from trees have differing uptake capacities. A principal difference, therefore, between above‐ground competition for light and below‐ground competition for nutrients is that the former is probably almost entirely asymmetrical in nature and the latter in the main symmetrical (Schwinning & Weiner, 1998; Weiner, 1990).

Under symmetrical competition for resources, uptake and utilization by neighbors are linearly related to their biomass (proportionate), and under asymmetrical competition, they are nonlinearly, normally positively, related to biomass (disproportionate redistribution). These definitions do not exclude competition below ground between roots being slightly asymmetric too under some conditions, though the degree of asymmetry is likely to be far less than that for light above ground as the latter is one‐directional and instantaneous in use and the latter three dimensional and gradual. The two forms of symmetry correspond to the removal of the smaller tree's resources (exploitive nonredistribution) and to relocation of its resources (proportional or shared redistribution) as model modes. Models that fit better with removal form might suggest a predominance of light competition, ones that fit better with relocation, a predominance of nutrient competition. Higher competition above ground will partly translate to higher competition below ground, and vice versa due to root‐shoot inter‐dependencies. On the other hand, a root–shoot allocation strategy and plasticity could counteract that translation. If spatial models in either form failed to improve model fitting, this might question whether competition for resources is at all a reason for the conspecific effects or invoke a search for why the model alternatives were not correctly representing envisaged neighborhood interactions.

If ECMs in general contribute to enhancing root competition, conspecific effects of neighbors under spatial extension models should furthermore be higher for dipterocarps than nondipterocarps, especially under the relocation form—for neighboring trees of similar sizes (basal areas). A mixed range of increases in effects might indicate specialist fungi operating more in favor of some host species than others. If an ECM network operates, it can be postulated that the distance effect will not be mirroring resource depletion curves around trees, but be allowing exploration to much further away. Conspecific effects below ground would presumably operate most strongly in species that are strongly aggregated, not necessarily in that case requiring specialist ECMs; but for dipterocarps that are more spread out they would lack the immediate advantage of high local abundance and ex hypothesis the one way left for them to affect juveniles conspecifically would be through ECMs. Nonaggregated species would be expected to have greater releases in growth rates than aggregated ones, being much freer of adult influences at distance.

### 1.3 Context and modeling aims

In the context of the nearest‐neighbor modeling explored in this paper, terms “spatial” and “nonspatial” refer to the spatial *extension of crowns* around point stem locations. Using crown sizes constitutes a spatial model, using only stem center locations constitutes a nonspatial one. This usage should not be confused with the one of statistical spatial point (pattern) analysis. “Spatial” is a fundamental physical attribute that is applied in numerous contexts.

Unraveling the causal nexus of system interactions (direct and indirect effects, reciprocation and feedback, time‐lagged) is very complicated if the aim is to reduce a phenomenon such as the *average* conspecific effect of a species at population and community levels to a set of understandable mechanisms operating between individuals in space and time (Clark, 2007; Clark et al., 2010, 2011). Conspecific effects, if they are indeed real, and not “by default,” might play a role in determining species composition in forests, but they do not necessarily need to be competitive or facilitative if they come about from a combination of spatial clustering (caused by dispersal) and stochastic environmental (climatic) variability (Newbery & Stoll, 2013).

This third concluding paper on the role of neighborhood effects on tree growth and survival in the lowland rain forest at Danum in Sabah builds directly on Stoll and Newbery (2005) and Newbery and Stoll (2013) by incorporating spatial extension to trees. It attempts to (a) reject the “default hypothesis” for conspecific effects in favor of a resource‐based competition one and, where successful (b), reject the hypothesis that conspecific competition is largely for light in favor of the alternative that it is more for nutrients. This leads to a revision in how negative density dependence is seen to operate in tropical forests and its role in tree community dynamics, as well as a reconsideration of neighborhood stochasticity.

## 2 MATERIALS AND METHODS

### 2.1 Study site

The two permanent 4‐ha plots of primary lowland dipterocarp forest just inside of the Danum Valley Conservation Area (Sabah, Malaysia), close to the middle reaches of the Ulu Segama, are situated *c*. 65 km inland of the east coast of Borneo, at 4°57′48″N and 117°48′10″E. They are at c. 220 m a.s.l.; measure each 100 m × 400 m in extent, lie parallel c. 280 m apart: each samples the lower slope‐to‐ridge gradient characteristic of the local topography. Soils are relatively nutrient‐rich for the region (Newbery et al., 1996, 1999). Rainfall at the site is fairly equitable over the year, totaling c. 2,800 mm on average, but the area is subject to occasional moderate ENSO drought events (Newbery & Lingenfelder, 2004; Walsh & Newbery, 1999).

The plots were established and first enumerated in 1986 (Newbery et al., 1992). Trees ≥ 10 cm girth at breast height (gbh) were measured for gbh, identified, and mapped. The extent of taxonomic naming to the species level was, and has been since then, very high: Vouchers are held at the Sandakan (Sabah) and Leiden (Netherlands) Herbaria. Plots were completely re‐enumerated in 1996, 2001 and 2007. In the present paper, we analyze data of the two longer periods, 1986–1996 (P_1_, 10.00 years) and 1996–2007 (P_2_, 11.07 years). For plot structural data, refer to (Newbery et al., 1992, 1996, 2011). Measurement techniques and their limitations are detailed in Lingenfelder and Newbery (2009). An over‐understory index (*OUI*, continuous scale of 0 – 100), for the 100 most abundant species in the plots, was adopted from Newbery et al., (2011). Three storys are nominally designated as: overstory (OUI > 55), intermediate (OUI 20–55), and understory (OUI < 20).

### 2.2 Species selection

**FIGURE 1:**
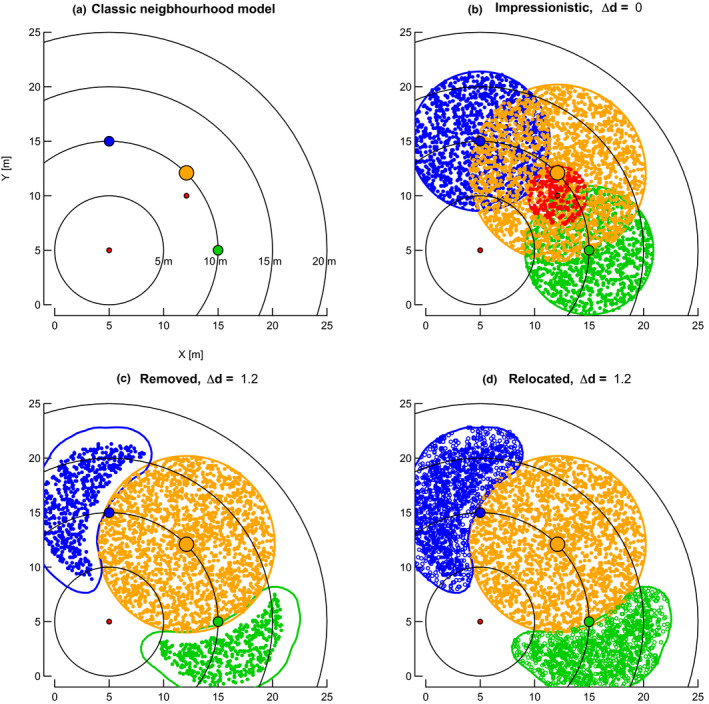
From abstract, mathematical to impressionistic representation of trees with crown plasticity in neighborhood models. (a) Classical neighborhood models represent trees as points without any spatial extension. Taking the red tree at *X*/*Y* = 5/5 as focal tree, it has no neighbors at 5 m neighborhood radius (smallest circle centered at 5/5), four within 15 m and, depending on the exact definition of neighbors (i.e., <10 or ≤10 m), one or four neighbors at 10 m neighborhood radius. (b) Impressionistic representation of tree crowns as circles filled with as many points as the trees basal area at breast height (ba, in cm^2^) and crown radii (cr, in m) allometrically related to girth at breast height (gbh, in cm). Girth of the smallest trees (red at 5/5 and 12.1/10) is 50 cm, those of its neighbors in increasing girth order 175 cm (green at 15/5), 200 cm (blue at 5/15), and 300 cm (orange at 12.1/12.1). These girths correspond to crown radii of 2.7, 5.9, 6.4, and 8.1 m, respectively (all‐species regression, Table 1). All three bigger neighbors of the focal tree at 5/5 have at least parts of their crowns already within the 5 m neighborhood of the smallest one (c and d). Focal tree points may have points of bigger neighbors within their immediate neighborhood as a function of some distance (∆*d*). ∆*d* = 0 would allow complete overlap (as shown in b), whereas larger values of ∆*d* flag individual points (open symbols) as being “shaded” if they have points of bigger neighbors within ∆*d*. Two possibilities are used to handle these shaded points. First (in c), the points are completely removed (*pruned*). Second (in d), in an attempt to mimic plasticity, shaded points are relocated to unshaded parts of the crown using two‐dimensional contour functions (see text) to find the outline of these points. The number of points remains proportional to each tree's ba. The tree at 5/5 is shown here in its role as a focal tree and the other red tree at 12.1/10 (in b) as a conspecific neighbor. This second red tree has no flagged points because ∆*d* = 0. When this second one is taken as a focal tree, the first one would have its crown extended, with possibly some points removed or relocated. The relevant focal tree's position is taken as the stem coordinates (larger colored points) or, alternatively, as the centroid of the unshaded part of its crown (not shown). For the largest tree (orange), these two positions coincide

Of the 37 tree species which had ≥50 small‐to‐medium‐sized trees (10–<100 cm gbh), at 1986 and inside of 20 m borders to the two plots, plus another 11 overstory ones with 20 or more such individuals—48 in all (Newbery & Stoll, 2013), 38 that had five or more dead trees in P_1_, were selected for the analyses here (Appendix S1: Table S1). Among the species excluded was exceptionally *Scorodocarpus borneensis*, with five dead trees, but for which no model for survival could be satisfactorily fitted. Trees in P_2_ were also selected for the same 38 species and size class: Numbers dying in this period were also ≥5. Table S1 of Appendix S1 has the species’ abbreviations which are used later in the Results. Precise locations (to 0.1‐m accuracy) were known for every focal tree and its neighbors, the distance between them being the length of the radius (*r*) of a circle circumscribing the focal tree's location (Figure 1a).

### 2.3 Spatial extension of neighborhood models

Crown radii, cr (in m), and their corresponding girths at breast height, gbh (in cm), were available for 17 species of the Danum plots (F. J. Sterck, personal communication). An allometric relationship was fitted with a linear regression by pooling all of these species’ trees (Table 1). For the most abundant eight species, with *n* > 35 individuals each (Sterck et al., 2001), regression estimates were very similar. These more abundant species were *Aporusa falcifera, Baccaurea stipulata, Mallotus penangensis, M. wrayi, Parashorea melaanonan, Shorea fallax, S. johorensis, and S. parvifolia*, and all occurred in neighborhood analyses reported in this paper. Relaxing the condition of independence for the *X*‐axis (gbh), major axis regression gave slopes very slightly larger than those for standard linear regression (Table 1). Applying the upper regression equation in Table 1 to all trees ≥10 cm gbh in 1986 and in 1996, the predicted total canopy covers were 18.516 and 19.069 ha, respectively (both plots together), which for a land‐surface area of 8 ha represents leaf area indices (or fold‐overlaps) of 2.315 and 2.384. Even trees ≥ 50 cm gbh gave corresponding covers of 9.623 and 10.296 ha. Since cr ∝ gbh^1/2^ and ba = gbh^2^/4*π*, ca ∝ gbh or ca ∝ ba.^1/2^

**TABLE 1:** Regressions between crown radii (cr, in m) and stem girth at breast height (gbh, in cm; square‐root transformed) for 17 tree species at Danum, and the same for eight of these species each with *n* > 35 trees

Number of species	*n*	adj. *R* ^2^	Term	Estimate^a^	*SE*	*t*	*p*(*t*)
17	443	81.4	Intercept	−1.003	0.075	−13.5	<.001
			sqrt(gbh)	0.523	0.012	44.0	<.001
8	382	78.5	Intercept	−0.959	0.085	−11.2	<.001
			sqrt(gbh)	0.516	0.014	36.3	<.001

Note

The data are from of Sterck et al. (2001).

^a^ Major axis (model II) regressions had intercepts and slopes for the two equations as −1.157/0.549 and −1.137/0.547, respectively.

Once the cr of each individual tree in the plots had been determined as a function of its gbh, the (assumed) circular crown was “filled” at random positions with 10 points per m^2^ crown area (ppsqmca: referred to later as “equal”) or, alternatively, as many randomly positioned points as the tree's basal area, ba (in cm^2^). The alternative approach led to larger trees having more points per m^2^ crown area compared to smaller trees (ppsqmca: larger > smaller referred to later as “larsm,” Table 2). The size of each tree in terms of its ba was therefore reflected in the number of points per crown (Figure 1b). Moreover, the alternative approach ensured that the total number of points per plot was identical to the total basal area, Σ ba, per plot (inside of borders), when all species were included, and if no points were removed (see below). Filling was realized by multiplying up the original data file with as many rows per individual tree as points within the crown and initially flagging each point as “uncovered.” Setting cr = 0 for every tree allowed a check of whether the algorithm was working correctly: Such a parametrization corresponds to the nonspatial case, that is, it must give exactly the same results as the nonspatial approach treating individual trees as mathematical points without spatial extension. How the number of points in crowns changes with increasing gbh under the “equal” and “larsm” approaches is illustrated in Table 2.

**TABLE 2:** Illustration of the allocation of points to canopy area (ppca) and per square meter of canopy area (ppsqmca) across a range of tree sizes (gbh—girth at breast height, cr—canopy radius, ca—canopy area, ba—stem basal area) under the two “filling” options: “equal,” with a constant point density per m^2^, and “larsm” where larger crowned taller trees have a disproportionally higher density than smaller lower ones

gbh (cm)	gbh^1/2^	cr (m)^a^	ca (m^2^)	ba (cm^2^)	ppca		ppsqmca		
					equal^b^	larsm^c^	equal	larsm	Ratio
10	3.163	0.645	1.307	7.958	6.5	7.96	10	12	1
30	5.477	1.848	10.73	71.62	18.5	71.6	10	39	4
100	10.000	4.200	55.4	795.8	42.0	796	10	190	19
300	17.321	8.007	201	7162	80.1	7162	10	895	90

^a^ From the equation in Table 1 of main paper: cr = −1.0 + 0.52 gbh^1/2^.

^b^ Based on 10 points m^−2^ of canopy area (3rd column from right).

^c^ Calculated directly as “ba”‐points per canopy.

Different degrees of overlap were realized by visiting each point within every tree's crown and evaluating the point's local neighborhood. If a point in a tree's crown lay within a distance, Δd, of a point of a larger (overlapping) tree's crown, the former was defined as being “shaded” and was flagged. The distances, Δd in steps of 0.2 m, varied from 0.0 (points perfectly overlapping) to 1.2 m (no point of a larger tree's zone of influence within 1.2 m of a smaller trees). Different values for Δ*d* were allowed that corresponded to conspecific (CON) and heterospecific (HET) neighbors in the spatially extended models which involved two terms, that is, Δ*d*_CON_ applied to points of different trees of the same species, and Δ*d*_HET_ to points for different trees of different species. This meant 49 different combinations of the Δ*d* levels on evaluating crown overlap at the start.

Flagged points were then either completely “removed” or they were “relocated” (Figure 1c,d). In the latter case, they were moved to lie within the unshaded part of the crown given by the contour of those points without points of bigger trees crown within Δ*d*; contour function kde2d in R package MASS (R Core Team, 2017–2019; Venables & Ripley, 2010). This procedure attempted to mimic crown plasticity, that is, the tendency of a shaded crown to grow toward higher light availability and more away from being directly under larger shading neighbors. The contours were allowed to be larger than the original crowns by taking the lowest density contour lines as their outer edges.

Spatial extension models provide a test of the hypothesis that asymmetric competition for light, that is, above ground, is the main determining process in tree‐tree interactions at the population and community levels at Danum. If spatial models for growth response to neighbors, especially those that accentuate asymmetry (or nonlinearity), result in stronger relationships with both species plot abundance and with survival response to neighbors than does the nonspatial one, this would confirm to light being the important factor; if not, the inference would be that nutrients below ground using a symmetric competition mode are more important. The R‐code for the calculations of points allocation to crowns, zone of influence overlap, and removal and relocation of points is available on the GitHub Code Repository Platform (www.github.com) site indicated in Appendix S2, together with some technical details and explanation, and a small test data set (Stoll, 2020).

The readjustment of crowns was performed once, across all trees ≥10 cm gbh in the two plots, for each of the Δ*d*_CON_‐x‐Δ*d*_HET_‐level combinations (same seed for each randomization run). It was therefore set for all focal tree neighborhood calculations to follow. When three (or more) crowns were overlapping in a common zone, the largest say A (i.e., the dominant) was considered with the first next largest B (below it) and an adjustment made to B. Then, the second next largest C was considered under the crowns of A and adjusted B. The procedure was therefore hierarchical, in the sense of [A → B] → C, and it left no overlap between the two adjusted crowns B and C. Nonsequential and other procedures would have been possible but they were not explored.

When a small tree was taken as a focal one (i.e., when its neighborhood was evaluated), it was represented without any crown extension: Only its stem coordinates were needed (Figure 1). However, when that same tree was a neighbor to another focal one (of either the same or a different species) it would resume its canopy shape and points distribution, in the way they were set at the start by the universal overlap calculations.

When Δd was 0.0, there was no removal or relocation. This was because the probability of a larger crown's overlapping point coinciding exactly in location with one of a smaller crown below was effectively null (within the limits of real number storage accuracy on the computer). The points might be viewed as being “symmetrical”: The tree is therefore “fully present” in terms of its crown dimensions under Δ*d* = 0.0 (Figure 1b). As Δ*d* increased, though, a rarely occurring distance of 0.2 m could happen by chance, more often so when point densities within the crowns increased (Table 2). This introduced a slight asymmetry. Points were allocated across the circular crowns, just once at random, and each time with the same seed set. (That stage might have been repeated but it would have led to an inordinate increase in computing time, even when say 100 realizations were averaged.) As Δ*d* increased from 0.4 to 1.2 m, more and more flagged points were accumulated when crowns overlapped: The larger the Δ*d*‐value, the more “asymmetrical” was the influence of the larger on the smaller crown because this resulted in more removals or more relocations, and hence points becoming sparser in the shaded crown parts under removal, or becoming denser in the unshaded crown parts under relocation. The total number of points is reduced in Figure 1c, but remains unaltered in Figure 1d: In both cases, the smaller neighbors’ crowns became irregular in shape. If all points of a tree with a small crown became flagged that tree would disappear as a neighbor because its points were either completely removed or had no unflagged crown parts to which they could be relocated.

Once the points of all trees’ crowns had been either removed or repositioned, the neighborhood of each focal tree was evaluated by summing the number of points within a focal tree's neighborhood, a circle with radius *r*, of all larger neighbors (Σba_ALL_), conspecific bigger neighbors (Σba_CON_) or heterospecific bigger (Σba_HET_), each point weighed by a linear distance decay factor (i.e., ba ∙ 1/*r*). Analyses without distance decay (Newbery & Stoll, 2013; Stoll & Newbery, 2005) showed very similar results, and these are not reported here. Summations were evaluated in 1‐m steps for all neighborhood radii (*r*) between 1 and 20 m for focal trees, within the 20‐m borders. To deal with ln‐transformation of zero values, 1 cm^2^ was added to each Σba neighborhood value.

The approach described so far offered, in addition, the possibility of a new way of defining a focal tree's location (or position). Besides, the original field‐recorded stem coordinates, the centroid of the unshaded part of the tree's crown (i.e., mean *x* and *y* values of focal tree points not having any points of bigger neighbors’ crowns within Δ*d*) could be taken as an alternative, perhaps more relevant, location of that focal tree with respect to maximum light availability. In addition to the size (ba‐only) models, and models with either one (Σba_ALL_) or two neighbor terms (Σba_CON_ and Σba_HET_), crown area considerations provided eight combinations from the three spatial extension factors: “equal” or “larsm” for numbers of *ppsqmca*, times “removed” or “relocated” for point adjustment, times “stem” or “crown” location. The adjustment levels will usually be abbreviated hereon to “remov” and “reloc.”

### 2.4 Model fitting

Models for all possible combinations of radii, for CON and HET neighbors, and Δ*d* (see above) were evaluated (i.e., 7 Δd_CON_ * 7 Δd_HET_ * 20 r_CON_ * 20 r_HET_ = 19,600 cases). Least‐squares fits for growth, and general linear models with binomial errors for survival, as dependent variables were then applied for all combinations of radii and Δ*d*, excepting a few cases where fitting was not possible. The approach follows that of Stoll and Newbery (2005) and Newbery and Stoll (2013). The absolute growth rate, agr, of focal trees between two times, *t*_1_ and *t*_2_ was modeled statistically as a function of size at the start of the period (ba*_t_*_1_), and one or two neighbor terms which were sums of ba of trees that survived the period and were larger than the focal one at *t*_1_, as either all (ALL), conspecific (CON) or heterospecific (HET) neighbors weighted by a linear distance decay. Regressing agr upon ba for each species per period, trees that had residuals <–3∙*SD* were iteratively excluded. All variables were ln‐transformed to normalize their errors. The neighborhood models were as follows:

$$\ln\left({\text{agr}}_{t1‐t2}\right)=\text{intercept}+\alpha \ln\left({\text{ba}}_{t1}\right)+\beta \ln\Sigma \left({\text{ba}}_{\text{ALL}}/\text{distance}\right)+\text{error}\;\left(\text{nonspatial only}\right)$$

$$\ln\left({\text{agr}}_{t1‐t2}\right)=\text{intercept}+\alpha \ln\left({\text{ba}}_{t1}\right)+\beta \ln\Sigma \left({\text{ba}}_{\text{CON}}/\text{distance}\right)+\gamma \ln\Sigma \left({\text{ba}}_{\text{HET}}/\text{distance}\right)+\text{error}\;\left(\text{nonspatial}\;\text{and}\;\text{spatial}\right),$$

with intercept, *α*, *β*, and *γ* as the regression parameters to be estimated by the least‐squares approach and normally distributed errors. The summations ba_CON_ and ba_HET_ were evaluated in 1‐m steps for all neighborhood radii between 1 and 20 m, with a border of 20 m. This second model was identical to the C_2_ one of Stoll and Newbery (2005). If less than five focal trees in the sample had CON neighbors, or less than five focal trees had not a single HET neighbor, these model fits were flagged and excluded from further consideration. Their estimates were usually based on, respectively, either very small or very large radii. The magnitude of effects on growth were quantified by calculating effect sizes as squared multiple partial correlation coefficients, or *t* ^2^/(*t* ^2^ + *df*_resid_) (Cohen, 1988; Nakagawa & Cuthill, 2007; Rosenthal, 1994). All models were fitted using alternatively no, linear, and squared distance decay (Stoll et al., 2015).

For survival as dependent variable, the binary response (0/1) was analyzed using generalized linear model with binomial errors (logistic regression), and the same model structures as used for growth. When the proportion of dead trees is small (typically, <0.1), the logit transformation becomes less effective (Collett, 1991), and fitting is unreliable or even fails. For this reason, 10 species (see Section 2.2) were not fully analyzable for both survival and growth as dependent variables. No restrictions regarding numbers of CON and HET neighbors were put in place for the survival models. Some estimates (est) and their associated standard errors (*SE*) were unrealistically very large, and to avoid these cases, estimates with *SE* > 100 were excluded from the calculations of effects of neighbors on focal tree survival.

Survival effects were estimated by the raw regression coefficients, *β*, from logistic regression. The fitted GLM is of the form ln(odds) = *α* + *βX*. Beta therefore expresses the difference in ln (odds) when *X* increases by 1 unit: exp(*β*) is the change in odds, or odds‐ratio, and (exp(*β*) – 1)) × 100 is the corresponding increase or decrease in those odds (Agresti, 2007; Fleiss, 1994; Fox, 2008; Hosmer et al., 2013; Zuur et al., 2007).

### 2.5 Model comparisons

Models were tested and compared by taking a combined pluralistic statistical approach (Stephens et al., 2005, 2007). On the one hand, the classical frequentist approach is needed to assess the strength of model fitting and allow a hypothesis‐testing framework (recently defended by Murtaugh, 2014; Spanos, 2014), while on the other hand, the information‐theoretic approach provides an efficient means of model comparison and inter‐model summarization (Burnham et al., 2011; Richards et al., 2011), with the final outcome being purely relative yet avoiding “data‐dredging” and undue heightening of confidence through multiple testing that contravenes the rules of independence (Symonds & Moussalli, 2011). The formulation of good alternatives to the null hypothesis may lead to more informed model fitting and testing than when none are posed beforehand (Anderson & Burnham, 2002; Burnham & Anderson, 2001). Several cautionary points have been raised in the literature concerning the general use of the information‐theoretic approach (see Arnold, 2010; Richards, 2005). Especially, it does not sit well on its own within a critical rationalist approach to science. It provides for a valuable heuristic complement, however.

The analysis here was therefore a mixture of approaches, structured as follows. First, the central reference model is just tree size (gbh), plus the basal area (ba) of “ALL” neighbors’ basal area within radius *r*. The question was whether model fits were improved by having CON and HET terms in place of “ALL,” and then having fixed spatial terms for them (eight alternatives). The differentiation between CON and HET constitutes one quantum‐level change in information and the addition of spatial form a second. These eight spatial forms were not fully independent of one another in their information because CON and HET ba‐values will be highly correlated. The modes of decay (the inverse distance weighting applied to neighborhood BA) offered three different ways to improving model fitting. Accordingly, the reference model, ex hypothesis, for within periods 1 and 2 and for growth and for survival, was the nonspatial “ba + ALL” one with linear decay. It may not have necessarily been the best‐fitting model compared with the other nonspatial and spatial ones. Individual species’ best‐fitting models were said to differ strongly from the reference model when the ΔAICc was >|7|, and to be not different when ΔAICc was ≤7 (Burnham & Anderson, 2010). A ΔAICc‐value of 7 or one more negative meant a “much better” model, one of 7 or more positive, a “much worse” one. ΔAICc > |7| is equivalent to a Pearson–Neyman significance level of *p* ≤ .003–.005 (with *k* = 1 to 4 independent variables; Murtaugh, 2014).

The dependence of CON effects for growth or survival at the community level (one point for each of the 38 species) on total plot BA per species was estimated again by standard linear regression. Because models with ΔAICc in the 2–7 range have some support and should perhaps not be too readily dismissed (Burnham et al., 2011; Moll et al., 2016), results and estimates from all different nonspatial and spatially extended models are reported. Regression statistics from specific models but different neighborhood radii or Δ*d* were often very similar and had very small ΔAICc among them. The correlations between differences in the CON or HET effect sizes on growth between periods (P_2_–P_1_) and CON or HET effect (expressed as raw coefficients) on survival in P_1_ or P_2_ were tested at the community level with the expectations stated in the Introduction.

The final effect sizes, for a nonspatial or spatial model, per species and period, were found by averaging raw coefficients (equally weighted) across all radii and Δ*d* values with fits ≤2 ΔAICc of the best one, that is, the one with the smallest AICc (Claeskens & Hjort, 2008; Ripley, 2004). Averaging was considered valid here because all of the models involved had exactly the same structure (same terms), and so within species and period, they would be differing in the exact combination of *r*_CON_, *r*_HET_, Δ*d*_CON_, and Δ*d*_HET_ values used (see Banner & Higgs, 2017; Cade, 2015, for general discussion). Averaging was unweighted, that is, no Akaike weights, *w_i_*, were applied since there was no a priori reason to do so within such a small AICc‐band (Burnham & Anderson, 2001, 2010). There were often very many models in this 2‐ΔAICc range, and in some cases, there was a change in sign for a minority of them; *r* and Δ*d* values were often very close to one another. Alternative ways of summarizing these coefficients, namely averaging only those values with sign the same as that of the overall mean, or taking the medians, resulted in very small differences in the overall outcomes, and hence, the simple arithmetic mean was used.

Calculations were performed largely in R (version 3.4.2; R Core Team, 2017–2019), using package AICcmodavg (Mazerolle, 2019) to find AICc, the small sample size correction of AIC, Akaike's information criterion (Burnham & Anderson, 2010; Hurvich & Tsai, 1989). Predicted *R* ^2^‐values were found using the predicted residual error sum of squares (PRESS) statistic (Allen, 1974; Fox & Weisberg, 2011). The calculation of (pseudo‐) *R* ^2^ for logistic regression followed (Mittlböck & Schemper, 1996), where *R*_L_^2^ = [(*L*_0_ − *L*_p_)/*L*_0_] ∙ 100, *L*_0_ and *L*_p_ being the log‐likelihoods of the model with only the intercept and with the nearest‐neighbor (spatial) terms, respectively (Hosmer et al., 2013, p. 184). The *R*_L_^2^‐values were adjusted as in linear least‐squares regression, although they are not directly comparable.

### 2.6 Randomizations

To more rigorously test the significance of the CON and HET coefficients, the model fitting was re‐run for *n’* =100 randomizations of locations of trees within the plots. The randomization outcomes of Newbery and Stoll (2013) were used. The method that produced them is described in detail in Appendix B (ibid.): It involved simple rules allowing different minimum distances between nearest‐neighbor trees within the same and different size classes (six defined), and it ensured that the same overall frequencies of size distribution for each species were maintained. On each run, focal trees were those, of each species (in the size class used for the observed trees), which were now located within the 20‐m plot boundaries: CON and HET neighborhoods were accordingly realistically randomized; any spatial clustering in observed tree distributions will have been removed as well. The procedure also tests whether the relationships in the community‐level graphs might have arisen by chance.

## 3 RESULTS

### 3.1 Finding the best fit models

Frequency distributions of growth and survival CON (raw) coefficients within 2ΔAICc, for each of the 48 species first analyzed in P_1_ and P_2_ (survival in P_1_ gave 46 histograms) using the nonspatial and the spatial “larsm/reloc/crown” and “larsm/remov/crown” modes, were inspected visually for evidence of obvious bimodality, or multimodality, which would indicate inconsistency in the final averages estimated (Appendix S3). Bimodality was judged to be present when there were two clear modes separated by being to either side of zero or otherwise by a peak difference at least approximately twice the mean coefficient. Of the 570 cases, 16 (2.8%) showed evidence of bimodality (seven “±‐zero,” nine “≥2‐fold difference”). There were no cases of multimodality. Repeating this analysis with growth and survival, HET coefficients, but for just the spatial “larsm/reloc/crown” mode, just five of 190 cases (2.6%) were correspondingly bimodal (two “±‐zero,” three “≥2‐fold difference”). For both CON and HET coefficients, bimodal cases were occurring across many different species and not the same for different modes or periods. Different peaks were arising because models were fitting at two clusters of similar radii (and Δ*d* values) suggesting that occasionally two neighborhood relationships may have been operating. Overall, these cases are too infrequent to have affected the main results to any major degree.

A particularly interesting feature is that for nonspatial models many species had over 100 (out of the maximum of 400 possible), and for spatial models thousands or tens of thousands (out of 19,600 maximally), CON effect estimates within the 2ΔAICc‐band. This latter maximum was actually reached for *Pentace laxiflora* (“larsm/reloc” and “larsm/remov”) growth in P_2_, and for *Dehassia gigantifolia* (“larsm/reloc”) survival in P_1_. It means that many models were indistinguishable in their estimates of CON effects, and that radius or Δ*d* interacting with ba had little role, and presumably the main information lay in the presence or absence of any neighbor within 20 m of the focal tree.

### 3.2 Individual species’ model fits

With linear distance decay and any of the eight spatial model forms, having “ba + CON + HET” as the terms for growth responses in P_1_ led to 8–14 out 38 species (on average 29%) showing better fits than when using just “ba + ALL” terms. No particular model excelled by being better fitting for appreciably more species, although for four of them (11%) the fits were better by just using “CON + HET” without spatial extension (Appendix S1: Table S2a). For P_2_, the outcome was similar but slightly weaker in that 7–14 species (28%) had correspondingly better fits. No‐decay models were similarly frequent to linear distance ones, although squared distance models were fewer in both periods. The number of species which had worse fits when including spatial extension, compared with “ba + ALL,” were very few in P_1_ (0–2), and slightly more for P_2_ (1–3).

Spatial models for survival responses led to very few species with improved fits in P_1_ and P_2,_ for linear distance decay 2–5 (8%) and 2–7 (12%) out of 38 species, respectively (Appendix S1: Table S2b). Replacing the model terms "ba + ALL" by "ba + CON + HET" resulted in 0–1 species with improvements in P_1_ and P_2_: The corresponding number of species with worsening fits when comparing spatial models with “ba + CON + HET” was 0–1 in both P_1_ and P_2_ (Appendix S1: Table S2b). Tables of nonspatial and the two spatial, “larsm/reloc/crown” and “larsm/remov/crown,” model parameter fits for all species, for growth and survival, in P_1_ and P_2_ are linked in Appendix S4.

Resetting the reference model to “ba + CON + HET” instead of “ba + ALL,” for spatial linear decay modes, the number of species with improved fits decreased to 3–10 (17%) for P_1_ and 4–11 (20%) for P_2_, especially “larsm” models the reduction was down to 3–4 for P_1_ and 4–6 for P_2_ better fitting (Appendix S1: Table S3) while just 0–1 and 1–3 species with “larsm” models were, respectively, worse than the reference one. These comparisons imply that part of the improved model fitting under spatial extension compared with “ba + ALL” was because CON and HET were being used as separate terms.

Considering the individual species’ fits in P_1_ and P_2_, over the nonspatial and two spatial “larsm/reloc/crown” and “larsm/remov/crown” models, for growth, 9–13 (29%) of species had adjusted *R* ^2^‐values ≥50% and only 8–12 (26%) <20% (Appendix S1: Table S3); and for survival—recalling here that *R* ^2^ is a “pseudo”‐estimate—far fewer at 0–2 (3%) had *R* ^2^‐values ≥50% and as many as 33–35 (89%) of species with just <20%. The *p*‐Values of CON coefficients were ≤0.05 for 9–22 (41%) for growth and 6–11 (22%) for survival; 7–16 (30%) and 13–21 (45%) with *p* ≥ .25. HET coefficients showed similar distributions, for growth 15–22 (49%) at *p* ≤ .05 and for survival 4–8 (16%) of species, with correspondingly 8–13 (28%) and 14–21 (46%) at *p* ≥ .25 (Appendix S1: Table S4). In general, the significance of fits and their coefficients were weaker for survival than growth regressions, but the CON and HET coefficients’ *p*‐values were rather similar. Adjusted *R* ^2^‐values were similar for the nonspatial and two spatial models of more interest, though CON coefficients were more often significant (*p* ≤ .05) for spatial than nonspatial, and HET coefficients showed a slight trend in the opposite direction (Appendix S1: Table S4).

### 3.3 Effect sizes dependence on species’ plot basal area abundance and density

Regressing the 38 species’ CON effect sizes on growth in P_1_, whether at the individual species’ level they were significant or not, against plot BA (log_10_‐transformed), showed that the nonspatial model with “ba + CON + HET” led to a substantially better fit (*p* ≤ .001) than with “ba + ALL” (*p* = .18) (Table 3a) and accounted for the maximum adjusted and predicted *R* ^2^ of all nonspatial and spatial models. However, including spatial extensions with the eight different forms led to reduced fits, rather surprisingly, with adjusted and predicted *R* ^2^ decreasing by about a third (and *p* ≤ .01). The eight spatial forms differed little from one another in fit although “equal” was slightly better than “larsm.” In P_2_, the relationships were similar but less strong and less significant, the nonspatial “ba + CON + HET” model achieving significance only at *p* ≤ .05. Predicted *R* ^2^‐values were very low, much lower than the adjusted values for the eight spatial forms (Table 3a). In P_1_ and in P_2_, the slopes of the relationships changed little between nonspatial and spatial modes, and if at all were slightly less negative for the spatial ones (Table 3a). To recall, the stronger the CON or HET effect the more negative it was, so if the species’ values decreased with increasing plot BA the expected slope of the relationship would be negative.

**TABLE 3:** Dependence of conspecific (CON) effects in terms of (a) absolute growth rate, and (b) survival in periods 1 and 2 (P_1_, P_2_), and (c) difference in CON effect sizes in growth rates between periods (P_2_ − P_1_), of the 38 species, for the different nonspatial and spatial models using linear distance decay, on plot‐level basal area (as log_10_[BA])

Model	ppsqmca	Cover	Position	Period 1				Period 2			
				Adj. *R* ^2^	*p*	Pred. *R* ^2^	Slope^a^	Adj. *R* ^2^	*p*	Pred. *R* ^2^	Slope^b^
(a) Growth											
ba + ALL	–	–	–	2.4	.177	−8.7	−0.034	1.9	.197	−6.7	−0.027
ba + CON + HET	–	–	–	35.2	<.001	23.4	−0.120	9.3	.036	1.2	−0.046
	Equal	Removed	Stem	21.9	.002	14.7	−0.115	10.0	.030	−0.9	−0.061
			Crown	22.2	.002	14.6	−0.115	11.8	.020	2.1	−0.064
		Relocated	Stem	21.0	.002	12.8	−0.115	12.1	.019	1.7	−0.068
			Crown	21.8	.002	12.8	−0.113	12.1	.018	2.6	−0.066
	Larger > smaller	Removed	Stem	19.9	.003	12.7	−0.103	9.6	.033	−0.4	−0.057
			Crown	18.2	.004	10.8	−0.097	13.2	.014	4.8	−0.065
		Relocated	Stem	16.6	.006	9.6	−0.102	10.8	.025	1.1	−0.067
			Crown	15.4	.008	8.0	−0.096	12.2	.018	3.0	−0.070
(b) Survival											
ba + ALL	–	–	–	−2.2	.662	−7.2	0.128	1.5	.219	−4.3	−0.406
ba + CON + HET	–	–	–	4.0	.118	−7.3	0.339	16.4	.007	9.0	−0.332
	Equal	Removed	Stem	−0.1	.332	−6.4	0.354	11.3	.022	5.7	−1.085
			Crown	2.5	.170	−6.3	0.695	15.4	.008	8.4	−1.306
		Relocated	Stem	3.3	.142	−4.9	0.438	13.8	.012	7.6	−0.628
			Crown	3.6	.131	−4.6	0.462	18.8	.004	8.0	−0.867
	Larger > smaller	Removed	Stem	1.5	.218	−5.8	0.575	7.5	.053	1.8	−0.999
			Crown	2.2	.183	−5.0	0.626	8.6	.042	2.7	−1.149
		Relocated	Stem	2.5	.171	−4.2	0.466	6.6	.065	1.2	−0.423
			Crown	3.5	.136	−3.4	0.516	12.2	.018	2.5	−0.596

Model	ppsqmca	Cover	Position	Period 1 and 2			
				Adj. *R* ^2^	*p*	Pred. *R* ^2^	Slope^a^
(c) Difference in growth							
ba + ALL	–	–	–	−2.8	.931	−9.6	0.002
ba + CON + HET	–	–	–	13.7	.013	−4.7	0.072
	Equal	Removed	Stem	3.0	.153	−10.5	0.050
			Crown	2.2	.186	−11.0	0.045
		Relocated	Stem	1.1	.241	−15.4	0.041
			Crown	1.3	.232	−15.5	0.041
	Larger > smaller	Removed	Stem	1.6	.215	−12.3	0.042
			Crown	−0.5	.378	−13.1	0.028
		Relocated	Stem	−0.8	.405	−14.5	0.030
			Crown	−1.7	.546	−15.2	0.021

Note

The models involved basal area (ba) and one or two neighborhood terms and, in the spatial case, one of the eight different forms of crown extension (as structured in Table 2). The final effects sizes came from model averaging, that is, finding mean CON coefficients across all model fits ≤ 2 ΔAICc of the best‐fitting one.

(a) Ranges in *SE*: ^a^0.025–0.035; ^b^0.020–0.029; (b) Ranges in *SE*: ^a^0.212–0.496; ^b^0.116–0.543. (c) Ranges in *SE*: ^a^0.028–0.035.

Considering the 38 species’ CON effects on survival in P_1_, regressions for both nonspatial and spatial forms were very weakly dependent on plot BA (*p* = .13 to .33 for the spatial ones). However, the relationships here were stronger in P_2_ than P_1_ and showed improved fits for spatial forms (*p* < .05 in all but two cases) over nonspatial ones, *R* ^2^‐values reaching almost as high as those found for growth in P_1_ (Table 3b). The slopes of the relationships were positive in P_1_ and negative in P_2_, becoming steeper for spatial compared with nonspatial modes, despite the lack of significance (Table 3b). Difference in CON effect size on growth P_2_ − P_1_ (i.e., effect size and P_2_ minus that at P_1_) regressed on plot BA had much lower adjusted and predicted *R* ^2^‐values than for CON effects on growth in P_1_ and P_2_ separately, and most notably the nonspatial model with “ba + CON + HET “was far poorer fitting (Table 3c). None of the eight spatial modes had significant fits (i.e., *p* ≥ .15). Slopes for these differences in CON effect size were all positive but less so for spatial than nonspatial modes.

Of the nine spatial and nonspatial model forms times eight “CON‐HET versus growth‐survival versus P_1_–P_2_” combinations (72 in all), correlations between effect sizes (for growth), or raw coefficients (for survival), and log_e_ (population size), all were weak and insignificant except for HET effect on growth in P_1_ across all eight spatial model forms was consistently positive (*r* = .433 to .500, *p* ≤ .005). Variables were all approximately normally distributed except HET effect on growth in P_2_ with one distinct outlier.

### 3.4 Cross‐correlations between the eight spatial models

For each of the eight CON‐HET x growth survival x P_1_‐P_2_ combinations there were, among the 28 pair‐wise correlations of the eight different spatial models (38 species selected), several‐to‐many showing very high agreement (*r* = .96 to >.99; Appendix S1: Table S5). Differences arose as the correlations between models became weaker. For CON growth P_1_ and P_2_ which spatial model form was used had little influence as the correlations were always very high. For the corresponding HET growth P_1_ and P_2_, the minimum *r*‐values (and corresponding *t*‐values) decreased moderately, especially for “larsm/reloc” versus “equal/remov.” In comparison to growth based variables, correlations between spatial models based on survival variables dropped considerably, especially for “larsm/reloc” versus “equal/remov.” Crown or stem location (or position) accounted very little for differences between spatial models. Growth models, therefore, depended very little on the spatial form, but survival models so did much more. Spatial extension was apparently influencing survival more than growth across species, for CON and HET cases.

### 3.5 Radii of neighborhood effects

For the “larsm/reloc/crown” model, across the 38 species, mean of mean model‐fitted radii were similar for growth and survival CON and HET coefficients at P_1_ (9–10 m). By P_2_ CON mean radii exceeded HET ones for both growth and survival (Table 4), CON and HET means being on average closer to 10 m—midway for the radii modeled (viz. ≤20 m). The weaker CON effects in P_2_ than P_1_ is commensurate with increasing mean radius. Mean radii were based in some cases on very many model fit estimates where radii ranged greatly, and radii were not normally distributed for every species. Nevertheless, mean range of radius, over which effects were averaged, was clearly smaller for CON than HET, for both growth and survival at P_1_ (10 vs. 13–14 m), but by P_2_ these were much more similar at 11 m for growth and 13 m for survival (Table 4). The lessening of CON coefficients from P_1_ to P_2_, and their moving toward the HET ones, occurred as radii became more similar.

**TABLE 4:** Means of means (±*SE*) and of ranges of radii within the models fitted within 2ΔAICc, across the 38 species, and the means of the corresponding regression slopes of CON (conspecific) or HET (heterospecific) coefficients on radii, for the growth and survival response variables in periods P_1_ and P_2_, using spatial model “larsm/crown/reloc”

	Growth		Survival	
	CON	HET	CON	HET
Mean of radius mean (m)				
P_1_	9.85 ± 0.83	9.55 ± 0.86	9.99 ± 0.78	8.99 ± 0.74
P_2_	13.30 ± 0.72	7.86 ± 0.91	11.11 ± 0.85	7.98 ± 0.72
Mean of radius range (m)				
P_1_	9.87 ± 1.15	13.16 ± 1.17	10.03 ± 1.11	14.47 ± 1.04
P_2_	10.74 ± 0.99	10.89 ± 1.34	13.16 ± 1.08	13.03 ± 1.11
Mean slope of estimate on radius (m^−1^·10^3^)				
P_1_	10.31 ± 4.13	−20.09 ± 4.79	0.00 ± 52.7	6.59 ± 8.61
P_2_	6.14 ± 2.27	−9.04 ± 3.15	24.8 ± 12.1	0.30 ± 16.70

Across the 38 species, again for the same spatial model, and using the raw coefficients (i.e., not effect sizes for growth), mean CON coefficient was positively correlated with mean CON radius at which the effect operated for growth in P_1_ (*r* = .545, *p* ≤ .001) and P_2_ (*r* = .370, *p* ≤ .05), and survival in P_1_ (*r* = .208, *p* > .05) and P_2_ (*r* = .436, *p* ≤ .01), although mean difference in CON coefficients for growth in P_2_–P_1_ were not significantly correlated with mean CON radii for growth in P_1_ and P_2_ (*r* = −.137, *p* > .05). In contrast, mean HET coefficient was weakly (*p* > .05) negatively correlated with mean HET radius for growth in P_1_ (*r* = −.096) and P_2_ (*r* = −.248), and survival in P_2_ (*r *= −.169), although survival in P_2_ showed a correspondingly much stronger positive correlation (*r* = .509, *p* ≤ .001), and mean difference in HET coefficients for growth in P_2_–P_1_ was also not significantly correlated with mean HET radii for growth in P_1_ and P_2_ (*r* = −.030, *p* > .05). Thus, species with strong negative CON coefficients for growth tended to be operating at short distances, and the strong positive ones at much larger distances (≤20 m). Even so, differences in CON growth coefficients P_2_–P_1_ across species were less related to neighbor distance than those for P_1_ and P separately.

Correlations between CON and HET regression coefficients, growth and survival, P_1_ and P_2_, with best‐fitting radii within the 2ΔAICc range were also found. Histograms of the 38 species correlation coefficients revealed a clear difference between CON and HET: The former were always bimodal, with strong negative and positive correlations, and the latter were normally distributed around zero, that is, most species were weakly or not correlated with radius (Appendix S1: Fig. S1a). This would reflect the more species‐specific nature of CON effects (by definition) versus the highly mixed and diverse ones bundled into HET effects. The difference values will have been very highly spatially autocorrelated as they were using neighboring ppsqmca locations. A change in radius increments across the defined neighborhood crowns (see Figure 1), so different combinations of points would be achieved as different neighbor's crowns are encompassed.

Regressions for all of the 38 species studied, again for growth and survival, P_1_ and P_2_, estimated the changes in CON or HET coefficient per meter of radius (Table 4). For ca. 90% of the species, the slope values were very small. Graphs of coefficient versus radius (Appendix S5) indicated mostly continuous set of lightly curved lines, increasing or decreasing with radius; occasionally there was a mixture of lines resulting in little overall trend, rarely disjunctions (five cases of 1–2 m). Histograms of the slopes for coefficient versus radius did highlight, however, a few strongly positive or negative outliers, especially for CON survival in P_1_ (Appendix S1: Figure S1b). The five important species’ cases that might have biased the study's conclusion are highlighted. Strong bimodality explained some of them because when the tail values formed a minority of points, basic assumptions of regression were not being met. Only very small shifts in mean CON effects on survival in P_1_ occurred on the exclusion of tail values.

### 3.6 Crown overlap and readjustment

Correlations between mean Δ*d*_CON_ and mean Δ*d*_HET_ (across the model fits within 2ΔAICc, as for coefficients and radii) for growth and survival in P_1_ and in P_2_, for “larsm/crown/reloc” were all very weak and insignificant (*r* = −.077 to .034, *p* ≥ .65). Likewise, for either Δ*d*_CON_ or Δ*d*_HET_ between growth and survival, in P_1_ and in P_2_, correlations were weak (*r* = −.240 to .057, *p* ≥ .15). Using “larsm/crown/remov” as the model gave very similar outcomes.

Mean Δ*d*_CON_ and Δ*d*_HET_ values across the 38 species (with “reloc”) were nevertheless very similar and all sitting near the center of the levels 0 to 1.2 m preset (Table 5): The overall average was 0.524 (range 0–1.2). Means for survival were slightly higher than those for growth. Means using “remov” in the model were also very close to those with “reloc.” Histograms of the 38 species’ mean Δ*d* values, for the different combinations of CON/HET, P_1_ and P_2_ and growth/survival, were all either roughly even or slightly normally distributed, in the full range 0 to 1.2, but none were skewed (Appendix S1: Fig. S2). The expected possible separation then of models fitting best with Δ*d* = 0 versus those with Δ*d* > 0—recalling the important qualitative difference and its consequence—was not obvious.

**TABLE 5:** Means of means (±*SE*) of Δ*d* values, with ranges in parenthesis, within the models fitted within 2ΔAICc, across the 38 species, for the growth and survival response variables in periods P_1_ and P_2_, and using spatial model “larsm/crown/reloc”

	Growth		Survival	
	CON	HET	CON	HET
P_1_	0.494 ± 0.057	0.485 ± 0.044	0.573 ± 0.047	0.602 ± 0.034
	(0 – 1.20)	(0 – 1.01)	(0 – 1.16)	(0 – 1.00)
P_2_	0.527 ± 0.044	0.446 ± 0.047	0.519 ± 0.045	0.549 ± 0.040
	(0.09 – 1.20)	(0 – 0.99)	(0 – 1.20)	(0 – 0.98)

Abbreviations: CON, conspecific (Δ*d*_C__ON_); HET, heterospecific (Δ*d*_HET_).

Despite these overall community‐level means being so similar and central, species differed individually from one another markedly in the distributions of their Δ*d*_CON_ and Δ*d*_HET_ values for the model fits, across the full 0‐to‐1.2 range. Some had best fits with only Δ*d*_CON_ or Δ*d*_HET_ = 0, or alternatively 1.2, others had low‐ or high‐valued skewed peaks, and several showed clear declines from, or inclines toward, the scale extremes. The “boxes” defined by Δ*d* 0–1.2 for CON and HET were largely, and approximately evenly, filled with points of the species means; and hence the very poor correlations noted. Visually matching species’ histograms of Δ*d*_CON_ values for models using growth versus those using survival, both in P_1_ (“reloc”)—and the same for Δ*d*_HET_ (38 × 2 = 76 combinations), 30 showed a strong tendency for Δ*d* to be low or 0.0 for growth yet high or at 1.2 for survival, and 14 the converse, that is, 58% of cases were radically opposite in most frequently fitted Δ*d* values for the two responses. Once more, the models with “remov” barely differed from “reloc,” having very similar patterns.

Mean Δ*d*_CON_ and Δ*d*_HET_ values for the 38 species were, furthermore, not strongly or consistently related to the CON and HET effect sizes in the best‐fitting models either, when again placing attention on growth and survival responses in P_1_ and in P_2_ (the “reloc” model). Seven of eight correlations were insignificant (*p* > .05), and the one for CON/survival/P_2_ only marginally so (*r* = −.331, *p* = .043). With “remov” in place of “reloc” in the model, a different period was significantly highlighted, as CON/survival/P_1_ (*r* = −.396, *p* = .014). Hence, CON and HET effect sizes were seemingly unrelated to degree of overlap of crowns (ZOIs). Correlations of species’ mean Δd_CON_ and Δd_HET_ values and plot‐level BA were poor too (*r* = −.160 to .097, *p* ≥ .34).

### 3.7 Effect sizes on growth and effects on survival

**FIGURE 2:**
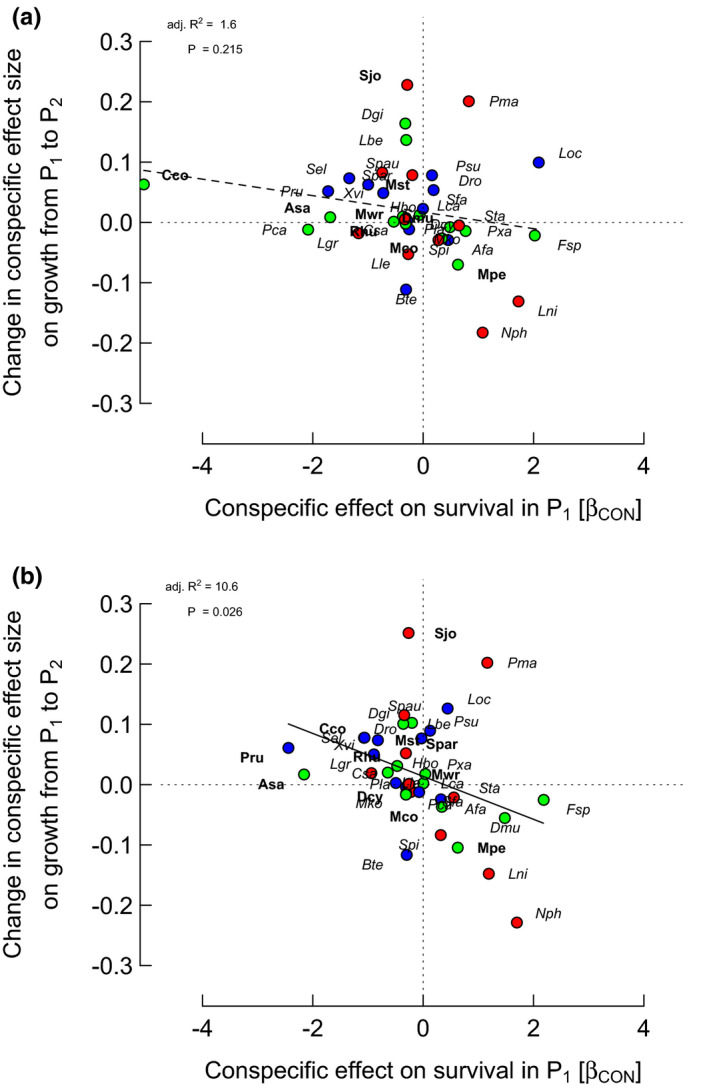
Relationships between *differences* in conspecific (CON) effect sizes on growth rates between periods (P_2_ − P_1_) and CON effects on survival in period 1 (P_1_; *β*_CON_ = change in log[ODDS] per unit change in log(1 + sum(CON_ba_))) for the 38 species, from spatially extended neighborhood models with size (ba) and two neighbor terms (HET and CON). Points of focal trees having points of bigger neighbors within their zone of influence were either (a) removed or (b) relocated. Crown position was used as focal tree position in order to evaluate its neighborhood (lines 8 and 10 in Table 3a refer). Color codes for points. OUI, over‐ understory index: <20 (green), ≥20–55 (blue), >55 (red). Species’ labels: italicized, CON effect on survival in P_1_ with *p* ≥ .05, in bold *p* < .05. Points (each representing a single species) above or below the horizontal dotted line (*Y* = 0) indicate, respectively, decrease or increase of CON effects on growth, going from P_1_ to P_2_. Points to the left or right of the vertical dotted line (*X* = 0) indicate, respectively, negative or positive CON effects on survival (CON neighbors increasing or decreasing mortality)

**FIGURE 3:**
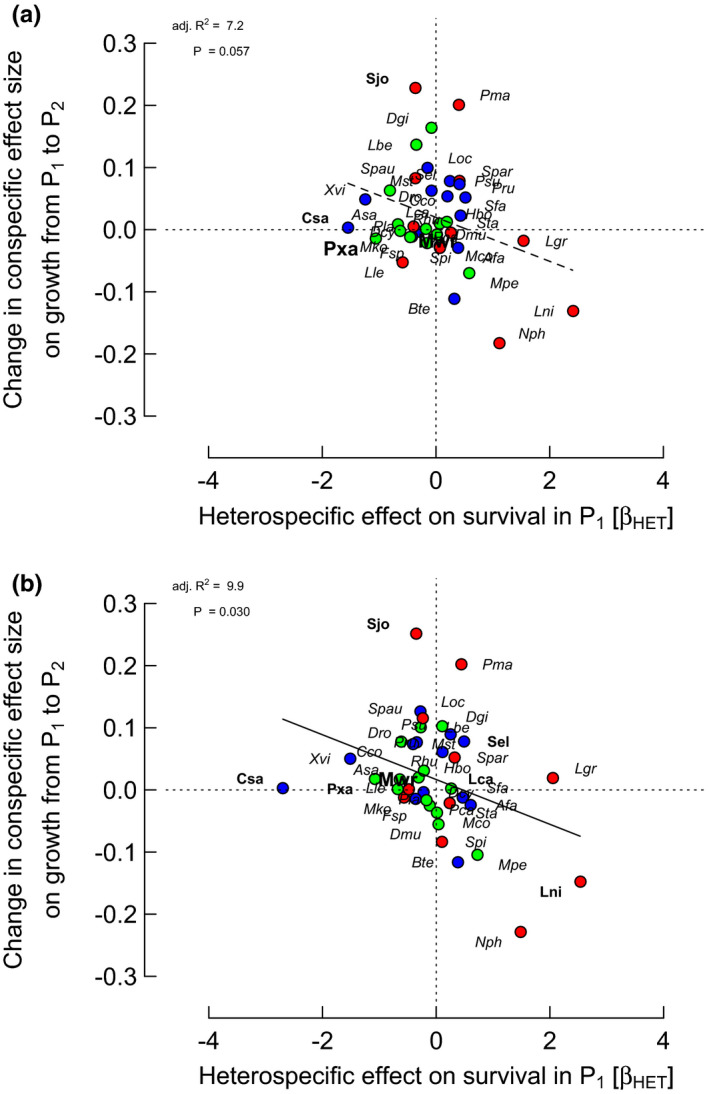
Relationships between *differences* in conspecific (CON) effect sizes on growth rates between periods (P_2_ − P_1_) and HET effects on survival in period 1 (P_1_; *β*_HET_ = change in log[ODDS] per unit change in log(1 + sum(HET_ba_))) for the 38 species, from spatially extended neighborhood models with size (ba) and two neighbor terms (HET and CON). Points of focal trees having points of bigger neighbors within their zone of influence were either (a) removed or (b) relocated. Details are the same as for Figure 2, except for species’ labels: larger font, HET effect on survival in P_1_ with *p* < .001

The absolute values of the effect sizes on growth, as squares of the partial correlation coefficients, are proportions of the total model variance (*R* ^2^) accounted for in each species’ fitting. Squared partial correlations (e.g., CON) are proportions of the variance in Y that is unaccounted for by the other variables (ba and HET) in multiple regression, that is, when these other variables are set constant (at their means) and have no variance. By contrast, semi‐partial or part correlations squared would express the proportion of variance in Y accounting for all variables in the model (overall model *R* ^2^; Cohen, 1988, Warner, 2013). For the two periods, CON and HET, and for the two spatial models “larsm/reloc/crown” and “larsm/remov/crown,” half of the species defined by the eight medians had variances of just 2.7 to 4.4% or less, the upper quartiles reaching 7.2 to 13.0%, and just a very few species attaining >20%. It is these few that give the most leverage to the relationships at the community level. Fits with relocated crowns were slightly better overall than with removed crowns. These variances, in an approximately similar order of magnitude, are realized by the spread of differences in CON effects on growth in Figures 2 and 3 (signs reassigned).

### 3.8 Community‐level graphs

The strongest correlations were between CON difference effect on growth P_2_–P_1_ and CON or HET effect on survival in P_1_ for “larsm/reloc/crown” with *r* = −.361 and −.352, respectively (*p* = .026 and *p* = .030; Figures 2b and 3b). Those corresponding for “larsm/remov/crown” were weaker, with *r* = −.206 and −.311 (*p* = .215 and .057; Figures 2a and 3a). CON difference effect on growth on the sum of CON and HET effects on survival in P_1_, however, showed an even stronger correlation for “larsm/reloc/crown” with *r *= −.437 (*p* = .006), although rather less strongly for “larsm/remov/crown” with *r *= −.296 (*p* = .071). Correlations between CON difference effect on growth P_2_–P_1_ and CON, or HET, effect on survival in P_2_, however, were very poor with a range in *r* = −.023 to .055 (*p* = .74 to .89); and likewise the sum of CON and HET effects versus survival in P_2_, for “larsm/reloc/crown” and “larsm/remov/crown” were very weak (*r *= −.014 and .034; *p* = .93 and .84).

**FIGURE 4:**
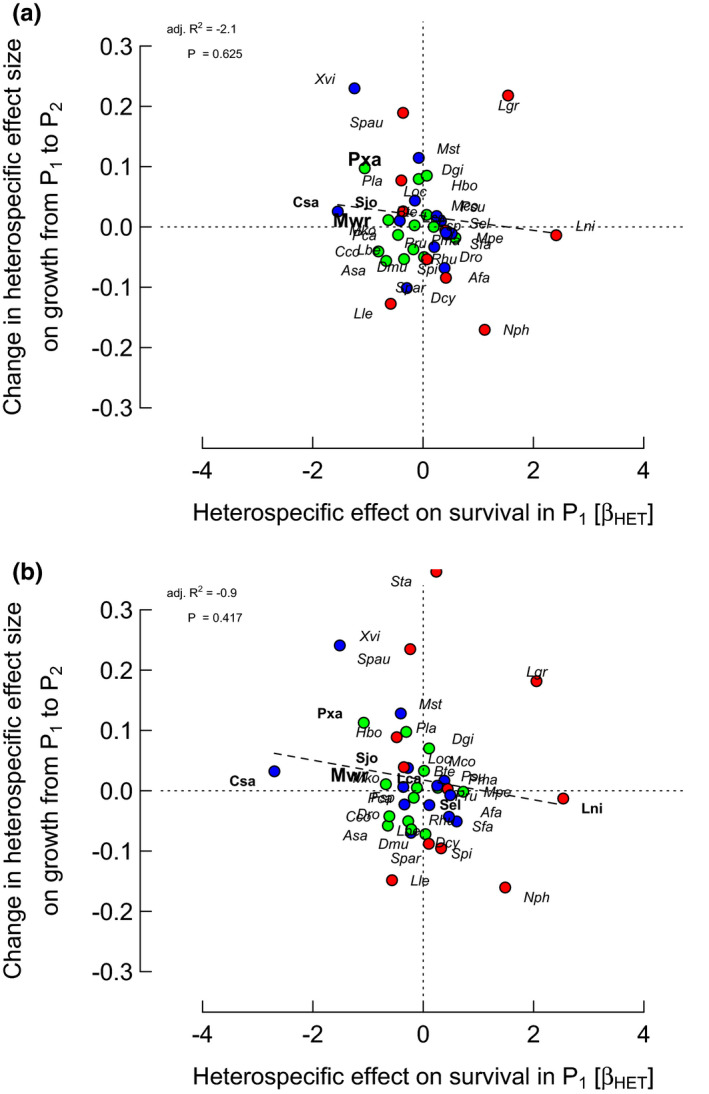
Relationships between *differences* in heterospecific (HET) effect sizes on growth rates between periods (P_2_ − P_1_) and HET effects on survival in period 1 (P_1_; β_HET_ = change in log[ODDS] per unit change in log(1 + sum(HET_ba_))) for the 38 species, from spatially extended neighborhood models with size (ba) and two neighbor terms (HET and CON). Points of focal trees having points of bigger neighbors within their zone of influence were either (a) removed or (b) relocated. Details are the same as for Figure 2, except for species’ labels: larger font, HET effect on survival in P_1_ with *p* < .001

**FIGURE 5:**
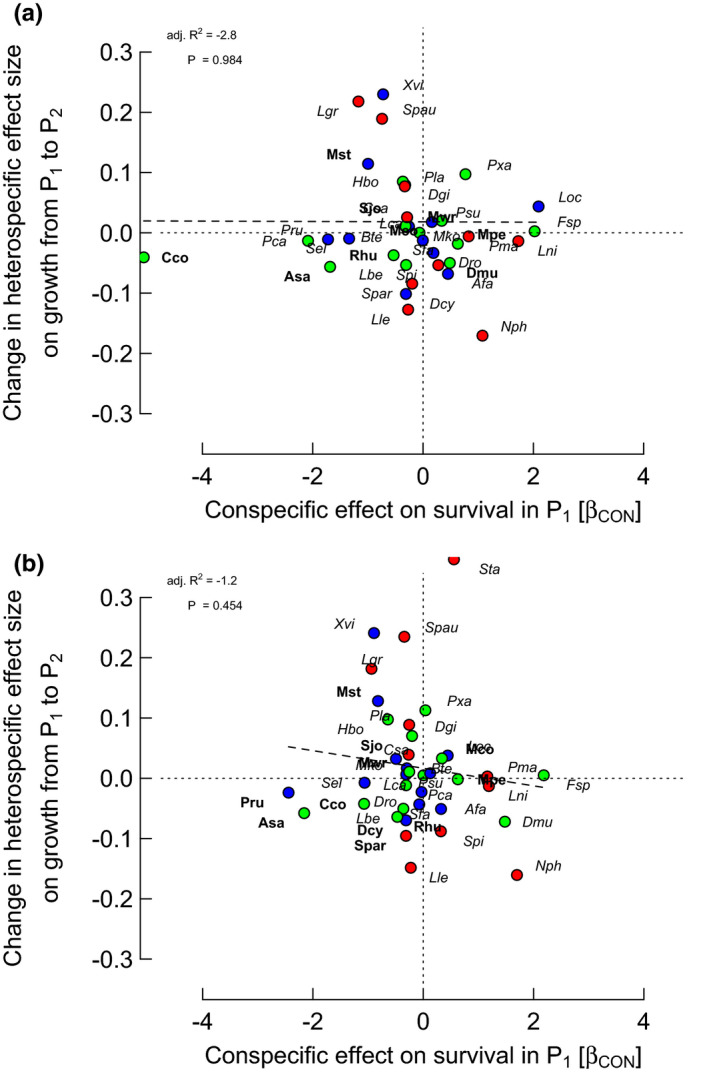
Relationships between *differences* in heterospecific (HET) effect sizes on growth rates between periods (P_2_ − P_1_) and CON effects on survival in period 1 (P_1_; *β*_CON_ = change in log[ODDS] per unit change in log(1 + sum(CON_ba_))) for the 38 species, from spatially extended neighborhood models with size (ba) and two neighbor terms (HET and CON). Points of focal trees having points of bigger neighbors within their zone of influence were either (a) removed or (b) relocated. Details are the same as for Figure 2

By contrast to the difference in CON growth effects, the HET difference effect on growth P_2_–P_1_ and CON, or HET, effect on survival in P_1_ for “larsm/reloc/crown” or “larsm/remov/crown” were all insignificantly correlated with the range in *r* = −.136 to −.003 (*p* = .42 to .98) (Figures 4 and 5). However, this HET difference effect on growth P_2_–P_1_ was much better—yet in opposite ways—correlated with CON or HET effect on survival in P_2_ for “larsm/reloc/crown” with *r* = .367 and −.516, respectively (*p* = .023 and .001), and for “larsm/remov/crown” with *r* = −.061 and −.602, respectively (*p* = .72 and <.001). Differences in HET growth effects P_2_–P_1_ on survival as the sums of CON and HET effects on survival in P_2_ were also significantly negatively correlated for P_2_ (*r* = −.541 and −.331, *p* < .001 and .043, respectively, for “larsm/remov/crown” and “larsm/reloc/crown”), but not P_1_ (*r* = −.039 and −.160, *p* = .82 and .34).

Community‐level graphs using nonspatial models that complemented the two spatial ones in Figures 2, 3, 4, 5 showed no trends or significance for a dependence on effect on survival (CON or HET) in P_1_ (Appendix S1: Figures S3 and S4). Further, community‐level graphs that used simply CON effect on growth versus CON effect on survival within one period, P_1_ or P_2_, also showed no significant relationships (Appendix S1: Figure S5). The interesting trends happen, therefore, when *difference* in growth effect between P_1_ and P_2_ is related to effect on survival in P_1_ (CON and HET) using a *spatial* model.

Within the best‐fitting spatial model, “larsm/reloc/crown,” CON effect on growth in P_1_ was significantly and positively correlated with that in P_2_, and also positively with HET effect on growth in P_1_ (*p* < .001) yet less strongly with HET effect on growth in P2 (*p* < .10) (Appendix S1: Table S5). Likewise, CON effect on growth in P_2_ correlated positively with HET effect on growth in P_1_ and P_2_ (*p* < .05), but HET effects on growth in P_1_ and P_2_ were much less strongly correlated (Appendix S1: Table S6). CON and HET effects on survival in P_1_ and P_2_ were all generally poorly and insignificantly correlated with one another, except for CON effects on survival in P_1_ with HET effects on survival in P_1_ (positive). Stronger was the correlation between HET effect on survival with the same on growth in P_2_ (*p* < .01). Between survival and growth variables, few were significantly correlated apart from CON survival in P_1_ with HET effect on growth in P_2_ (positive) and the same with HET effect on survival in P_1_ (negative). The model “larsm/remov/crown” had a similar pattern of correlations with an even stronger negative correlation for HET survival and growth in P_2_ again (Appendix S1: Table S6).

The relationship between CON difference effect on growth P_2_–P_1_ and CON effect on survival in P_1_ taken as a linear regression, that is, assuming now a dependence of growth effects on survival effect, indicated that the best‐fitting community‐level plots were for “larsm/reloc/stem” and “larsm/reloc/crown” (*p* < .05), and that removing overlapping canopy instead of relocating it led to much lower fits (*p* > .15) (Table 6a). Having “equal” instead of “larsm” canopy allocations led to also weak, marginally significant fits. Restricting the regressions though to those 24 species which had CON effects on survival significant at *p* < .1 (no‐decay mode) led to much stronger fits than for 38 species, with adjusted and predicted *R* ^2^‐values up to almost 30 and 16%, respectively, maximal for the “larsm/reloc/stem” and “…/crown” spatial forms (Table 6b).

**TABLE 6:** Dependence of the difference in conspecific (CON) effect sizes in growth rates between periods (P_2_–P_1_), for the different nonspatial and spatial models using linear distance decay, on the CON effects on survival in period 1(P_1_), for (a) all 38 species, and (b) the 24 species for which CON effects on survival in P_1_ were significant at *p* < .1 (see text for details)

Model	ppsqmca	Cover	Position	Periods 1 and 2		
				Adj. *R* ^2^	*p*	Pred. *R* ^2^
(a) 38 species						
ba + ALL	–	–	–	7.7	.051	−0.2
ba + HET + CON	–	–	–	−0.5	.369	−21.4
	Equal	Removed	Stem	2.1	.190	−5.5
			Crown	2.2	.182	−5.9
		Relocated	Stem	6.4	.068	−3.4
			Crown	6.1	.073	−3.6
	Larger > smaller	Removed	Stem	0.2	.305	−7.1
			Crown	1.6	.215	−5.7
		Relocated	Stem	11.8	.020	2.0
			Crown	10.6	.026	0.7
(b) 24 species						
ba + ALL	–	–	–	1.5	.260	−22.3
ba + CON + HET	–	–	–	−1.5	.422	−31.3
	Equal	Removed	Stem	10.5	.067	1.3
			Crown	7.1	.111	−30.1
		Relocated	Stem	15.7	.031	3.1
			Crown	15.7	.032	2.7
	Larger > smaller	Removed	Stem	3.9	.178	−9.3
			Crown	6.8	.116	−8.0
		Relocated	Stem	26.0	.006	12.9
			Crown	25.9	.006	12.8

Note

The models involved basal area (ba) and one or both neighborhood terms (CON + HET) and, in the spatial case, one of the eight different forms of crown extension (as structured in Table 2). The final effects sizes came from model averaging (as in Appendix S1: Table S2).

In the community‐level graphs, neither over‐understory status nor spatial patterning of trees (could explain differences and trends in the CON and HET effect (Appendix S6 for detailed results: Table S1 and Figure S1). However, in P_1_ though not P_2_, both CON and HET effects were significantly negatively correlated with stem relative growth, recruitment and mortality rates from early plot census analyses, that is, species with strong negative effect values had also fast growth and population dynamics (Appendix S6 for detailed results: Table S2.).

A case could be made for excluding *N. philippinensis* and *A. sanguinolenta* (two of the five species whose changes in effects with radius were unusual) from Figure 2b but that would have moved the fitted line only very slightly upwards with a similar slope (the points become a little more positive for CON effect on survival in P_1_). In conclusion, the final set of 38 species’ values appear quite robust for the community‐level analysis. At this community level, correlation or regression of species’ slopes versus CON or HET coefficient was not feasible due to high skew and strong leptokurtosis, respectively.

### 3.9 Spatial and nonspatial models compared

The community‐level graphs of difference in CON effect on growth P_2_–P_1_ versus either CON or HET effect on survival, particularly for the “larsm/crown/reloc” model (Figures 2b and 3b), showed stronger and more significant relationships than those with nonspatial models (Appendix S1: Figure S3a,b). Correlation between CON effects on growth in P_1_ and in P_2_ between the two models was both strong (*r* = .802 and .848, respectively, *p* ≤ .001), but for CON and HET effects on survival in P_1_ they were weaker (*r* = .570 and .567, *p* ≤ .001). These differences between the two community‐level plots are more the likely due to these CON and HET effects on survival.

To come closer to understanding the reasons for these differences, graphs of CON effects on survival in nonspatial and spatial models, and the same for HET effects, showed that the nonspatial model was more prone to serious outliers away from the general linear trends than the spatial one (Appendix S1: Figure S6). Indeed, for CON effects one species, *Polyalthia rumphii*, had an extremely large negative value, and for the HET ones three species, *P. rumphii*, *P. sumatrana,* and *Syzygium tawaense*, had unusually high positive values. Clearly these points created considerable leverage and were the main causes for the lack of agreement in the spatial and nonspatial community‐level graphs. The probabilities associated with the *t‐*values for these effect sizes coefficients in the individual species’ regressions were all large (0.41; 0.42, 0.88, and 0.98, respectively), that is, insignificant, indicating large uncertainties about the estimates. Otherwise, high *p*‐values were mostly attached to the coefficients close or at zero in Figures 2b and 3b, that is not distinguishing them much from null effects. *Cleistanthus contractus* and *Ardisia sanguinolenta*, both moderately separated from the main cluster of points in Appendix S1: Figure S4a had effect sizes that were significant or marginally so (*p* = .02 and .09). These subtle differences in reliability are not quite so apparent from the fonts applied to species codes in Figure S4. In passing, the remarkable species *S. johorensis* had outlying positive values for its difference in CON effect on growth P_2_–P_1_, for both the nonspatial and spatial models, but that was accounted for by the highly significant large effects in P_1_ (*p* ≤ .001) despite the corresponding effects in P_2_ being very close to zero (*p* > .05).

Omitting the above three outlying species resulted in stronger correlations between difference in CON effects on growth P_2_–P_1_ and CON effect on survival in P_1_ for the nonspatial (*r *= −.313, *p* = .067) and spatial (*r *= −.366, *p* = .031) models. For HET effect on survival in P_1,_ the improvement was correspondingly even better (*r* = −.430 and −.364, *p* = .010 and .031). The main reason, however, that the spatial models were slightly superior overall than the nonspatial ones despite the more rigorous selection of species’ estimates was that a group of five species, *D. muricatus*, *F. splendidissima*, *Lithocarpus niewenhuisii*, *Mallotus penangensis,* and *Neoscortechinia philippinensis*, became more spread in their increasingly positive CON effects on survival in P_1_. A similar case for positive HET effects on survival in P_1_ is less strong though, involving just *L*. *niewenhuisii, N. philippinensis*, and *Lithocarpus gracilis*. Compared with nonspatial models, spatial models seemed to emphasize more positive rather than negative effects on survival. The two models, nonspatial and spatial, became more similar with the 35‐species analyses.

Taking the axes coordinates for the spatial and nonspatial graphs of difference in CON effect on growth P_2_–P_1_ versus CON effect on survival in P_1_ as two 35 × 2 matrices, Procrustes rotation (package vegan in R; Mardia et al., 1979, Oksanen et al., 2019) highlighted strong agreement between the models, rescaling the spatial matrix to the target nonspatial one gave a correlation coefficient of 0.423 (*p* = .011; tested with 999 randomizations). Repeating this procedure for HET effects on survival in P_1_ led to a Procrustes correlation of 0.863 (*p* = .001). Thus, comparison of HET‐based community graphs led to better model matching than did CON‐based ones.

### 3.10 Randomization of neighborhoods

Across the 48 species, randomized mean CON effects on growth in P_1_ and P_2_, and their differences P_2_–P_1_, as well as CON effects on survival in P_1_ and P_2_, were mostly not significantly different from zero, judged by their confidence limits calculated as ±3 *SE* (Appendix S7: Figure S1). Cases of significance occurred more often among the 10 excluded species, and particularly for survival effects: In these cases, the limits were usually much larger than for the 38 retained species (see Appendix S1: Table S1). Retrospectively, the species selection was therefore well supported.

Considering only the 38 selected species, the mean observed effects were significantly different from the randomizations (i.e., they lay outside of the ±3 *SE* limits) either positively, not or negatively for 5, 7, and 26; 8, 11, and 19; and 17, 11, and 10, of them for CON effects on growth in P_1_, P_2_ and P_2_–P_1,_ respectively. The corresponding numbers for CON effects on survival in P_1_ and P_2_ were 8, 10 and 20; and 8, 19 and 11 (Appendix S7: Figure S1). These frequencies show clearly that CON effects on growth in P_1_ and P_2_ were negative for a majority of species but differences moved to being mostly positive. Likewise, CON survival effects in P_1_ were in the majority negative too, but in P_2_ more species’ effects were insignificant, and positive and negative effects were more similar in frequency. Numbers of differences (positive, negative or null) among the other 10 species are uninformative given the statistical grounds for these species’ exclusion.

Defining moderate limits as being 0.06 to <0.1 and 0.6 to <1.0 for growth and survival effects, respectively, and corresponding large as being ≥0.1 and ≥1.0, among the 38 selected in P_1_ and P_2_ species such moderate and large limits for growth effects were moderately frequent (28 of 2 × 38 combinations, 37%). For the difference in CON effects, P_2_–P_1_, 18/38 species (47%) had medium and large differences, while for survival ones they were similar (24/76, 32%). For the excluded 10 species, a majority of limits, for both growth and survival, were moderate or large (27 of 4 × 10, 67.5%).

HET effects on growth in P_1_ and P_2_, and their difference P_2_–P_1,_ plus HET effects on survival in P_1_ and P_2_, were mostly not significantly different from zero, judged by their confidence limits calculated as ±3 *SE* (Appendix S7: Figure S2). As with the CON effects, limits (±3 *SE*) were much larger for the 10 excluded than the 38 selected species.

Of the 38 species, mean observed HET effects differed significantly from the randomization means positively, not or negatively for 4, 8 and 26; 2, 8 and 28; and 14, 9 and 15 of them for growth in P_1_, P_2_ and P_2_–P_1,_ respectively. Thus, HET effects on growth were again predominantly negative in both periods. The numbers for survival in P_1_ and P_2_ were correspondingly 13, 10 and 15, and 18, 9 and 11. Hence for growth ± or 0 cases were rather similarly distributed over the 38 species for HET as for CON.

The strengths of the HET differences between empirical and randomized means, calculated in the same way as for CON effects, were very similar to the latter: 28/76 (37%) of species with medium and large differences in P_1_ and P_2_, 14/38 (37%) for P_2_–P_1_. For survival, medium and large HET effects differences in P_1_ and P_2_ formed 22/76 cases (29%). Among the excluded species, these differences were also frequent with 20/40 (50%).

Putting CON and HET effect differences in comparison, significant negative CON and HET effects were in the majority for growth in P_1_ and P_2_; and with CON a majority positive, but HET more evenly distributed, for P_2_–P_1_. For survival in P_1_ and P_2_, CON effect differences were also predominantly negative in P_1_, yet fairly evenly positive or negative in P_2_ with most nonsignificant. HET effect differences showed the converse however, being evenly negative, not and positive in P_1_ yet predominantly positive in P_2_. For many species, there was evidently a shift in sign of survival differences between P_1_ and P_2_. Due in part to their sampling unreliability, differences for the excluded 10 species were often more pronounced for CON than HET (Appendix S7: Figure S2 cf. S1).

Simulating the regression of difference in CON effects on growth P_2_–P_1_ versus CON effects on survival in P_1_ 100 times using the randomizations, for all 38 selected species, and “larsm/reloc/crown” spatial model, resulted in 62 slopes that were positive and 38 that were negative. Seven lines were individually significant at *p* < .05, two with negative and five with positive slopes (Appendix S7: Figure S3a). Just one *t*‐value (of −3.2) for the slopes was more negative in the randomizations than in the observed relationship (*t* = −2.3). This supports the inference (on the basis of a two‐tailed null hypothesis) that the relationship in Figure 2b is statistically robust at *p* < .05 level (arguably at *p* ≤ .02) since it lies outside of the 95% (or 98%) confidence envelopes of *t*‐values under the null hypothesis of randomly positioned neighborhoods. These randomizations will have removed any influences of spatial aggregation and therefore local dominance of species.

Repeating the community‐level simulation with just the 24 species with significant CON specific effects on survival in P_1_ resulted in 59 slopes that were positive and 41 that were negative. Seven lines were individually significant at *p* < .05, two with negative and five with positive slopes (Appendix S7: Figure S3b). Neither of the negative *t*‐values of the slopes (both − 2.2) was more negative in the randomizations than in the observed relationship (*t* = −3.0), supporting the inference that the relationship was statistically robust here at *p* < .01.

The proportion of slopes of the difference in CON effects on growth P_2_–P_1_ versus HET effects on survival in P_1_ from the 100 randomizations were 46 negative and 54% positive, with 12 simulation lines significant (*p* ≤ .05), five negative and seven positive (Appendix S7: Figure S3c). Four *t*‐values for slopes (−2.8 to −3.3) were more negative in the randomization than in the observed relationship (*t* = −2.3), indicating only significance at *p* < .1 on a two‐tailed basis. Why there was an imbalance of positive to negative slopes for CON (~60:40) compared HET (~50:50) under randomization remains to be explored.

Again, considering the 24‐species community‐level HET relationship, 50 and 50 of the slopes were, respectively, negative and positive and 14 lines were individually significant (*p* ≤ .05), seven negative and seven positive (Appendix S7: Figure S3d). The empirical regression, accounting for very similar variance as for the relationship with 38 species, had (the) seven randomized slopes all more negative than that for the observed relationship (*t* = −1.8), suggesting that H_0_ might be rejected only at *p* ≤ .2. Narrowing the species considered from 38 down to 24 had an opposite effect for HET than it did for CON relationships in terms of improved statistical fitting (worsening versus improving, respectively). Overall, the strength of the HET relationship was therefore much less significant (indeed nonsignificant at *p* > .1) than that for CON (significant at 0.02 ≤ *p* < .05) based on the randomization testing.

Using the mean effects from the randomizations, difference in CON effects on growth P_2_–P_1_ versus CON effect on survival in P_1_, for the 38 species—plotted in a similar way as for empirical data in Figure 2b, had a positive correlation (*r* = .360, *p* = .026). However, excluding one clear outlier (*Hydnocarpus borneensis*), the correlation was then closer to zero (*r* = .080, *p* = .640), as should be expected from an effective randomization procedure. Had the number of randomizations been higher than 100, this and maybe other outliers would have been less important.

## 4 DISCUSSION

### 4.1 Spatial and nonspatial models

Difference in CON effect on growth P_2_–P_1_ was significantly *negatively* correlated with both CON and HET effect on survival in P_1_, but not in P_2_. Conversely, the difference in HET effect on growth P_2_–P_1_ was correlated *positively* with CON, yet negatively with HET, survival in P_2_—but not in P_1_. There was therefore a part reversal of CON and HET effect associations over time. CON and HET effects on growth were positively correlated with one another in both P_1_ and P_2:_ CON and HET effects on survival, however, were positively correlated only in P_1_. Hence, CON and HET effects on growth, and on survival, appear to have been coupled in P_1,_ and then decoupled in P_2_. The randomization tests showed a high statistical confidence in the relationship between difference in CON effects on growth and CON effect on survival (*p* ~ .02), but a similar relationship for HET effects was not nearly so robust (*p* > .1). While HET effects evidently had a role in the tree neighbor interactions in P_1_ and P_2_, the main results concern the CON effects: Conspecific interactions might be seen as being embedded in a diffuse matrix of heterospecific ones. While the “larsm/reloc/crown” spatial model was marginally the best supported realization of zone of influence competition, the concept and alternative models may have interpretational difficulties, highlighting the almost intractable complicatedness of diverse forest tree‐tree interactions.

The models used in this paper defined neighbors as trees lying with a radius of 20 m and with gbh greater or equal to that of the focal one. For the majority of understory species, this meant that their focal trees often had few conspecific neighbors, especially if the focal trees themselves were ~50–100 cm gbh, because these species rarely ever attained sizes of >50 (or even 100) cm gbh. The opposite was the case though for overstory species; for them conspecific neighbors were often far larger, and sometimes included the largest trees in the plots. On a simple biomass basis, then, CON effects on understory species were expected to be rare and not as strong as the commoner overstory ones, although both would be subject to similar levels of HET neighbor basal area. The effects of large‐canopy conspecific trees would be even higher when the adults were aggregated (Newbery & Stoll, 2013). In addition, removal and relocation of parts of crowns in spatial models was affecting mostly sub‐canopy overstory trees, those that on the one hand were being overlapped by upper canopy and emergent trees’ crowns, and on the other hand were remaining still large enough to make major contributions to CON and HET basal areas. Zone of influence adjustments, according to story position and size‐class (gbh) frequency distribution, determined differences in how spatial and nonspatial models were operating for each species. The influence of adjustments on regression fits was weaker for under‐ than overstory species. But then, most understory species would not be expected to be plastic in their crown adjustment to move toward light, because they are shade‐tolerant trees, and drought‐tolerant ones would only be temporarily exposed to higher light levels to have had insufficient time to change crown position before the canopy closed again.

For a neighborhood model involving above‐ground architectural traits, allometric regressions of crown area versus gbh would ideally have been better constructed for each of the 38 tree species and had sufficient data been available. Combining all species led to an averaging of crown areas across different species in each gbh class, for example, a small tree of a dipterocarp (overstory) and one of a euphorbiaceous (understory) species would have been equivalent. Height was not involved in the crown overlap (removal/relocation) calculations: It was tacitly assumed that a tree with a large gbh was always higher than, and overlapping, a small adjacent one. This had important consequences for the crown adjustment algorithm. Crown depth and volume were not involved either. The simple nonspecies‐specific allometric approach may, therefore, have distorted the true variation within and between species. The basic model, while being in some ways more realistic in the incorporation of crown area at all, may have introduced complicated biases when these crowns did not match well with each species’ ecological‐defined height‐diameter‐crown area relationships. Spatial models adjusted crowns by removal and relocation of parts of them when overlap occurred. If this really was happening in the forest, Sterck et al. (2001) would have incorporated them when making their crown measurements. So to some extent, natural crown adjustment was already in the allometric equation. In this connection, the influence of coordinates of focal tree stem versus those of crown centroid was barely detectable in the outcomes of the spatial models.

The algorithms used for adjusting crown (and root system) overlap came nevertheless at a cost to some realism of the spatial models. Nonspatial models, with basal area at a distance from the focal tree placed at a neighbor tree's center, and the spatial models with Δ*d* = 0 where crown and root system extents were determined (without any adjustments) by the common allometric equation, present two well‐defined ends of a scale in crown extension, none to full. However, once Δ*d*_CON_ or Δ*d*_HET_ were allowed to increment positively, adjustment meant potential removal or relocation. When overlap of smaller trees however was complete, either by one larger crown, several together, or one causing relocation of a less large one in a domino‐manner, they could disappear as neighbors. This was likely to happen often because firstly the predicted LAI was close to 2.5 when Δ*d* was 0, and secondly, understory species’ trees from their ecologies are almost always shaded by others, especially in P_1_. The analysis of Δ*d* values selected by the best‐fitting models showed that in the main Δ*d* was not 0.0. Even though the C_2_ two‐term model was used, many understory species had reduced CON basal areas because when small and fully shaded from above, they were still larger in gbh than the majority of the focal trees. Shading hierarchy paralleled story structure and may have given an undue bias to CON effects of overstory species (as was the selection in Stoll & Newbery, 2005).

In building the nearest‐neighbor models, the focal tree was a point, with no crown extension or adjustment. Another, but computationally far more demanding approach, would have been to allow focal trees to have irregular crowns and then find CON and HET basal area for each of the allocated points, and integrate basal areas per tree. But then, focal trees themselves would be susceptible to disappearance if they were completely overlapped, presenting a dilemma. The random positioning of points in crowns at the start, before any adjustments for overlap, was also done just once. Had this step used multiple randomizations, then points allocation, adjustments, and crown shapes would have been allowed to vary and thereby provided more robust mean effect sizes. However, this second potential extension involves prohibitively long computation times. Catering for these two fine‐scale (within‐crown) sources of variability may not have affected the qualitative outcome of the analysis of the empirical data too much but it would have allowed for some modeling uncertainties to be taken into account. The many radial increments times Δ*d* levels led to large numbers of very similarly fitting models, particularly as points in crown area (ppsqmca) were from single crowns as neighbors and very small changes in the fitted coefficients came from the radial points moving across at 1‐m increments.

Using spatial extension posited that the statistical modeling would move a step closer to forest realism in that above‐ and below‐ground allocation of (neighbors) biomass under the symmetry/asymmetry of competition within the zone of influence would capture the CON and HET influences better than a nonspatial model. Neighborhood models for the individual species highlighted, though, that while involving spatial models did more often explain focal tree growth (not survival) better than nonspatial ones, those models with a ppsqmca allocation proportional to tree basal area (“larsm”), either with relocation or removal of overlapping parts of crowns, were not more often better than those with an even allocation (“equal”). The former introduced an asymmetry, or nonlinearity, in neighbor interactions, the latter not: However, increasing Δ*d* also introduced degrees of asymmetry so that as this parameter was increased, removal or relocation led to “equal” points distributions becoming less equal. Conversely, with the “larsm” model form a larger shading crown could cause a lower crown to be adjusted, which when through relocation would increase the lower one's ppsqmca.

Conspecific effects of neighbors on growth in P_1_ were more weakly (though still significantly) related to plot‐level BA for spatial than for nonspatial models, and likewise for differences in CON effect on growth P_2_–P_1_, but there was no influence of model form for CON effects versus BA in P_2_. Conspecific effects of neighbors on survival in P_1_ and P_2_ were also independent of BA, whether the model form was nonspatial or spatial. The relative strengths and directions of the relationships for CON effect on growth versus plot BA in P_1_ and P_2_, for nonspatial models, is the same as reported before (Newbery & Stoll, 2013; Stoll & Newbery, 2005). If spatial extension (in the form of plasticity of crown size and overlap, and location) was supposed to simulate competition for light between neighbors better, and be a basis for the proposed negative density dependence of CON effect size on plot‐level species abundance (i.e., more abundant species in the plots with higher BA had associated with them stronger more‐negative CON effects on their small trees), the reduced fitting would imply either that any driving causal influence of abundance per se (plot BA) was not happening through asymmetric competition for light, or that nonspatial models without plasticity overestimated the “true” effect of negative density dependence. Alternatively expressed, plasticity allowed crowns to be distributed closer to how they are thought to compete for light yet removed the conspecific negative density dependence (Stoll et al., 2002).

The reduced fits of the spatial compared with the nonspatial models can be explained best by the application of Jensen's Inequality (Jensen, 1906; Ruel & Ayres, 1999) (see also Ross, 2014), and most simply for Δ*d* = 0, because the 1/*d* weighting of basal area is a concave function of *d*. The mean of the inverses of distances from the focal tree location to points in the neighbors (circular) crown will always be greater than the inverse of the distance from the focal tree to the center of the neighbor one (being the mean coordinate of all points in that crown). Consider a neighbor of crown radius 1 m whose center is 3 m from a focal tree. The closest point on the circumference of the crown is 2 m, and the furthest point 4 m from the focus. The mean of their 1/*d* values is (0.5 + 0.25)/2 = 0.375, but 1/*d* for the center of the crown is 1/3 = 0.333. A more extreme example: A crown of 5 m radius has its center 7 m from the focal tree. The corresponding means of inverse distance and distance from center to focus are (0.5 + 0.083)/2 = 0.292 and 0.143. Thus if ppsqmca are allocated under “larsm” at random within crowns equal in number to the neighbor tree's basal area in cm^2^, a spatial model will always give a higher weighting to that neighbor's basal area compared with the nonspatial model with all basal area at the tree's center. It follows that the spatial models will fit less well than the nonspatial ones because the larger CON and HET basal areas are moved more positively, away from zero, on the *X*‐axis which leads to the dependence of growth or survival (the slope in the regression) to be less steep. Nevertheless, the model fits for “no,” “lin,” and “squ” distance weightings differed rather little (Appendix S1: Table S2) suggesting that the rescaling caused by the different weightings was, though important, small in its influence. The logarithmic transformation of CON and HET basal areas in the models would have dampened the differences between types of distance weighting.

The same arguments apply to the “equal” allocation of crown points, but the influence of the inequality will be generally less because ca is proportional to ba^1/2^ and not ba. Adjustments for crown overlap by removal or relocation emphasized the influence of Jensen's Inequality. Adjustment of points by removal would leave them on average both closer to or further away from a focal tree than before (i.e., to either side of a shading larger crown), or by relocation increase the average the inverse distance weighting further. Since logistic regressions are more sensitive to changes in range and skew in predictor continuous variable (CON and HET basal area weighted by 1/*d*) than Gaussian normal ones, the loss in fit moving from nonspatial to spatial models will be greater for CON effects in survival than CON effects on growth especially. For HET effects, the differences are ameliorated by the heterospecifics making up that large matrix of neighbor trees that shift about in their canopy positions between one another under adjustment much less than is experienced for conspecific trees.

By the same token, that the relationship between CON effects on growth in P_2_ versus BA fits was barely altered under spatial extension, and it was shown that CON effects were overall relaxed in this period compared with P_1_ (they became less negative, Newbery & Stoll, 2013), then another factor such as competition for, or utilization of, nutrients might account better for the negative density dependence—but only if patterns of nutrient acquisition are not following light ones in the same way, that is, the root systems are crown size and crown shape unrelated. That the slopes of relationships changed little between nonspatial and spatial modes, even a little less steep for the latter compared with the former suggests that the lowered variance accounted for was mainly due to added variability coming from the common crown allometric equation being unsuitable for all species, the random allocation of crown points, and way overlap led to crown or root system removal or relocation. This together raises then the possibility that below‐ground interactions were as or more important than above‐ground ones in this forest.

These aspects all likely contributed to the poorer fits of spatial models than the nonspatial one for CON and HET effects against plot BA. It is further notable, that the regressions against plot BA and the community‐level graphs showed no clear trends between overstory and understory species other than larger‐stemmed overstory species tending to be out on the extremes of the negative relationship and the understory ones clustered at the center. The spreading of a group of species to the other side (CON effects on survival in P_1_ being positive) is of considerable interest. Had light been the predominant factor a clearer story‐related pattern should have been more evident (see Newbery et al., 2011), especially in view that, because of the disappearance of smaller crowns, overstory species were emphasized over understory ones.

### 4.2 Changes between periods and conspecific mechanisms

The evidence and arguments so far suggest that competition for light was not the main or sole driving factor behind CON and HET effects. Asymmetry of competition can be explained physically in terms of light interception and shading, and yet the very strong asymmetry that the models invoked (Table 2) did not lead to significantly better model fits than with symmetry. By putting aside the outlying and statistically unreliable estimates of four of the species, the “larsm/crown/reloc” spatial model scaled well on to the nonspatial one. Competition among root systems is normally expected to be much more symmetric than among leaves and crowns (neighboring trees accessing and taking up resources in the soil in proportion to their respective biomasses), and with the involvement of ectomycorrhizas the interaction can be even more facilitative than competitive (Newman, 1983). However, in competition modeling, resources below ground (principally for nutrients outside of dry periods) have often been assumed to have either a negligible role or to be operating similarly, or in proportion, to the light factor. This may be more likely for fast growing, colonizing or secondary forest growth, but it is difficult to understand for a late end‐succession or mature primary forest like Danum (Newbery et al., 1992).

There exists an implicit assumption with the zone of influence competition modeling concept when defined by simple spatial extension of tree form and mass, its icon being crown area. Corresponding to the crowns, overlap and plasticity is directly and similarly implied for the root systems. The same effects of removal and relocation are sensu lato also implied for processes below ground, vertically matching crown with root system adjustments, so that presumably smaller trees roots are, respectively, thinned or concentrated away from those of larger ones. But is this spatial model, even in its main components, realistic? Based on the differing physiologies of the tree parts, only a broad correlation between root system and stem/crown biomasses across tree sizes would be expected. The idea of “overlap” remains importantly problematic. For light competition it is readily interpretable, but for root systems a tendency toward a symmetry or linearity of full intermixing in acquiring water and nutrients would be expected. The overlap Δ*d*‐value (up to 1.2 m) was to allow for an oblique shading zone; but for roots the equivalent is unclear—a depletion zone for water and nutrients that did not have daily or annual variation? The parameters Δ*d*_CON_ and Δ*d*_HET_ were set to apply in the same way for all species, irrespective of their leaf size and density, branch structure, root distribution, and corresponding ecophysiological differences.

The growth rate calculations in this paper did not specifically exclude all trees with “invalid” measurements (see Lingenfelder & Newbery, 2009), that is those where the point of measurement had moved slightly, stem (bark) condition changed or deteriorated, recording was inaccurate due to liana growth, etc. At the 1996 and 2001 censuses (end of P_1_ and P_2a_) close to 10% of growth estimates were invalid, and trees with invalid estimates had rates on average 47% lower than valid ones (Newbery & Lingenfelder, 2009). However, excluding, in this paper, trees with >−3 *SD* deviations on the species’ agr‐versus‐log(ba) regressions will have caught most of those more extreme low‐growth values. As a rough estimate, then, reliable growth rates were likely up to 4.7% underestimated, and the assumption has to be made for the present modeling that this small bias applied fairly evenly across periods and species and had little influence on the conclusions. The dependence of growth rates on the field‐recorded gbhs at two times is sensitive and prone to error and bias: And, this latter is more acute the more trees in the population are dying or close to death (with prior declining rgrs). However, mortality rates (trees ≥ 10 cm gbh) were 27% higher in P_2_ than in P_1_—1.99% versus 1.57%/year, so “true” rates would have been underestimated a little more so in P_2_ than P_1_, and thus, the difference in CON effect on growth P_2_–P_1_ for species with the relatively higher within‐period mortalities in P_1_ and P_2_ underestimated. The slopes of the lines in the community‐level graphs of Figure 2 are therefore slight underestimates. This issue of growth rate validity is crucial to evaluating how mortality depends on prior growth rate because assessing growth rate is very difficult when the stem itself is deteriorating just before death (Lingenfelder & Newbery, 2009).

Compared with P_1_, the temporary decrease in soil water availability followed by increases in light levels to the understory caused by the 1998 ENSO event in P_2_, differentially affected changes in species’ growth and survival rates between periods (Newbery & Lingenfelder, 2004, 2009; Newbery et al., 2011). However, the extent to which differences in response between individuals of any one species were directly caused by the drought/light‐change environment or were indirectly caused by their also‐affected neighbors’ growth and survival rates, or both, depends on a highly complex set of spatial–temporal tree–tree interactions. Integrating across each species’ tree population results is simply their average growth and survival rates for each period. A species responding positively to the P_1_–P_2_ external change might be expected to become more competitive for both above‐ and below‐ground resources and to thereby have stronger CON‐HET effects on its neighbors. Conversely, a negative response could be because the neighbors are responding more negatively to the change and their CON‐HET effects become weakened.

The community‐level diagrams indicate that those species which suffered the largest CON and HET effects on survival from neighbors in P_1_ (i.e., their relative increase in mortality from this cause was highest) had the largest releases from CON—but not HET—effects on growth P_2_–P_1_. Conversely, those species that were little, or even positively, affected in their survival by CON and HET neighbors had either small releases or decreased negative effects on growth between P_1_ and P_2_. Expressed otherwise, with growth rates intermediate or even slightly higher in P_2_ than P_1_, yet more equally spread across species in P_2_, the more the suppressed species in P_1_ appeared to be released and the less suppressed ones hampered. The neighborhood regression models only test though for CON and HET effects on growth rates within periods (separately); but they do not test for an interaction between CON and HET effects on growth rates and periods, which for the inferences drawn for this paper was assumed to be zero.

One potential explanation for the patterns in the community‐level graphs is that local thinning of focal trees in P_1_, due to the CON effects on growth and survival, might have relaxed competition between them in P_2_ and thus created the release in CON effect on growth. If that were to have operated, the relatively small focal trees would have had to be very close to one another indeed to allow for intraspecific interactions to operate (mechanistically). This was generally not the case. Just one species, *D. muricatus*, possibly reached sufficient local densities in clusters on ridges (Newbery et al., 1999; Newbery & Ridsdale, 2016). This species was most unremarkable on account of its position on the community‐level graphs. A HET effect on survival in P_1_ however seems more plausible on spacing grounds, and so together it can be supposed that CON and HET basal area together contributed to any general density or thinning effect (Figures 2 and 3).

The physiological process by which prior slowed growth leads to the death of a tree, and how growth and mortality are actually recorded in populations over time, is fundamental to an understanding and interpretation of the community‐level relationship in Figures 2 and 3. For the Danum forest this was demonstrated by Lingenfelder and Newbery (2009) and Newbery and Lingenfelder (2009). If probability of tree mortality is generally continuously related to stem growth rate, that is a population is not divided into say two discrete classes where one is dying very fast independently of growth rate and the other very slowly (as in, perhaps, an age‐related disease susceptibility situation), species with higher mortality rates will be expected to have on average (prior to death, and for those remaining and not yet dead) lower rgr than do species with lower mortality rates. It is important to note that the CON effects on survival in P_1_ are not alone determining mean survival rate of that species, but only how CON basal area increases or decreases it. The consequences of the CON effect for species with a very low compared with a moderate or high average rate of survival may be different.

If the main effect of declining growth on reduced survival can be translated to negative CON effect reducing growth and negative CON effect reducing survival—since growth rate and survival overall for a tree are in part determined by the neighbor effects, then a CON effect that reduces growth rate further should also have the consequence of reducing survivorship further. Hence, a “release” in CON effect on growth P_2_–P_1_ will be larger (positive) for species with larger (negative) CON and HET effects on survival in P_1_ than those with smaller (positive and negative) effects on survival, because the more suppressed a tree is in its growth the greater the potential for release when conditions that caused the suppression are removed. That CON and HET effects on survival appear to be operating more in P_1_ than P_2_ is compatible with the thesis that small focus‐sized trees in the undisturbed, closed and shady understory will have lowered survival rates due to these relatively low light conditions, and their large competitors in the overstory will exacerbate the situation by exerting increasingly larger negative effects on their growth.

The processes that resulted in the *release* of CON effects on growth in P_2_ may not have been the same one either that was linking CON effects on growth to CON (and HET) effects on survival in P_1_. The one in P_2_ was being largely driven by an external change in the environment leading to increased and variable light conditions within the understory, whereas the one before in P_1_ was determined more by a steady environment with more closed, shaded internal‐forest conditions. Thus under a trade‐off in responses, shade‐intolerant species would be dying most in P_1_ and yet responding (i.e., the survivors and new recruits) most to light in P_2_, while shade‐tolerant ones would be less affected in P_1_ but, being weaker competitors under more lighted conditions they would continue to have lowered growth rates, CON effects on growth became minimal. The fact that differences in CON but not HET effects on growth were strong indicates that species‐specific root processes might have been interacting positively with a growth‐survival trade‐off along the light gradient, and this thereby offers a route to explaining species’ idiosyncrasies at the community level. There would be the freedom of various individualistic conspecific processes to be operating. The idea here is that it is not simply the difference in response to light that likely differentiated species (as shown in Newbery et al., 2011), but the interactive effect of CON neighbor roots on that overall tree growth response to light.

Could the results be the outcome of random patterns and processes operating? The lines fitted on the community graphs go through zero with a negative slope. A completely random set of responses would settle around zero according to the central limit theorem, and this was supported by the randomization runs: on the other hand, a net outcome of interactions between neighbors would also result if species were balancing out their negative and positive effects, particularly when most of the neighborhood of any one species is largely HET. A form of zero‐sum game in the whole forest might be implied. It may be axiomatic that all interactions even out in a dynamic equilibrium: the CON and HET effects that some species on average experience as negative, others experience as being positive. What characterizes the differences in CON and HET effects on growth and CON and HET effects on survival in the two periods might be largely a question of chance where the individuals happen to be located with respect to their neighborhoods. In mixed forest, areas will differ in local BA density either at random or with some degree of local clustering. High‐BA neighborhoods create greater overlap of zones of influence and competition than low‐BA ones. If a shade‐intolerant species should by chance happen on average to have more of its small trees in high BA patches, it would be expected to show a strong CON and HET effect on growth and survival; and if in low patches, weaker effects.

Three aspects suggest that species responses were highly idiosyncratic because of the lack of any interpretative trends. Firstly, the arrangement of species in the community‐level graphs in Figures 2 and 3 seem to have no clear explanation in terms of the under‐ versus overstory classification, or population variables for P_1_ and P_2_ (indicating a growth‐survival trade‐off across species), or species’ reactivities to the drying event in P_2_, which were all useful in explaining changes in tree growth rates between P_1_ and P_2_ (Newbery et al., 1999, 2011; Newbery & Lingenfelder, 2004). Further, there are no apparently excepting reasons for the outlying species’ points in Figures 2 and 3, even for *Shorea johorensis* with its very pronounced relaxation of the negative CON effect on growth between P_1_ and P_2_. No common consistent explanation can be offered either for the group of five species whose CON effect of survival was positive (lower right portion of Figure 2b).

Secondly, individuals of shade‐intolerant tree species are expected to respond more negatively, in terms of their growth and survival, than those of shade‐tolerant species as CON and HET basal areas increasing in their neighborhood impose more shaded conditions on small trees. When the limiting (light) condition is removed, shade‐intolerant species will respond faster than shade‐tolerant ones—the link between mortality on growth being relaxed. However, the simple categorization into shade‐tolerant and light‐demanding species (e.g., Turner, 2001; Whitmore, 1984) scarcely applies here with so few forest gaps and lack of secondary species. Several main understory species respond to increased light and are apparently more drought‐ than shade‐tolerant, and understory trees of overstory species show a wide variation in responses to changing light conditions (Newbery et al., 1999, 2011; Newbery & Lingenfelder, 2004, 2009, 2017). Therefore, to refer to any “trade‐off” connected with life‐history strategy is irrelevant and misleading for this forest. Although species may differ on average in their responses, a wide variation within species exists because of many other factors which interact with light, particularly nutrient and water availability.

Thirdly, the new evidence, using spatially extended models, does not dispel that CON effects were acting “by default” (Newbery & Stoll, 2013), that is, they arose when conspecific species were clustered and large trees formed most of the neighbors of focal ones locally. The spatial pattern analyses in the present study concluded that degree of aggregation as a general factor for all species was unrelated to the trends in the community‐level graphs. This does not mean though that it was not *one* of a set of factors leading to CON effects on growth and applied mainly to the large strongly clustered dipterocarps (Stoll & Newbery, 2005). Randomization tests will not only have removed clustering but all possible linkages between, and complementation of, neighboring root systems.

Since strong CON effects on growth contribute to lowered tree rgr, which when very low or at zero will usually result in tree death, the community‐level graphs in Figures 2 and 3 are representing a form of difference in CON effect on growth P_2_–P_1_ versus CON effect of growth in P_1_. And if CON (and HET) effects on growth (not growth rates per se) were randomly occurring in tree populations labeled as species, then negative slopes could be explained by the “regression‐to‐the mean” phenomenon (Kelly & Price, 2005; Tversky & Kahneman, 1974). Random changes in CON effects between P_1_ and P_2_ would mean that groups with high CON effects in P_1_ would have lower values on average in P_2_, and their difference becomes closer to zero, and those with low CON effects in P_1_ work in the opposite way but also moving toward zero (the overall “mean”). The correlation between CON effect on growth in P_1_ with that in P_2_, for the nonspatial and spatial (“larsm/crown/reloc”) models were 0.466 (*p* = .003) and 0.521 (*p* ≤ .001), respectively. So while the correlation was higher for the spatial model there was sufficient variation to cause a regression to the mean. That might *in part* explain the passing through the origin and the negative slope. Randomizations—which simply relocated tree positions—would be expected to show a similar trend, and they do, although the empirical line being just significant, suggests that a set of some real CON effects—perhaps just for the main large dipterocarps (Stoll & Newbery, 2005)—were operating in addition to any resampling‐over‐time artifact.

We hypothesize that the negative slope in the community‐level graphs of Figures 2 and 3 is in part determined by a gradient of strong‐to‐weak below‐ground rooting processes allowed by different degrees of response to light‐level change. The negative density dependence relationship of differentiating CON effects on growth depending on plot BA might then be better explained by below‐ground rather than above‐ground processes? Conspecificity, if it is real and not by “default,” must have mechanisms that are species‐specific in order to operate: adults must be affecting juveniles of their own more than other species, and juveniles are only, or very largely, affected by their own, and not all, adults. Although not applying to all large‐treed species, juveniles with ECMs may have been relieved in P_1_ of their dependence on close‐by adults for carbon and nutrient transfer under the P_1_ light‐limited conditions. With more light in P_2_, the focal trees became more autonomous as their own C input increased and this meant they could, though increases in fine root and hyphal growth, acquire phosphorus and other elements by uptake and transfer more independently.

The analyses of the neighborhood effects of large trees on small focal ones at Danum, over the two periods, presented a paradox. The negative density dependence relationship based on the nonspatial model was weakened in spatial models due to averaging of inverse distance weightings. If the latter with crown extension and plasticity is a truer representation of forest structure and tree‐tree interactions than the former without it, then the strength of negative density dependence was overestimated by Stoll and Newbery (2005). Yet the spatial models came with several provisos and limitations that questioned their realism, in particular in the ways the conspecific tree interactions for understory species were de‐emphasized and those for overstory species were over‐emphasized.

Nevertheless, the community‐level graphs relating differences in CON effects on growth P_2_–P_1_ to those on survival in P_1_ were slightly better for spatial than nonspatial models which suggested that the link between growth and survival responses to neighbors, and the change in the former under differing forest conditions (P_1_ to P_2_), was in part due to a dependence of survival on growth but also partly due to differences in relative importance of asymmetric and symmetric competition (potentially with facilitation via ectomycorrhizas). However, species of the Dipterocarpaceae and Fagaceae (likely also ECM) show no grouping on the community‐level diagrams in Figures 2 and 3. To achieve some progress on understanding the mechanisms behind the tree‐tree interactions, it is essential to have detailed data on root distribution, growth, and activity of each of the different species, and how allocation and plasticity in stems and crowns is related to those of root systems. For practical reasons this, however, is going to be extremely difficult to achieve with the limited sampling techniques currently available. For now, the only rare available data for Danum are overall fine root biomass and dynamics estimates, undifferentiated at the species level (Green et al., 2005). Appealing to similar studies in other forests for support (and extremely few exist) is unlikely to be useful as the interpretation of forest dynamics at Danum is closely dependent on precise information about that forest and the plots recorded.

## 5 CONCLUSION

The forest at Danum, and a large part of the surrounding region in Sabah, is subject to the influences of climatic variability (Newbery et al., 1999; Walsh & Newbery, 1999), a main source of environmental stochasticity that drives forest dynamics (Newbery & Lingenfelder, 2004). Events such as dry ENSO periods introduce marked changes in growth and structure, albeit over relatively short time periods, but ones that have much longer‐term effects (Newbery et al., 2011). Different species’ trees, depending on their size, local environment, and ecophysiology, respond individually and differently in their growth rates. Tree population structures move along trajectories until the next event disturbs them again (Huston, 1994) and throw the interactions again into disarray (Richards, 1996). The responses of the trees, particularly smaller ones, to their larger neighbors, in this changing environment, entail both the focal tree's response to variation in external conditions and the collective changes of the neighbors to it. Existence of conspecific negative density dependence operating on small‐tree growth and survival was called into question by the present study because it was lost when moving from nonspatial to spatial models. Recent re‐evaluation of data from three tropical tree recruitment studies have raised doubts too that this type of dependence is as important in forest dynamics as was previous contended (Detto et al., 2019).

The tropical forest ecosystem cannot necessarily be assumed to be in equilibrium: There is neither reason nor evidence to show that the species populations measured over two decades (1986–2007) would have coexisted in similar proportions in the past or that they will continue likewise to coexist in the future (Tokeshi, 1999). Present dynamics, in terms of tree growth, recruitment, and mortality, are determined by historical contingencies and site conditions. No stabilizing trade‐offs among species need occur either beyond the simple physical constraint of maximum total forest biomass, and possibly a feedback determined within the system by under‐overstory guild structures (Newbery et al., 1992). In the absence then of empirical results, it might be unwise to assume evolutionary strategies operating for root systems (see Dybzinski et al., 2011; McNickle & Dybzinski, 2013). Environmental stochasticity, as pink/red noise in the Danum ENSO signal for instance (Newbery et al., 2011) adds in theory a potentially highly complicated mixing effect on tree‐tree interactions. Furthermore, the continually varying species composition of neighborhoods around individual (focal) trees, temporally and spatially, creates a *neighborhood stochasticity*, which is highly problematic to define, record, model, and use predictively. One clear realization of this was that the change in CON effects on tree growth moving from P_1_ to P_2_ appeared to be partly related to (correlated with) CON and HET effects on survival in P_1_, and yet it is difficult to explain this relationship satisfactorily using the recorded parameters of the species’ mean dynamics and population structures from the same periods.

## CONFLICT OF INTEREST

None declared.

## AUTHOR CONTRIBUTION

**David M. Newbery:** Conceptualization (equal); Data curation (lead); Formal analysis (supporting); Funding acquisition (lead); Investigation (equal); Writing‐original draft (lead). **Peter Stoll:** Conceptualization (equal); Formal analysis (lead); Investigation (equal); Software (lead); Writing‐original draft (supporting).

## Supporting information

Appendix S1‐S7

## Data Availability

The raw input data are available at the Dryad Digital Repository (https://doi.org/10.5061/dryad.q573n5tfx).
