# Supplementary material for: Including tree spatial extension in the evaluation of neighborhood competition effects in Bornean rain forest
Source: Ecol Evol. 2021 May 6;11(11):6195–222. doi: 10.1002/ece3.7452 (PMC8207374; doi:10.1002/ece3.7452)
Supplement: Supplementary file 1 — Appendix S1‐S7 [file ECE3-11-6195-s001.pdf]

Appendix S1: Supporting Table and Figures

[Newbery/Stoll "Including tree spatial extension in the evaluation of neighborhood competition effects in Bornean rain forest"]

Appendix S1: Table S1.

Full list of the 48 species in the two 4-ha plots at Danum combined, the first 38 of which (above the middle solid line) were selected for the more detail analyses and statistical modelling. Grounds for inclusion of the last of the 38, and exclusion of the other 10 species are explained in the main text. Abbreviations ('Abbr.') are used in figures for point labelling species, the matching eight-character codes from the 1986-2007 database are given for reference, with family codes. 'OUI' is the over/understorey index (scale 0-100; see Newbery *et al*. 2011: > 55, overstorey [red], 20-55 intermediate [blue], < 20 understorey [green]). 'BA P_1 and P_2', are the basal area abundances of each species at the plot level (m^2/ha; trees ≥ 10 cm *gbh*), in periods 1 and 2. 'N. trees' shows the numbers of focal trees (10 - < 100 cm *gbh*) that were potentially usable ('pot.'), those excluded ('excl.') where residuals in the *agr* vs. *ba* [individual focus tree basal area] were < −3 SD), and finally those accepted ('used'), for periods 1 and 2. 'N. dead' are numbers of focus trees that died by the ends of periods 1 and 2. The taxonomy follows the stand of the updated database of 2010. On tabulation, the archived data file will give the total counts of focal trees at the starts of P_1 and P_2 which equal the sums of the counts of potential and dead trees (e.g. APORFALC/P_1, *n*_total = 118 + 20 = 138).

						BA		N. trees P1			N. trees P2			N. dead	
Genus	Species	Abbr.	Code	Family	OUI	P_1	P_2	pot.	excl.	used	pot.	excl.	used	P_1	P_2
Aporosa	*falcifera*	Afa	APORFALC	EUPH	54.1	0.43	0.41	118	0	118	114	-7	107	20	19
Ardisia	*sanguinolenta*	Asa	ARDISANG	MYRS	6.8	0.19	0.20	274	-10	264	266	-13	253	51	71
Baccaurea	*tetrandra*	Bte	BACCTETR	EUPH	40.8	0.24	0.24	106	-3	103	92	-4	88	17	19
Chisocheton	*sarawakanus*	Csa	CHISSARA	MELI	49.0	0.25	0.26	78	-1	77	72	-2	70	9	12
Cleistanthus	*contractus*	Cco	CLEICONT	EUPH	3.0	0.08	0.09	146	-2	144	142	-5	137	11	16
Dacryodes	*rostrata*	Dro	DACRROST	BURS	26.1	0.12	0.13	83	0	83	74	-3	71	10	11
Dehaasia	*gigantocarpa*	Dgi	DEHAGIGA	LAUR	15.6	0.07	0.07	68	-3	65	63	0	63	7	9
Dimorphocalyx	*muricatus*	Dmu	DIMOMURI	EUPH	9.8	0.34	0.33	407	-11	396	370	-6	364	55	63
Dysoxylum	*cyrtobotryum*	Dcy	DYSOCYRT	MELI	50.2	0.25	0.26	74	-1	73	65	-1	64	15	12
Fordia	*splendidissima*	Fsp	FORDSPLE	LEGU	4.0	0.16	0.18	251	-7	244	250	-5	245	32	65
Hydnocarpus	*borneensis*	Hbo	HYDNBORN	FLAC	10.6	0.04	0.04	58	0	58	59	-4	55	6	5
Lithocarpus	*gracilis*	Lgr	LITHGRAC	FAGA	64.3	0.18	0.28	20	0	20	13	0	13	14	10
Lithocarpus	*leptogyne*	Lle	LITHLEPT	FAGA	56.5	0.17	0.23	27	-1	26	31	0	31	9	9
Lithocarpus	*nieuwenhuisii*	Lni	LITHNIEW	FAGA	57.0	0.23	0.22	61	-4	57	55	-5	50	7	11
Litsea	*caulocarpa*	Lca	LITSCAUL	LAUR	16.4	0.15	0.15	140	-4	136	110	-1	109	53	77
Litsea	*ochracea*	Loc	LITSOCHR	LAUR	27.7	0.17	0.17	68	-3	65	53	-1	52	19	24
Lophopetalum	*beccarianum*	Lbe	LOPHBECC	CELA	18.3	0.13	0.15	149	-1	148	167	-7	160	15	18
Madhuca	*korthalsii*	Mko	MADHKORT	SAPO	32.1	0.70	0.69	266	-8	258	265	-5	260	20	38
Mallotus	*penangensis*	Mpe	MALLPENA	EUPH	19.4	0.12	0.13	109	-1	108	122	-4	118	14	21
Mallotus	*stipularis*	Mst	MALLSTIP	EUPH	27.9	0.10	0.09	65	0	65	53	0	53	8	17
Mallotus	*wrayi*	Mwr	MALLWRAY	EUPH	10.4	0.95	0.94	1031	-11	1020	972	-15	957	189	217
Maschalocorymbus	*corymbosus*	Mco	MASCCORY	RUBI	0.8	0.11	0.09	144	-2	142	128	-1	127	71	49
Neoscortechinia	*philippinensis*	Nph	NEOSPHIL	EUPH	70.2	0.34	0.33	44	0	44	36	0	36	7	9
Parashorea	*malaanonan*	Pma	PARAMALA	DIPT	65.1	0.71	0.90	51	0	51	37	0	37	14	19
Pentace	*laxiflora*	Pla	PENTLAXI	TILI	63.0	0.42	0.56	103	-4	99	75	-2	73	23	36
Polyalthia	*cauliflora*	Pca	POLYCAUL	ANNO	11.9	0.15	0.14	160	-1	159	147	-2	145	28	26

Polyalthia	*rumphii*	Pru	POLYRUMP	ANNO	20.0	0.07	0.08	78	-1	77	74	-1	73	6	13
Polyalthia	*sumatrana*	Psu	POLYSUMA	ANNO	44.0	0.34	0.34	113	-1	112	114	-3	111	12	13
Polyalthia	*xanthopetala*	Pxa	POLYXANT	ANNO	14.8	0.14	0.11	93	-2	91	81	-4	77	31	45
Reinwardtiodendron	*humile*	Rhu	REINHUMI	MELI	18.1	0.14	0.13	112	-3	109	72	-3	69	34	48
Shorea	*fallax*	Sfa	SHORFALL	DIPT	39.1	0.93	0.94	182	-1	181	151	-4	147	58	79
Shorea	*johorensis*	Sjo	SHORJOHO	DIPT	87.0	3.17	3.83	51	0	51	32	-1	31	37	31
Shorea	*parvifolia*	Spar	SHORPARF	DIPT	86.6	2.32	2.49	51	0	51	40	0	40	32	22
Shorea	*pauciflora*	Spau	SHORPAUC	DIPT	65.8	1.17	1.09	42	0	42	42	0	42	18	17
Shorea	*pilosa*	Spi	SHORPILO	DIPT	69.8	0.97	1.00	59	0	59	52	0	52	13	21
Syzygium	*elopurae*	Sel	SYZYELOP	MYRT	23.2	0.10	0.09	66	-1	65	53	-2	51	15	18
Syzygium	*tawaense*	Sta	SYZYTAWA	MYRT	57.7	0.44	0.50	35	0	35	32	-3	29	8	6
Xanthophyllum	*vitellinum*	Xvi	XANTVITE	POLY	28.6	0.10	0.10	72	-2	70	65	-1	64	5	7
Barringtonia	*lanceolata*	Bla	BARRLANC	LECY	60.4	0.35	0.35	81	-1	80	74	-2	72	1	10
Dipterocarpus	*kerrii*	Dke	DIPTKERR	DIPT	56.0	0.24	0.28	24	0	24	22	0	22	1	5
Drypetes	*longifolia*	Dlo	DRYPLONG	EUPH	55.3	0.14	0.12	28	0	28	25	0	25	3	7
Gonystylus	*keithii*	Gke	GONYKEIT	THYM	42.2	0.24	0.25	61	-1	60	60	-1	59	0	9
Knema	*latericia*	Kla	KNEMLATE	MYRI	5.1	0.04	0.05	83	0	83	95	-3	92	2	7
Microcos	*reticulata*	Mre	MICRRETI	TILI	60.6	0.12	0.11	23	-1	22	19	0	19	4	7
Ochanostachys	*amentacea*	Oam	OCHAAMEN	OLAC	59.5	0.24	0.22	25	0	25	19	0	19	3	7
Scorodocarpus	*borneensis*	Sbo	SCORBORN	OLAC	84.8	0.92	0.89	27	-1	26	23	-1	22	5	7
Syzygium	*lineata*	Sli	SYZYLINE	MYRT	63.0	0.29	0.33	22	0	22	24	-1	23	3	3
Vatica	*dulitensis*	Vcu	VATIDULI	DIPT	45.1	0.37	0.36	53	-2	51	52	-2	50	4	6

Appendix S1: Table S2. Number of species (out of 38) for which regression models are > 7 ΔAIC_c worse/better than, or ≤ 7 ΔAIC_c indifferent from, the *ba* + ALL model with linear distance decay as a reference (0/0/38). The dependent variable was (a) absolute growth rate, or (b) survival (0/1), in period 1 (P_1, 1986 – 1996) or period 2 (P_2, 1996 – 2007). Numbers of species are given for models with size only (*ba*), then with one neighbour term added (*ba* + ALL), or two neighbourhood terms added (*ba* + CON + HET), as non-spatial models; and for the two neighbour terms with one of each of the eight different forms of crown extension (with [no], linear [lin] or squared [squ] distance decay), as spatial models. Models with two neighbour terms and spatial extension were allowed to have different Δd's for their CON and HET terms. Zones of influence of individual trees were filled either with equal numbers of points per unit crown area (*ppsqmca*: 'equal') or such that large trees had more points per unit crown area than smaller trees (*ppsqmca*: 'larger > smaller'), as a function of stem *gbh* (see Methods). Covered modules (i.e. modules with modules of bigger trees within a given distance) were either removed (pruned) or relocated, and the trees position (pos) taken as being at either the original stem coordinates or the centre of gravity of the uncovered points.

**(a) Growth**				Period 1			Period 2		
				Decay			Decay		
Model	*ppsqmca*	covered	pos.	no	lin	squ	no	lin	squ
ba	–	–	–	10/ 0/28	–	–	5/ 0/33	–	–
ba + ALL	–	–	–	0/ 0/38	0/ 0/38	0/ 0/38	0/ 0/38	0/ 0/38	0/ 0/38
ba + CON + HET	–	–	–	0/ 6/32	0/ 4/34	1/ 4/33	0/ 3/35	0/ 4/34	0/ 1/37
	equal	removed	stem	0/12/26	0/13/25	2/ 7/29	0/11/27	2/ 9/27	4/ 6/28
	"	"	crown	0/14/24	0/14/24	2/11/25	0/11/27	0/11/27	1/ 6/31
	"	relocated	stem	0/14/24	0/11/27	1/ 9/28	1/13/24	2/14/22	3/ 5/30
	"	"	crown	0/14/24	0/11/27	1/10/27	1/14/23	1/14/23	2/ 7/29
	larger > smaller	removed	stem	0/10/28	2/ 9/27	4/ 8/26	3/ 7/28	3/ 7/28	4/ 6/28
	"	"	crown	0/ 9/29	2/ 8/28	4/ 8/26	2/ 7/29	2/ 8/28	3/ 7/28
	"	relocated	stem	0/11/27	1/ 9/28	2/ 8/28	2/ 8/28	2/ 8/28	4/ 7/27
	"	"	crown	0/12/26	0/10/28	0/ 9/29	2/ 7/29	2/ 7/29	3/ 6/29

**(b) Survival**				Period 1			Period 2		
				Decay			Decay		
Model	*ppsqmca*	covered	pos.	no	lin	squ	no	lin	squ
ba	–	–	–	2/ 0/36	–	–	2/ 0/36	–	–
ba + ALL	–	–	–	0/ 0/38	0/ 0/38	0/ 0/38	0/ 0/38	0/ 0/38	0/ 0/38
ba + CON + HET	–	–	–	0/ 2/36	0/ 0/38	0/ 0/38	0/ 1/37	0/ 1/37	1/ 1/36
	equal	removed	stem	0/ 5/33	0/ 2/36	4/ 2/32	0/ 9/29	0/ 6/32	2/ 5/31
	"	"	crown	0/ 5/33	0/ 4/34	3/ 3/32	0/ 9/29	0/ 5/33	2/ 4/32
	"	relocated	stem	1/ 4/33	1/ 2/35	2/ 2/34	0/11/27	0/ 7/31	1/ 4/33
	"	"	crown	1/ 4/33	1/ 2/35	2/ 1/35	0/ 9/29	0/ 7/31	1/ 2/35
	larger > smaller	removed	stem	0/ 6/32	1/ 2/35	2/ 1/35	0/ 5/33	1/ 4/33	1/ 3/34
	"	"	crown	0/ 6/32	0/ 5/33	1/ 3/34	0/ 4/34	0/ 3/35	1/ 3/34
	"	relocated	stem	0/ 4/34	0/ 2/36	1/ 1/36	0/ 5/33	0/ 3/35	1/ 3/34
	"	"	crown	0/ 5/33	0/ 3/35	1/ 2/35	0/ 5/33	0/ 2/36	1/ 2/35

Appendix S1: Table S3. As Table S2 in the main text but with ‘ba + CON + HET’ as the reference model instead of ‘ba + ALL’. Otherwise the table parameters are the same.

**(a) Growth**				Period 1			Period 2		
				Decay			Decay		
Model	*ppsqmca*	covered	pos.	no	lin	squ	no	lin	squ
ba	–	–	–	14/ 0/24	–	–	9/ 0/29	–	–
ba + ALL	–	–	–	5/ 0/33	4/ 0/34	7/ 0/31	1/ 0/37	4/ 0/34	4/ 0/34
ba + CON + HET	–	–	–	0/ 0/38	0/ 0/38	0/ 0/38	0/ 0/38	0/ 0/38	0/ 0/38
	equal	removed	stem	0/10/28	0/10/28	5/ 4/29	1/10/27	0/10/28	4/ 4/30
	“	“	crown	0/11/27	0/ 9/29	5/ 5/28	0/12/26	0/10/28	3/ 5/30
	“	relocated	stem	1/12/25	0/ 9/29	5/ 6/27	1/13/24	1/11/26	3/ 4/31
	“	“	crown	1/12/25	0/10/28	5/ 5/28	1/13/24	1/ 9/28	3/ 5/30
	larger > smaller	removed	stem	0/ 3/35	1/ 3/34	7/ 3/28	2/ 5/31	3/ 5/30	6/ 4/28
	“	“	crown	0/ 4/34	2/ 4/32	6/ 3/29	2/ 5/31	3/ 6/29	5/ 6/27
	“	relocated	stem	0/ 5/33	0/ 3/35	4/ 2/32	2/ 5/31	3/ 5/30	5/ 5/28
	“	“	crown	0/ 4/34	1/ 4/33	4/ 1/33	2/ 6/30	3/ 4/31	4/ 5/29

**(b) Survival**				Period 1			Period 2		
				Decay			Decay		
Model	*ppsqmca*	covered	pos.	no	lin	squ	no	lin	squ
ba	–	–	–	4/ 0/34	–	–	5/ 0/33	–	–
ba + ALL	–	–	–	0/ 0/38	0/ 0/38	1/ 0/37	2/ 0/36	1/ 0/37	2/ 0/36
ba + CON + HET	–	–	–	0/ 0/38	0/ 0/38	0/ 0/38	0/ 0/38	0/ 0/38	0/ 0/38
	equal	removed	stem	0/ 1/37	1/ 0/37	4/ 1/33	0/ 6/32	0/ 1/37	2/ 1/35
	“	“	crown	0/ 1/37	1/ 0/37	2/ 2/34	0/ 7/31	0/ 3/35	1/ 3/34
	“	relocated	stem	1/ 1/36	2/ 0/36	3/ 1/34	0/ 7/31	0/ 4/34	0/ 1/37
	“	“	crown	1/ 1/36	2/ 0/36	3/ 1/34	0/ 7/31	0/ 3/35	0/ 1/37
	larger > smaller	removed	stem	1/ 2/35	1/ 1/36	1/ 0/37	0/ 0/38	0/ 0/38	2/ 1/35
	“	“	crown	1/ 2/35	1/ 1/36	1/ 1/36	0/ 1/37	1/ 1/36	1/ 1/36
	“	relocated	stem	0/ 2/36	1/ 2/35	1/ 1/36	0/ 0/38	0/ 0/38	1/ 1/36
	“	“	crown	0/ 2/36	1/ 2/35	1/ 1/36	0/ 1/37	0/ 1/37	1/ 1/36

Appendix S1: Table S4. Numbers of species reaching increasing levels of (a) variance accounted for, and significance of (b) CON and (c) HET coefficients, averaged across all fitted models within $2\Delta AIC_c$ of the best fitting one; for growth and survival response models in P_1 and P_2. 'Non-spat' is the non-spatial model, and 'relocate' and 'remove' indicate the 'larsm/reloc/crown' and 'larsm/remov/crown' spatial models (linear decay).

[a] mean adj.R^2 (%)			Number of species					
			<2	< 5	< 10	< 20	< 50	≥ 50
Growth	P1	non-spat	0	3	5	8	29	9
		relocate	1	3	5	9	27	11
		remove	1	3	5	9	27	11
	P2	non-spat	0	1	5	11	27	11
		relocate	0	1	3	12	26	12
		remove	1	1	4	12	25	13
Survival	P1	non-spat	8	21	28	35	38	0
		relocate	5	20	25	33	38	0
		remove	10	19	26	35	38	0
	P2	non-spat	5	19	29	33	37	1
		relocate	3	21	29	33	37	1
		remove	3	22	29	35	36	2

[b] mean CON_P-value			Number of species					
			< 0.001	< 0.01	< 0.05	< 0.10	< 0.25	≥ 25
Growth	P1	non-spat	4	8	17	18	24	14
		relocate	10	16	22	25	29	9
		remove	7	16	21	23	31	7
	P2	non-spat	3	5	9	13	22	16
		relocate	5	9	16	20	26	12
		remove	5	9	15	19	24	14
Survival	P1	non-spat	0	1	7	13	19	19
		relocate	0	2	11	17	28	10
		remove	0	2	9	17	25	13
	P2	non-spat	0	2	6	11	17	21
		relocate	1	2	8	12	21	17
		remove	1	2	7	10	18	20

[c] mean HET_P-value			Number of species					
			< 0.001	< 0.01	< 0.05	< 0.10	< 0.25	≥ 25
Growth	P1	non-spat	4	16	22	26	30	8
		relocate	5	9	20	24	25	13
		remove	5	10	15	24	30	8
	P2	non-spat	5	8	17	23	30	8
		relocate	4	5	16	23	28	10
		remove	3	7	17	23	29	9
Survival	P1	non-spat	1	2	6	11	20	18
		relocate	1	3	7	11	17	21
		remove	1	2	4	11	19	19
	P2	non-spat	0	1	8	14	21	17
		relocate	0	1	6	9	24	14
		remove	0	1	6	14	24	14

Appendix S1: Table S5. Ranges of the correlations coefficients, r, found pair-wise among the eight spatial models, with their corresponding *t*-values, for the eight combinations of conspecific vs heterospecific effect size (CON, HET), growth vs survival variables (G, S), and periods 1 and 2 (P_1, P_2). The critical *t*-value for a one-sided test at P = 0.0001 is 4.140 (df = 36).

			r	*t*
CON	G	P1	0.9323 – 0.9956	15.471 – 63.593
		P2	0.9625 – 0.9973	21.303 – 80.880
CON	S	P1	0.6924 – 0.9985	5.757 – 108.17
		P2	0.6707 – 0.9915	5.426 – 45.732
HET	G	P1	0.8594 – 0.9919	10.085 – 46.892
		P2	0.7022 – 0.9647	5.918 – 21.972
HET	S	P1	0.6779 – 0.9899	5.533 – 41.682
		P2	0.3244 – 0.9998	2.058 – 330.47

Appendix S1: Table S6. Correlations (Pearson-*r*) between combinations of conspecific vs heterospecific effect size (CON, HET), growth vs survival variables (G, S), and periods 1 and 2 (P_1, P_2), for the “larsm/**reloc**/crown” (upper triangle) and “larsm/**remov**/crown” (lower triangle) spatial models. T-tests for *r*-values were two-tailed. Coefficients in bold face are significant at $P \leq 0.05$.

	CON.G.P1	CON.G.P2	CON.S.P1	CON.S.P2	HET.G.P1	HET.G.P2	HET.S.P1	HET.S.P2
CON.G.P1		*** **0.521**	0.133	0.027	*****0.548**	º 0.300	0.047	−0.127
CON.G.P2	*****0.545**		−0.234	0.007	***0.363**	****0.440**	***−0.327**	−0.126
CON.S.P1	−0.004	−0.233		0.114	0.089	−0.090	***0.332**	-0.108
CON.S.P2	0.021	−0.032	−0.156		−0.226	0.308	−0.099	−0.047
HET.G.P1	****0.502**	***0.375**	−0.083	−0.021		−0.008	0.077	º0.318
HET.G.P2	0.295	***0.399**	−0.094	−0.111	0.019		−0.122	****−0.433**
HET.S.P1	0.035	−0.300	º0.320	−0.209	0.072	−0.042		−0.026
HET.S.P2	−0.118	−0.089	−0.116	***0.371**	0.196	*****−0.665**	−0.066	

***, $P \leq 0.001$; **, $P \leq 0.01$; *, $P \leq 0.05$; º, $P \leq 0.10$.

Appendix S1: Figure S1. Frequency histograms of (a) correlations (‘cor_’), and (b) slopes (‘slope_’), of the relationships between fitted CON (‘con’) and HET (‘het’) neighborhood regression coefficients versus their corresponding best fitting radii, when modelling either growth (‘g’) or survival (‘s’) focus tree responses, in periods P_1 (‘P1’) or P_2 (‘P2’), for the 38 selected species. In some panels the total frequency is < 38 when any species’ regression had ≤ 2 fitted models within the band 2ΔAICc.

(a) correlations

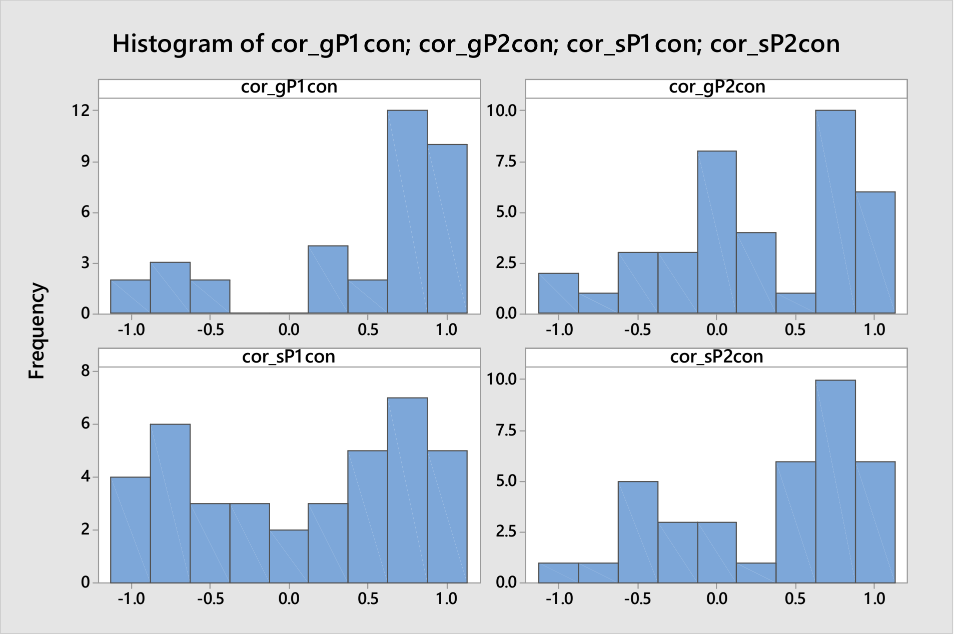

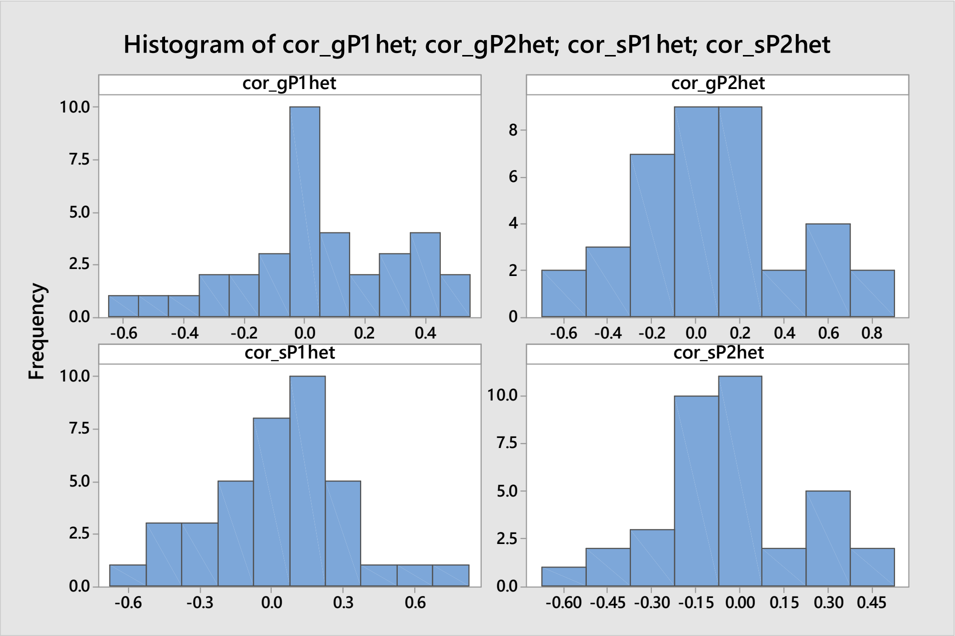

(b) slopes

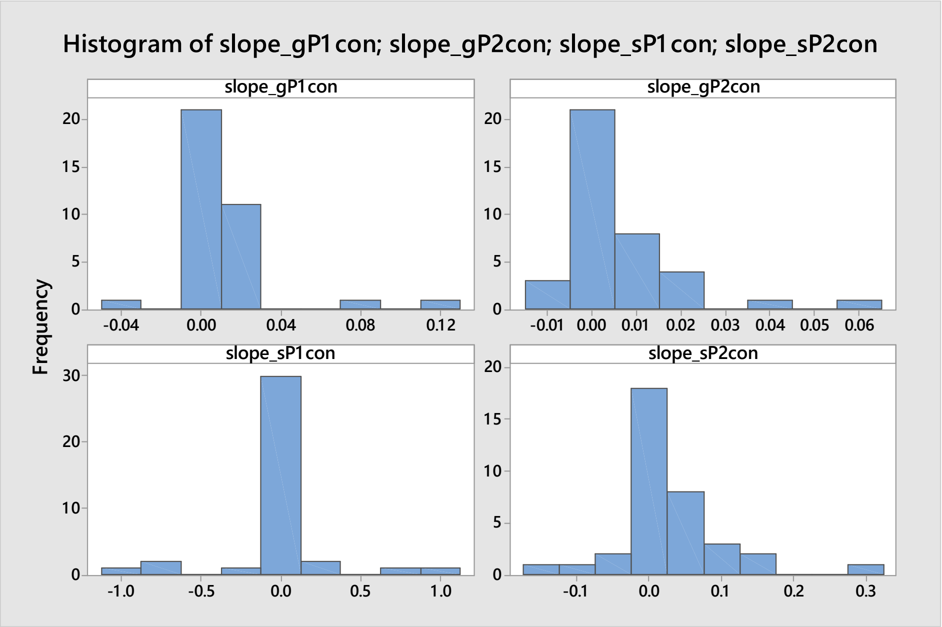

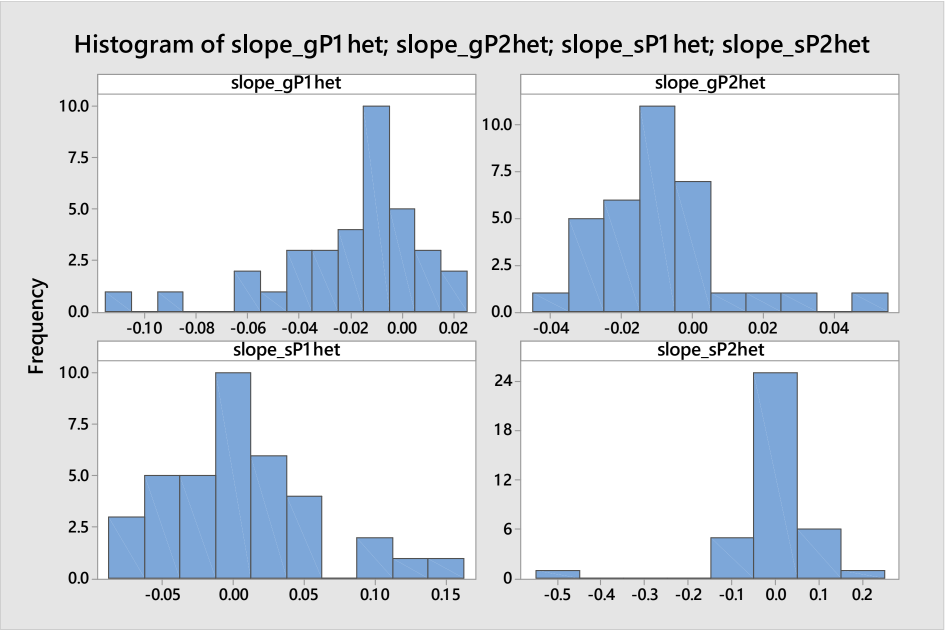

Note: In the lower left panel of the upper set of (b), i.e. for 'slope_sP1con', the five cases mentioned in the text with very outlying slopes were *Dimorphocalyx muricatus*, *Neoscortechinia philippinensis*, and *Parashorea malaanonan* (negative), and *Polyalthia rumphii* and *Ardisia sanguinolenta* (positive).

Appendix S1: Figure S2. Frequency histograms of mean Δd_{CON} and Δd_{HET}-values corresponding to the best fitting 'CON' and 'HET' neighborhood regression coefficients when modelling either growth ('g') or survival ('s') focus tree responses, in periods P_1 ('P1') or P_2 ('P2'), for the 38 selected species, using the spatial models 'larsm/crown/remov' and (b) 'larsm/crown/reloc'. The means were those over all model fits per species within the band 2ΔAICc.

(a) Point adjustment by removal

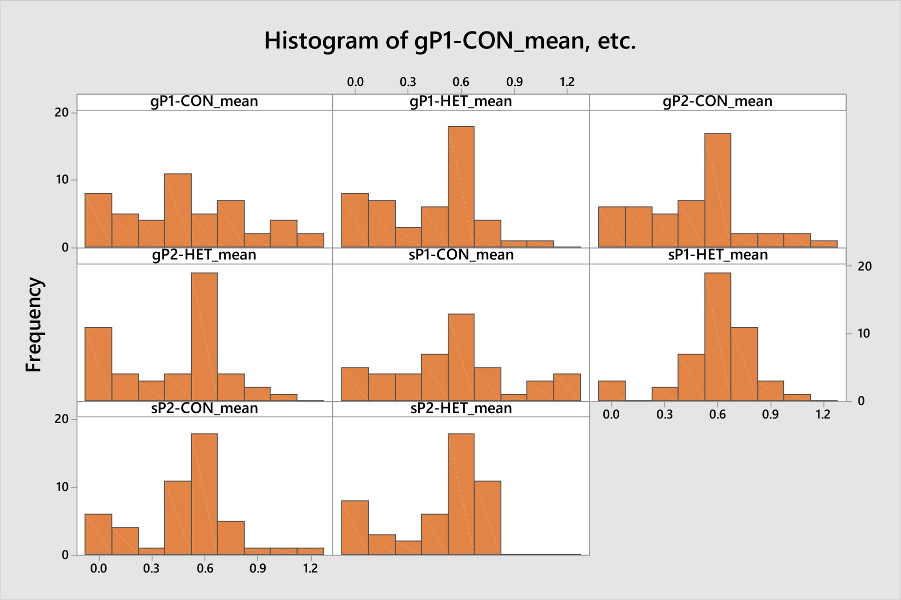

(b) Point adjustment by relocation

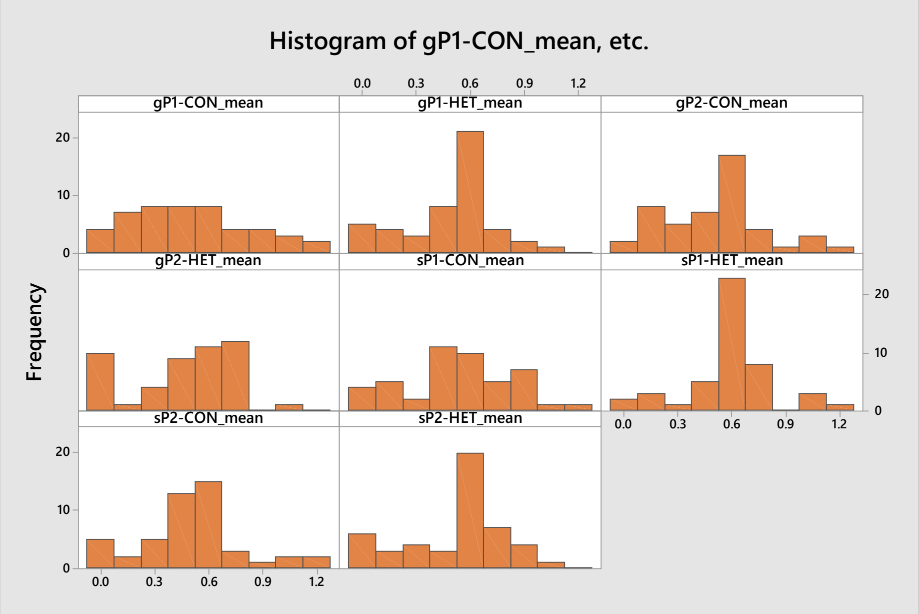

Appendix S1: Figure S3. Relationships between *differences* in conspecific (CON) effect sizes on growth rates between periods ($P_2 - P_1$) and (a) CON and (b) HET effects on survival in period 1 for the 38 species, from non-spatial neighbourhood models with size (*ba*) and two neighbour terms (HET and CON). These figures complement the spatial models in Figs. 2 and 3 in main text, using the same labelling and color-coding.

(a)

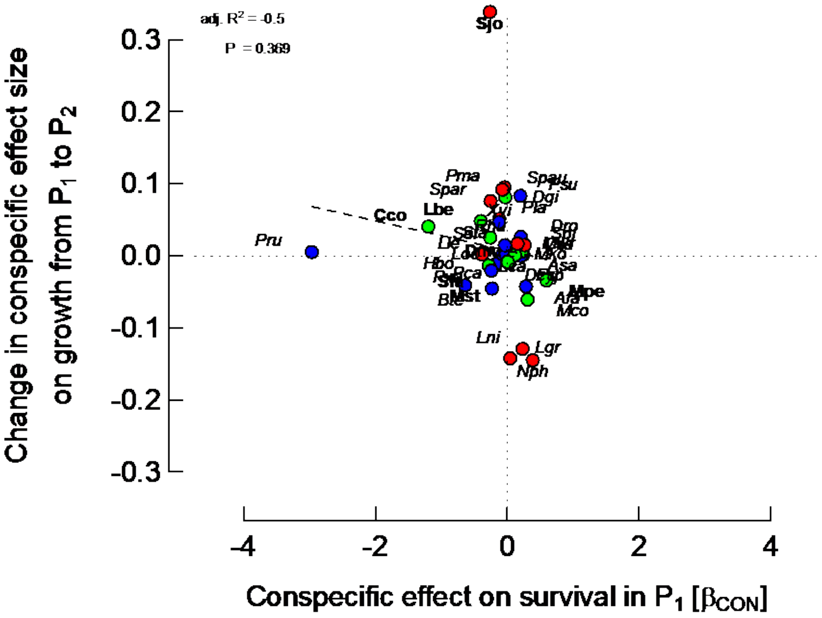

(b)

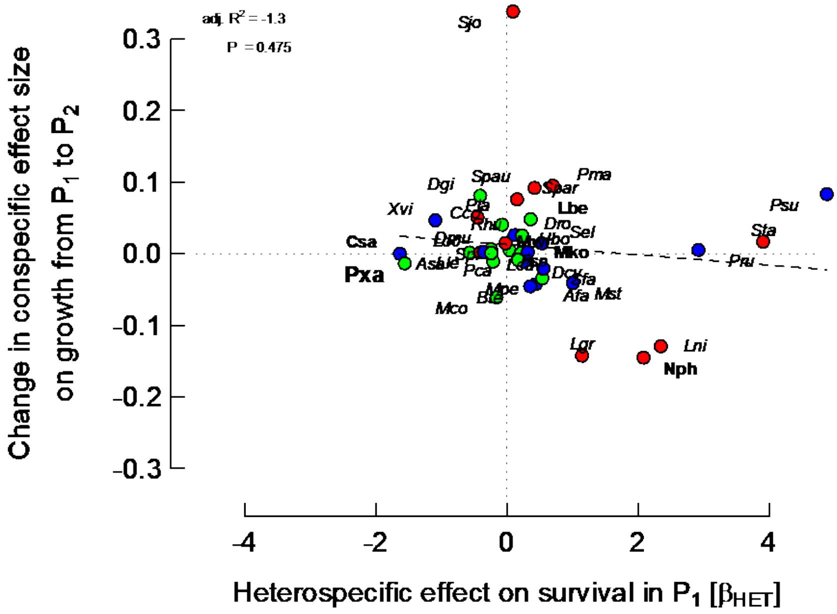

Appendix S1: Figure S4. Relationships between *differences* in heterospecific (HET) effect sizes on growth rates between periods ($P_2 - P_1$) and (a) CON and (b) HET effects on survival in period 1 for the 38 species, from non-spatial neighbourhood models with size (*ba*) and two neighbour terms (HET and CON). These figures complement the spatial models in Figs. 4 and 5 in main text, using the same labelling and color-coding.

(a)

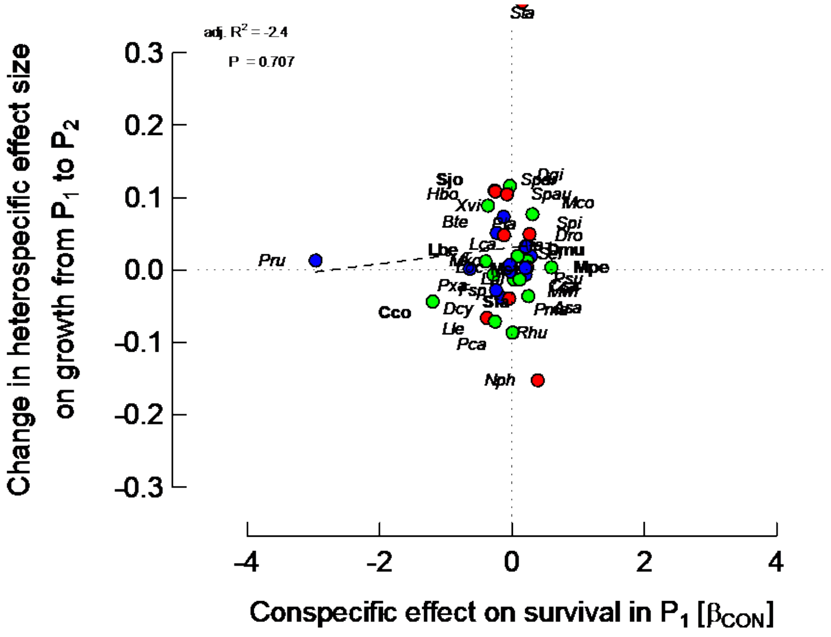

(b)

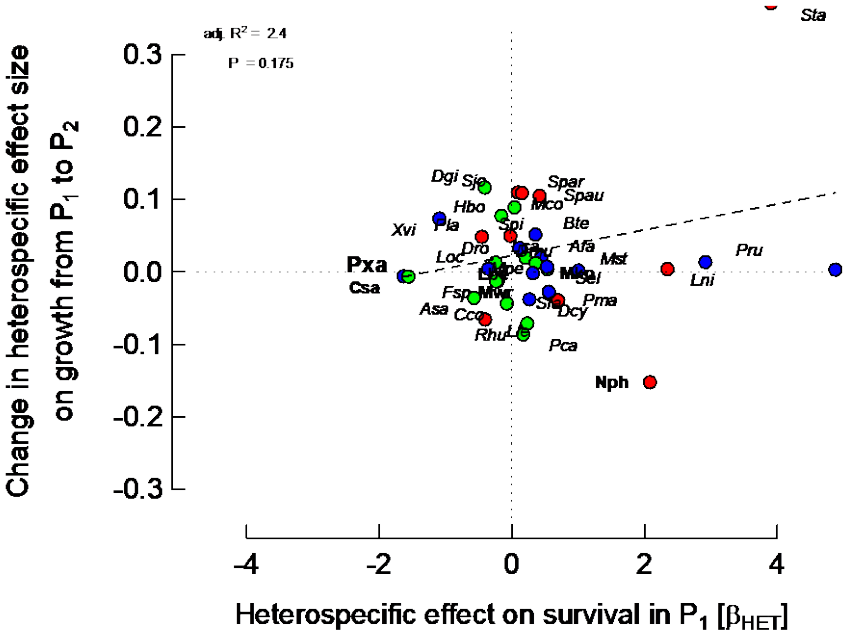

Appendix S1: Figure S5. Relationships between conspecific (CON) effect sizes on growth rates in periods (a) 1, and (b) 2, and CON effects on survival in period 1 for the 38 species, from the ‘larsm/reloc/crown’ spatial neighbourhood model with size (*ba*) and two neighbour terms (HET and CON). These figures complement the spatial models in Fig. 2 (b) in main text, using the same labelling and color-coding.

(a)

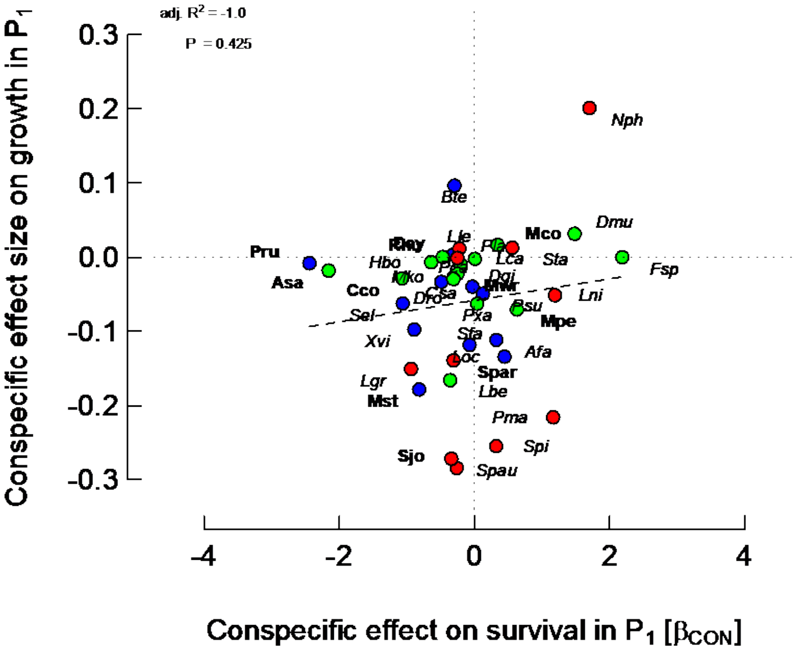

(b)

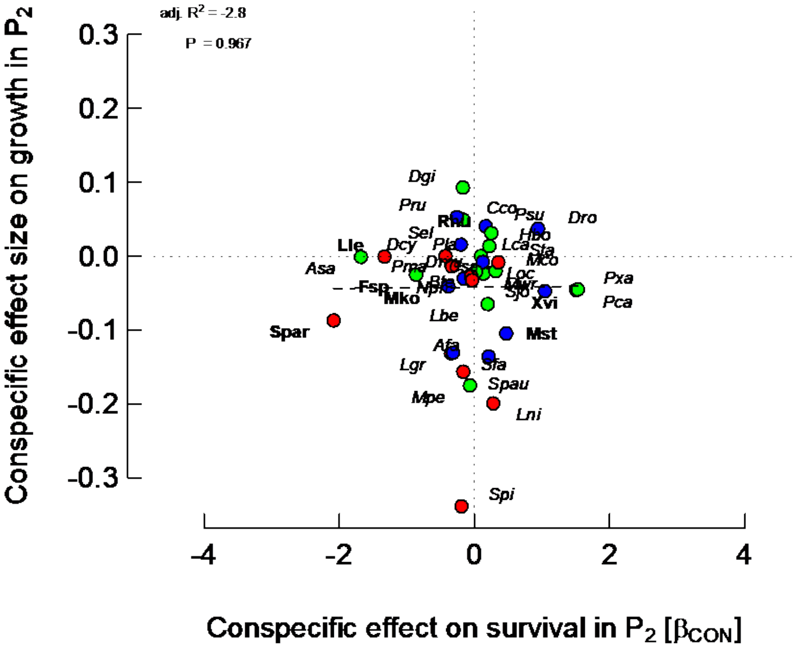

Appendix S1: Figure S6. Relationships between (a) conspecific (CON), and (b) heterospecific (HET) effect sizes on survival in period 1, for the 38 species, from the spatial ('larsm/crown/reloc') versus non-spatial models with size (*ba*) and two neighbour terms (HET and CON). Important outliers: (a) Pru, and (b) Pru, Sta, Psu (see main text).

(a)

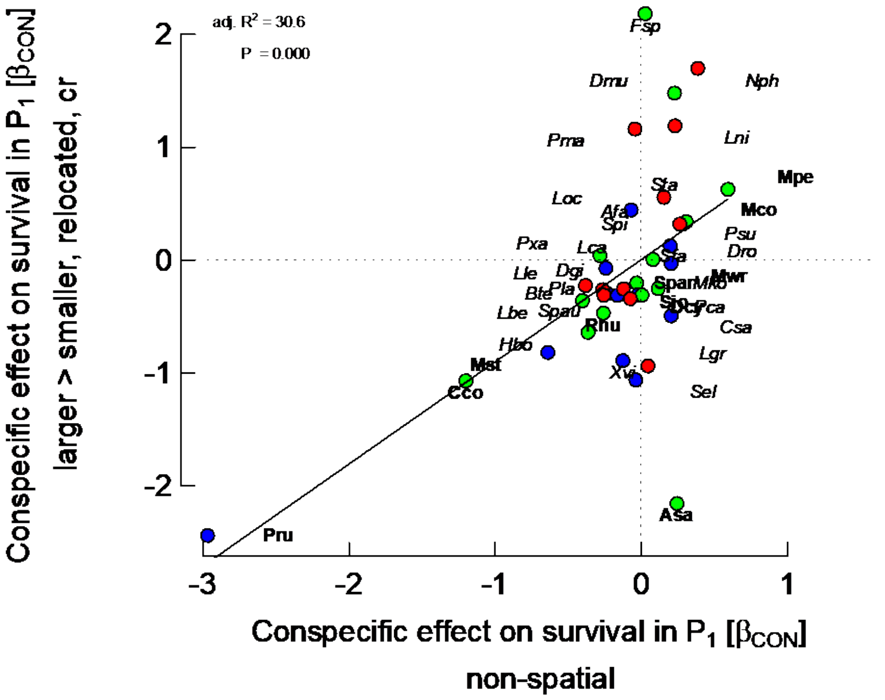

(b)

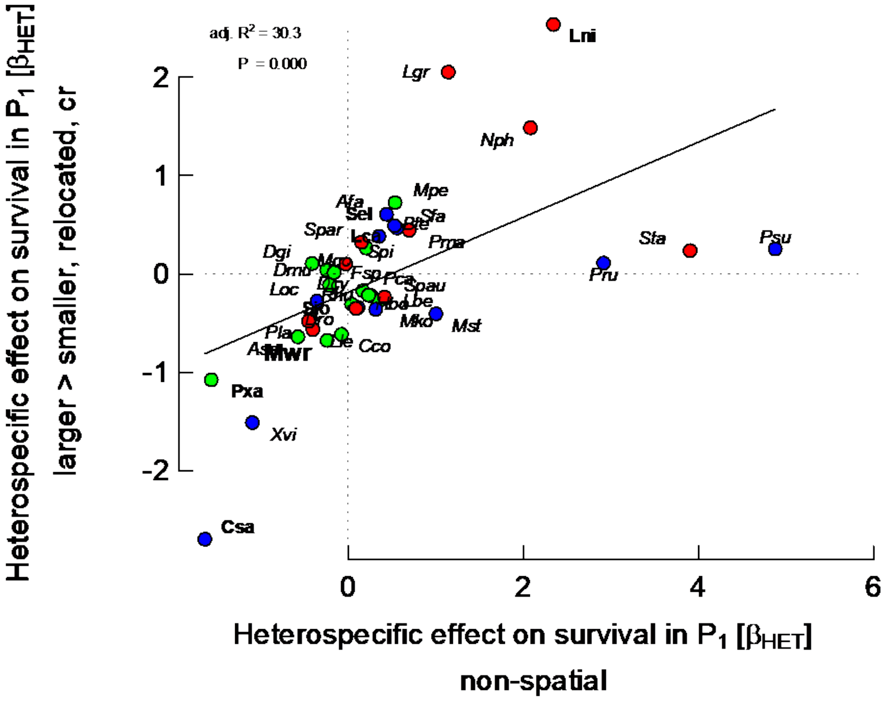

Appendix S2**:** explaining the algorithms used to extend trees spatially, taking potential plasticity into account and evaluate their neighbourhoods.

[Newbery/Stoll “Including tree spatial extension in the evaluation of neighborhood competition effects in Bornean rain forest”]

The **R-Scripts** in folder **GitHub** are intended to demonstrate the principles. Their functionality is exactly as those used in the analyses presented in the main text. However, they were simplified as far as possible to illustrate their functionality. The data in **FULL_test.txt** are constructed to facilitate understanding of the functionality of the algorithms and are not meant to imply any biological meaning. The scripts are available at https://github.com/stollp/nncrexm, under a **Creative Commons** (CC) **license** without any warranty or support. However, the author may be contacted for informal exchange of ideas.

FULL_test.txt

Plot	Tag	X	Y	F_Code07	Code07	GBH.86	GBH.96	GBH.01	GBH.07	col
MP1	1	5.0	5.0	EUPH	MALLWRAY	50	13.4	0.0	NA	"red"
MP1	2	15.0	5.0	MYRT	SYZYELOP	175	112.8	113.2	0	"green3"
MP1	3	5.0	15.0	LAUR	BEILGLAU	200	18.4	0.0	NA	"blue"
MP1	4	12.1	12.1	DIPT	SHORJOHO	300	18.4	0.0	NA	"orange"
MP1	5	12.1	10.0	EUPH	MALLWRAY	50	13.4	0.0	NA	"red"

GBH.YEAR: girth at breast height. GBH.96, .01, and .07 have no meaning and are only kept for consistency with the original data file. For example, the condition GBH.96 > 0 was used in some of the scripts to take only living trees as neighbours.

Figure S1. From abstract, mathematical to impressionistic representation of trees with crown plasticity in neighbourhood models. *This figure is similar to Fig. 1 in the main text but the point density is fixed for showing how the algorithms work.* (a) Classical neighbourhood models represent trees as points without any spatial extension. Taking the red tree at X/Y = 5/5 m as focal tree, it has no neighbours at 5 m neighbourhood radius, four (1 con and 3 het) within 15 m and, depending on the exact definition of neighbours (i.e. < 10 or ≤ 10 m), one or four neighbours at 10 m neighbourhood radius. (b) Impressionistic representation of tree crowns as circles filled with 100 points (to facilitate counting) each. The crown radii (*cr*, in m) were allometrically related to girth at breast height (*gbh*, in cm). Girth of the smallest trees (red at 5/5 and 12.1/10) are 50 cm, those of their neighbours in increasing girth order 175 cm (green at 15/5), 200 cm (blue at 5/15) and 300 cm (orange at 12.1/12.1). These girths correspond to crown radii of 2.7, 5.9, 6.4 and 8.1 m, respectively (all-species regression, Table 1). Coordinates of points filling the crowns (or ZOI) were random. For the analyses presented in the main text, crowns were either filled with as many points as the trees basal area at breast height (*ba*, in cm^2) or 10 points per unit crown area. Filling was realised by ‘blowing up’ the original data file with as many rows per individual tree as points within the crown and initially flagging each point as ‘uncovered’. All three bigger heterospecific neighbours of the focal tree at 5/5 now have at least parts of their crowns already within the focal trees 5 m

neighbourhood (4 blue, 1 green and 9 yellow = 14). The total number of conspecific points with in each 15 m neighbourhood of the red focals is 100 points. The total number of heterospecific points within each 20 m neighbourhoods of the red focals should be 300 as demonstrated in the following Output from R-Script 2_NN.R using function eval_NN.R.

r	Tag	x	y	nos	n_c	n_C	n_h	n_H	a_c	o_C	o_h	o_H	l_c	l_C	l_h	l_H
1	1	5.0	5	0	0	0	0	0	0	0	0	0	0.0	0	0	0.0
5	1	5.0	5	3	0	0	0	14	0	0	0	14	0.0	0	0	3.7
10	1	5.0	5	4	82	0	0	139	82	0	0	139	10.2	0	0	20.5
15	1	5.0	5	4	100	0	0	270	100	0	0	270	11.9	0	0	31.2
20	1	5.0	5	4	100	0	0	300	100	0	0	300	11.9	0	0	33.1
1	5	12.1	10	1	0	0	0	1	0	0	0	1	0.0	0	0	1.2
5	5	12.1	10	3	0	0	0	70	0	0	0	70	0.0	0	0	23.7
10	5	12.1	10	4	78	0	0	247	78	0	0	247	9.4	0	0	48.7
15	5	12.1	10	4	100	0	0	300	100	0	0	300	11.5	0	0	53.3
20	5	12.1	10	4	100	0	0	300	100	0	0	300	11.5	0	0	53.3

r: neighbourhood radius, nos: number of species, n_ number of points of smaller or equally sized conspecifics (_c), heterospecifics (_h) and bigger con (_C) or heterospecific (_H) neighbours. o_ sum of points ('basal area') of neighbours (the number of points), l_ sum of points weighed by distance.

Taking plasticity into account

Points within ZOI's may have points of bigger neighbours within their immediate neighbourhood as a function of some distance (Δd). Therefore, each points local neighbourhood is evaluated within Δd (0, 0.2, …, 1.2). Δd = 0 would allow complete overlap (as shown in b), whereas larger values of Δd flag individual points (open symbols in d)) as being 'shaded' if they have points of bigger neighbours within Δd. If a tree is completely covered as the red focal at 12.1/10, it is still used as focal, but from the perspective of the red focal at 5/5 it 'disappears' completely only as neighbour.

Two possibilities are used to handle these shaded points. First, (in c) the points are simply completely removed (*pruned*). Second, (in d), in an attempt to mimic plasticity, shaded points are relocated to unshaded parts of the crown using two-dimensional contour functions (function `kde2d` in MASS) to find the outline of these points. These contours were allowed to be larger as the original crowns by evaluating density contour lines using contourLines(Den, levels = c(0, 0.001, 0.01, 0.05, .1, 0.25, 0.5, 1)) and of these taking the lowest density levels at the outer edges of the crowns. The total number of points is reduced in c) but remains unaltered in d). Note that removal or relocation is only relevant for trees in their 'role' as neighbours. As focals, trees have a position and the neighbourhood is evaluated around this position given by the trees coordinate no matter whether or not it is completely covered. That is to say that figures 1c) and 1d) with Δd = 0, would look exactly as figure 1b), because not a single point would have a point of a bigger neighbour

exactly on top of itself. The neighbourhood of each tree is evaluated by counting the number of points of crowns of other trees as a function of the focal tree's neighbourhood radius. The focal tree's position is either taken as the original stem coordinates or, alternatively, as the centroid (not shown) of the unshaded part of its crown. For the largest tree (orange) these two positions coincide.

The **R-scripts** and functions in the **GitHub** repository are described in the **README.pdf** and may be used to reproduce Fig. 1 using the small test data set (FULL_test.txt).

Executing **0_Impressionistic.R** produces binary files Fig_1_xxppca_c_yy_d_zz

xx: Number of points per crown
yy: non-spatial 0.00, spatial 1.00
zz: delta_d (0 = complete overlap, 1.2 highly assymetric)

The spatial version (FIG_1_100ppca_c_1.00_d_1.20) used to draw Fig. 1 as shown is included and may be directly used to redraw Fig. 1.

Executing **Fig_1.R** produces the figure, evaluates the neighbourhood of the red focals with Tags 1 and 5 (sourcing(**1_Loop.R**) and **2_NN.R**) and outputs their neighbourhood information at neighbourhood radii of 1, 5, 10, 15 and 20 m.

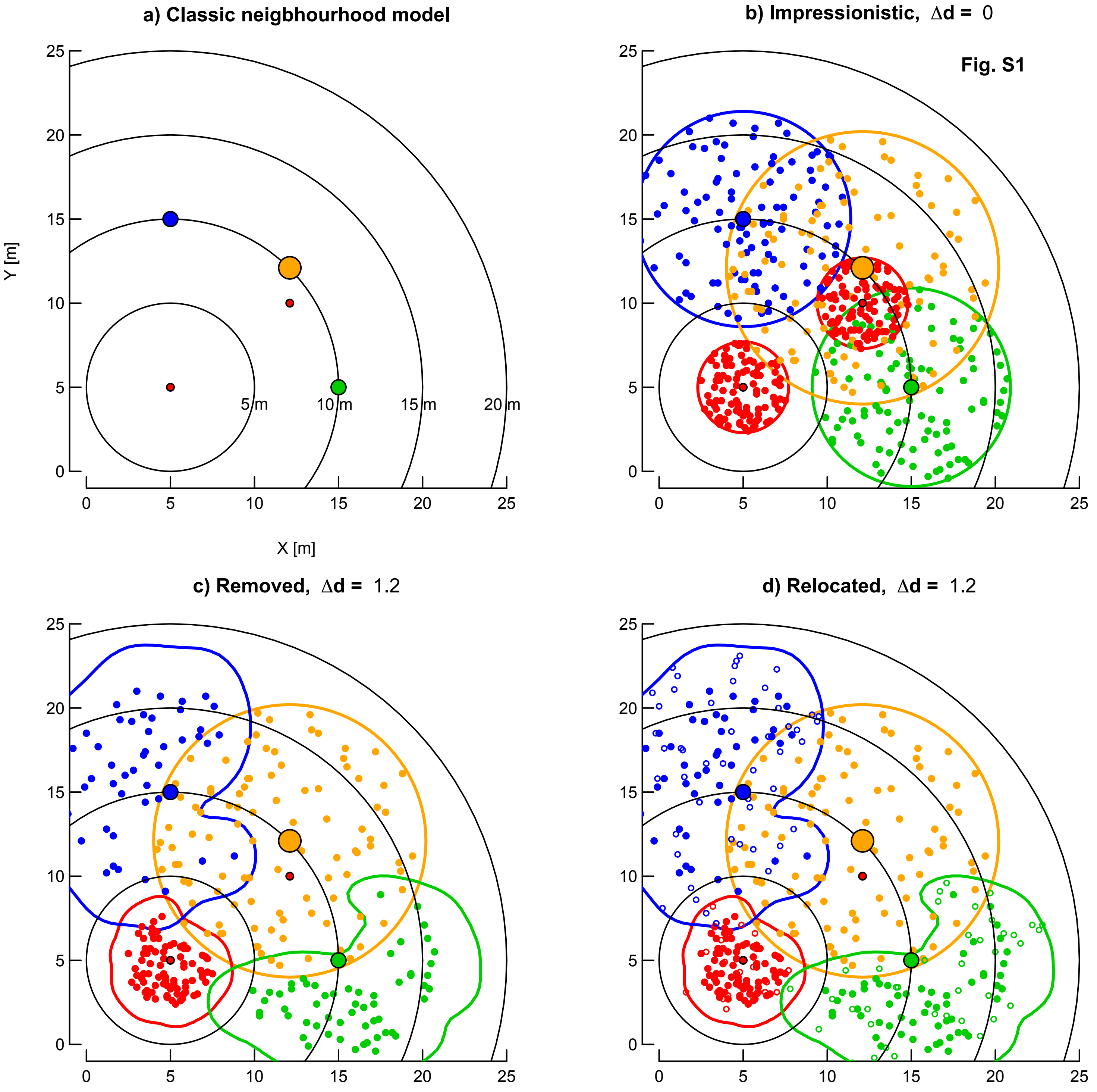
a) Classic neigbhourhood model
b) Impressionistic, Δd = 0
Fig. S1
c) Removed, Δd = 1.2
d) Relocated, Δd = 1.2
Y [m]
X [m]
5 m
10 m
15 m
20 m
0
5
10
15
20
25

Appendix S3. Frequency distributions of the fitted CON coefficients for the 48 species neighbourhood (lin-decay) 'larsm/reloc/crown' models:

[Newbery/Stoll “Including tree spatial extension in the evaluation of neighborhood competition effects in Bornean rain forest”]

Fig. S1A growth in P_1
S1B growth in P_2
S2A survival in P_1
S2B survival in P_2

Count is the number of fitted combinations of radius and Δd fits within the range of 2ΔAICc.

App_3:Fig_S1A (growth P1) − page 1

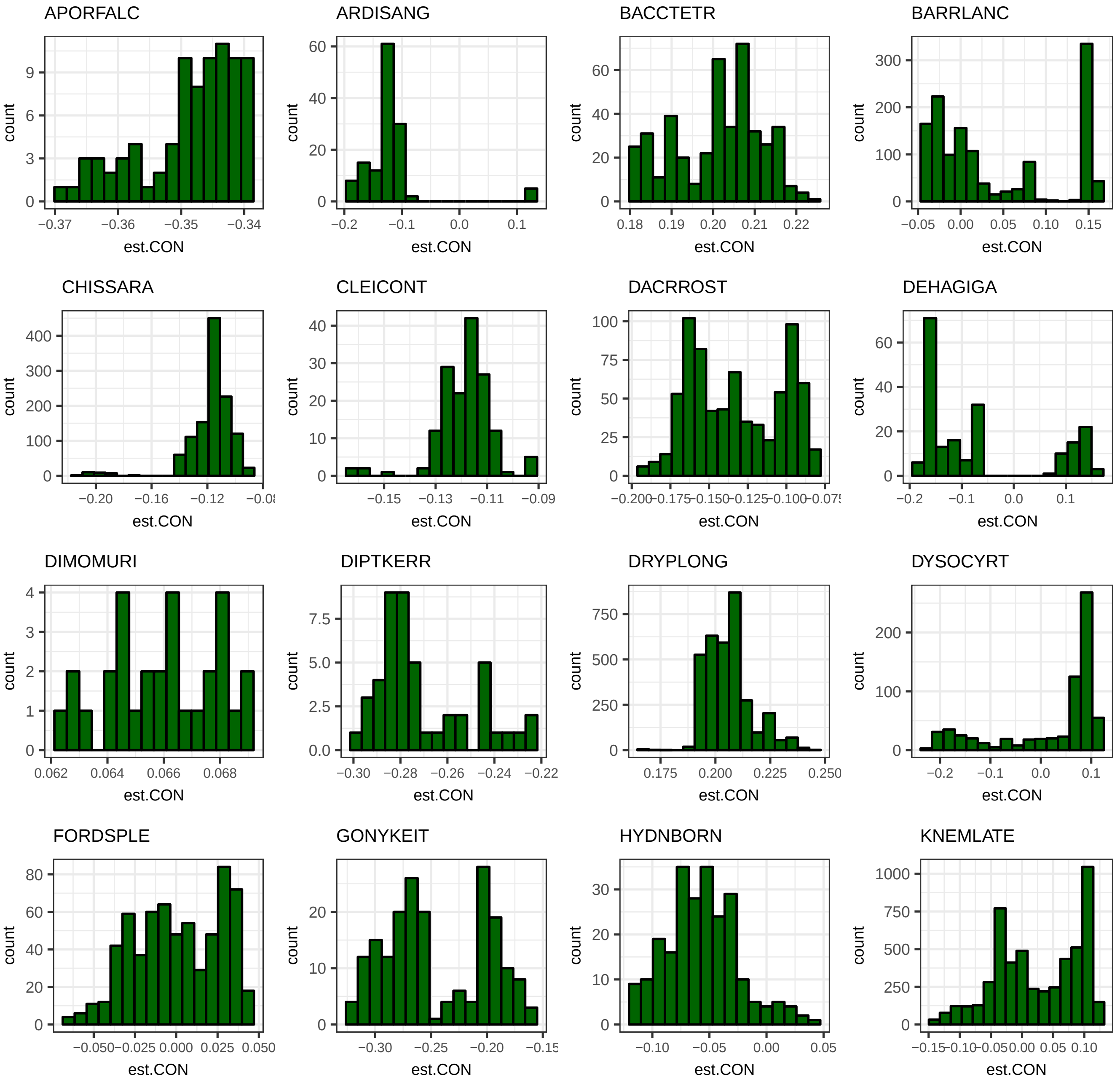

App_3:Fig_S1A (growth P1) − page 2

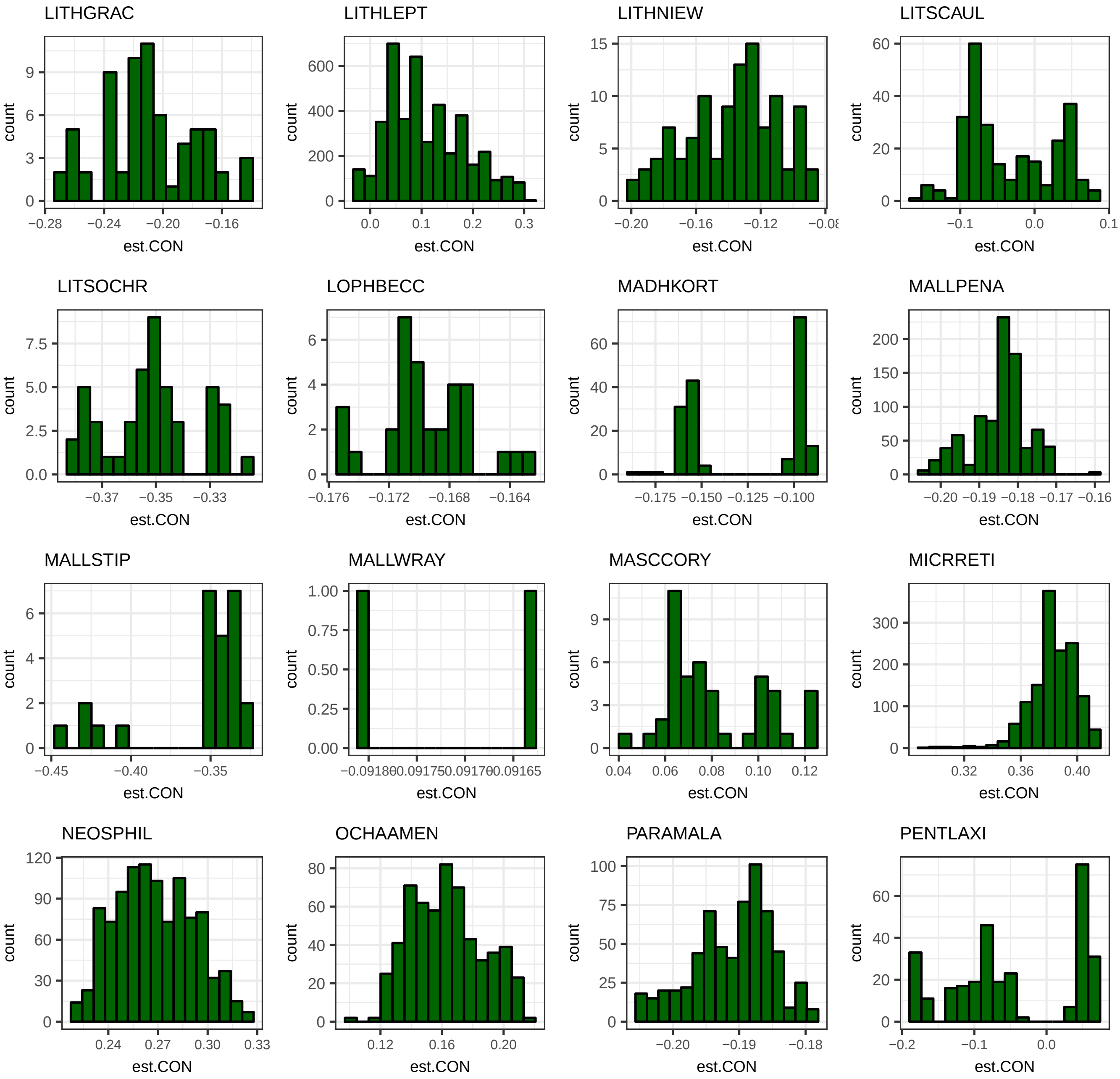

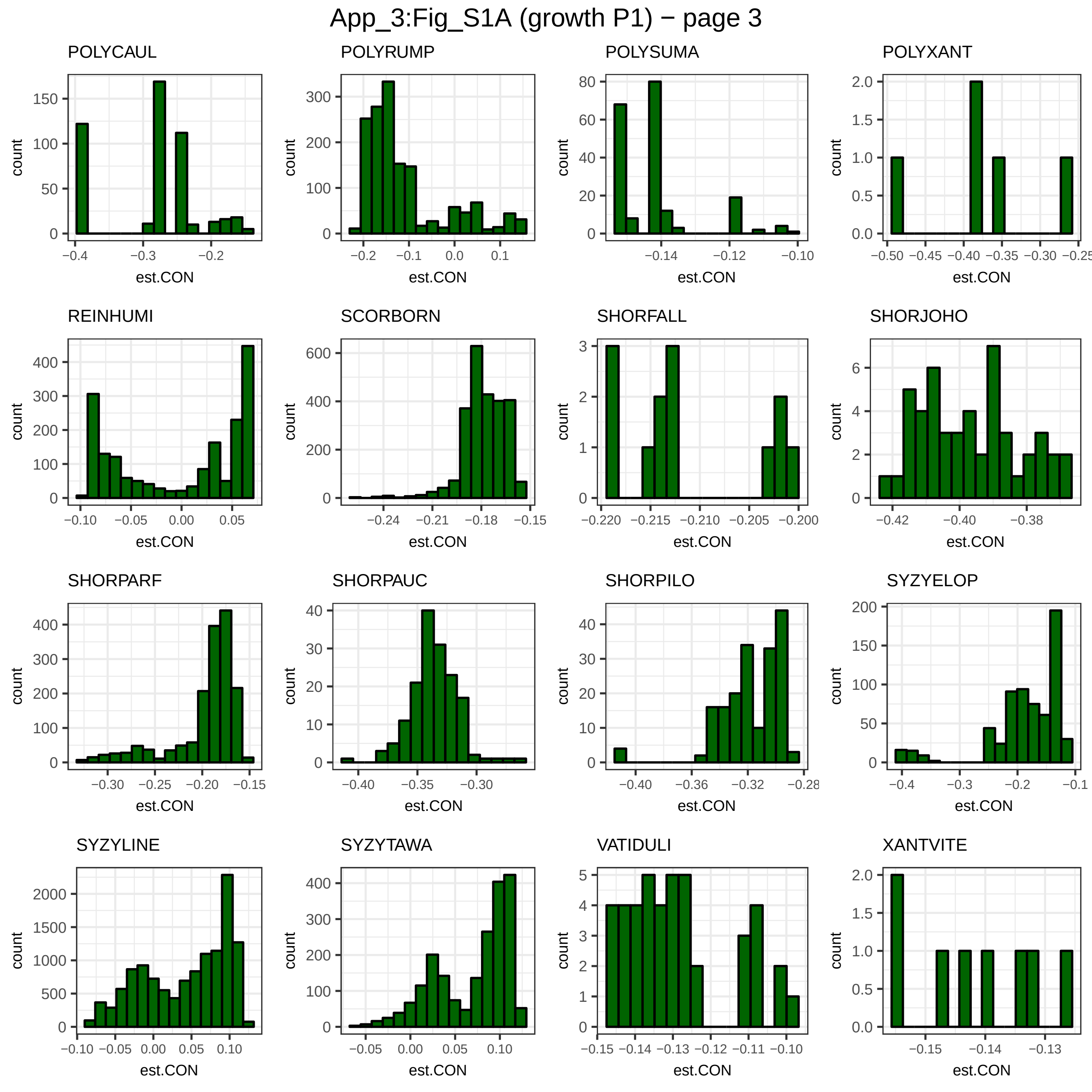
App_3:Fig_S1A (growth P1) − page 3
POLYCAUL
POLYRUMP
POLYSUMA
POLYXANT
REINHUMI
SCORBORN
SHORFALL
SHORJOHO
SHORPARF
SHORPAUC
SHORPILO
SYZYELOP
SYZYLINE
SYZYTAWA
VATIDULI
XANTVITE
count
est.CON

App_3:Fig_S1B (growth P2) − page 1

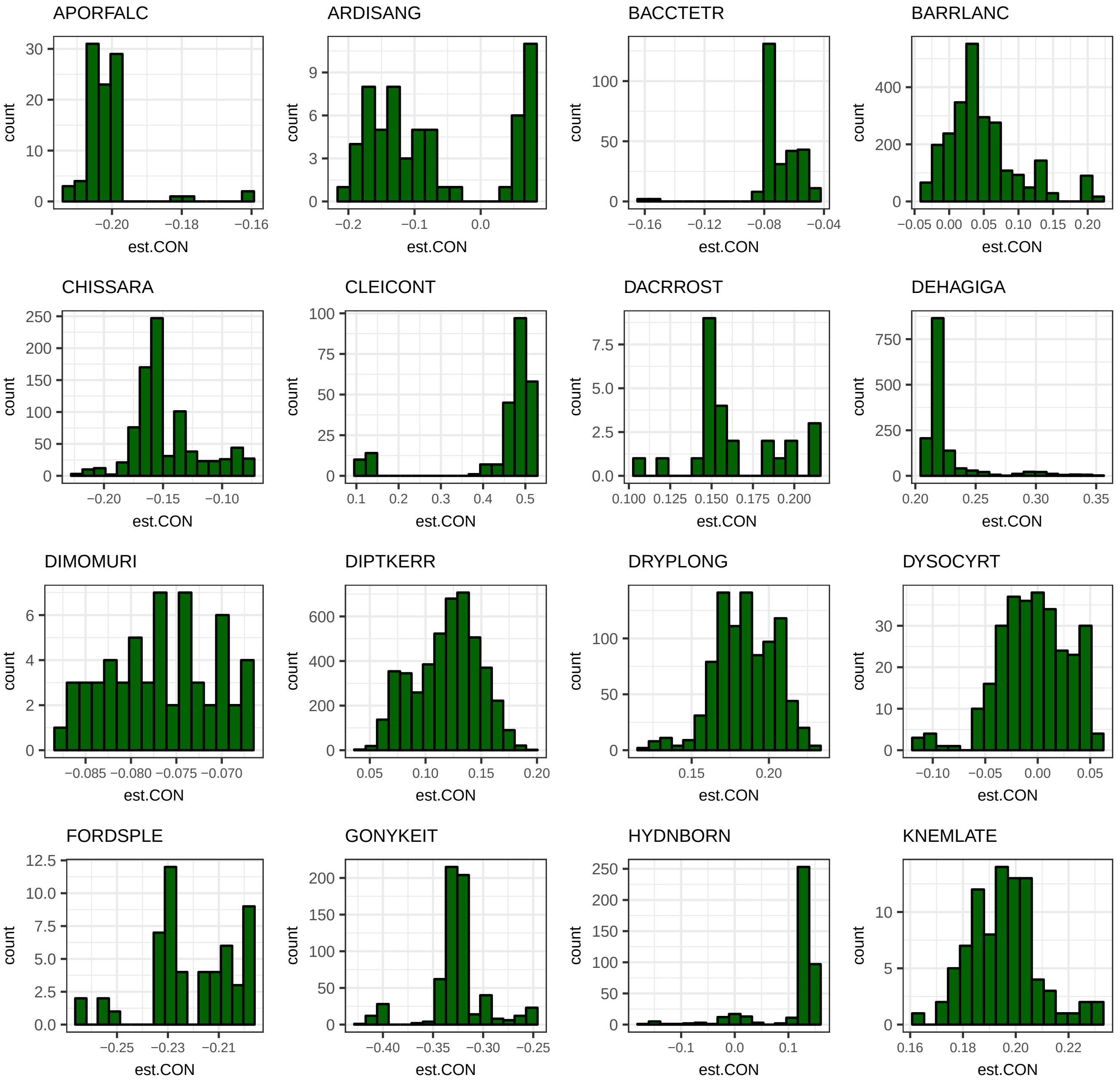

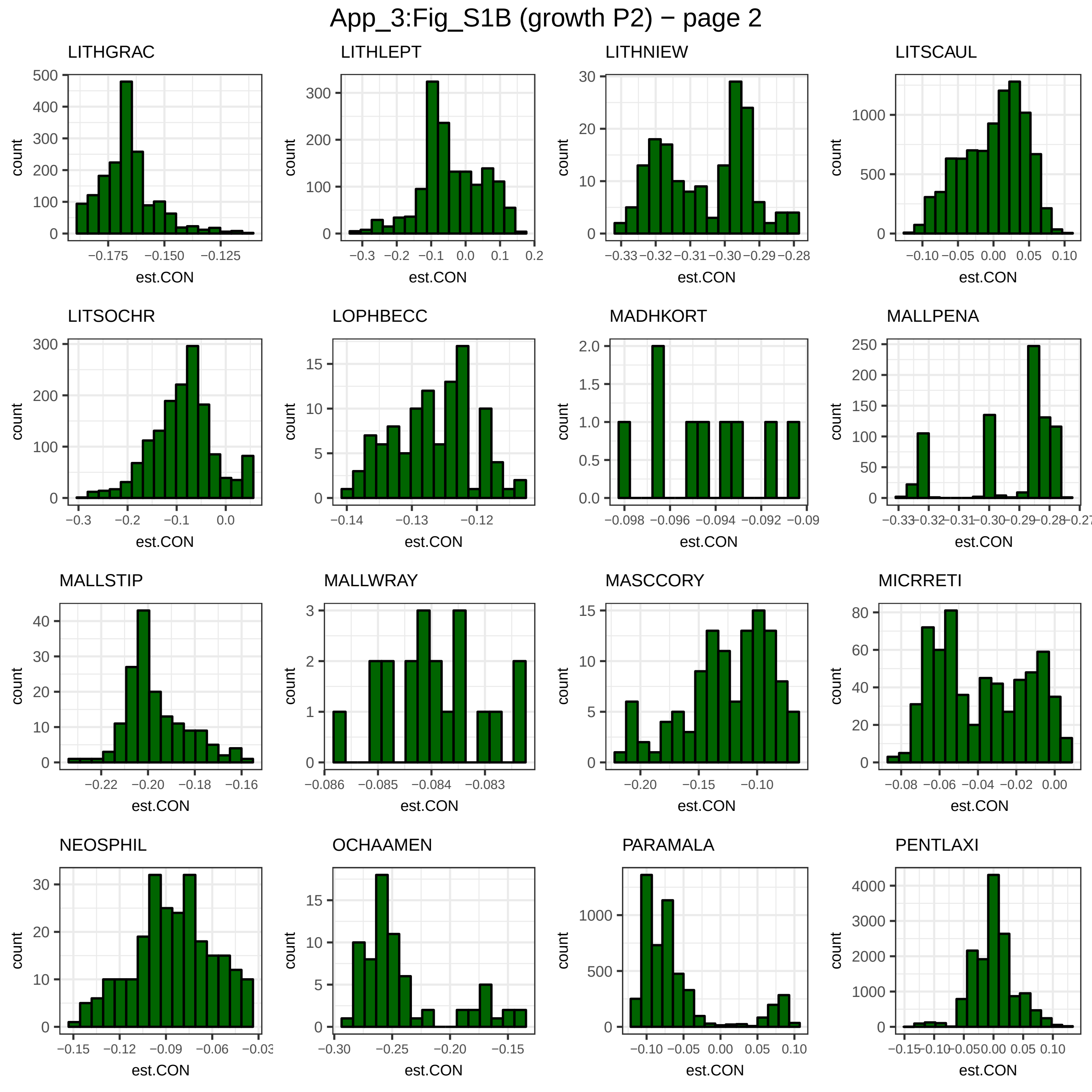
App_3:Fig_S1B (growth P2) − page 2
LITHGRAC
LITHLEPT
LITHNIEW
LITSCAUL
LITSOCHR
LOPHBECC
MADHKORT
MALLPENA
MALLSTIP
MALLWRAY
MASCCORY
MICRRETI
NEOSPHIL
OCHAAMEN
PARAMALA
PENTLAXI
count
est.CON

App_3:Fig_S1B (growth P2) − page 3

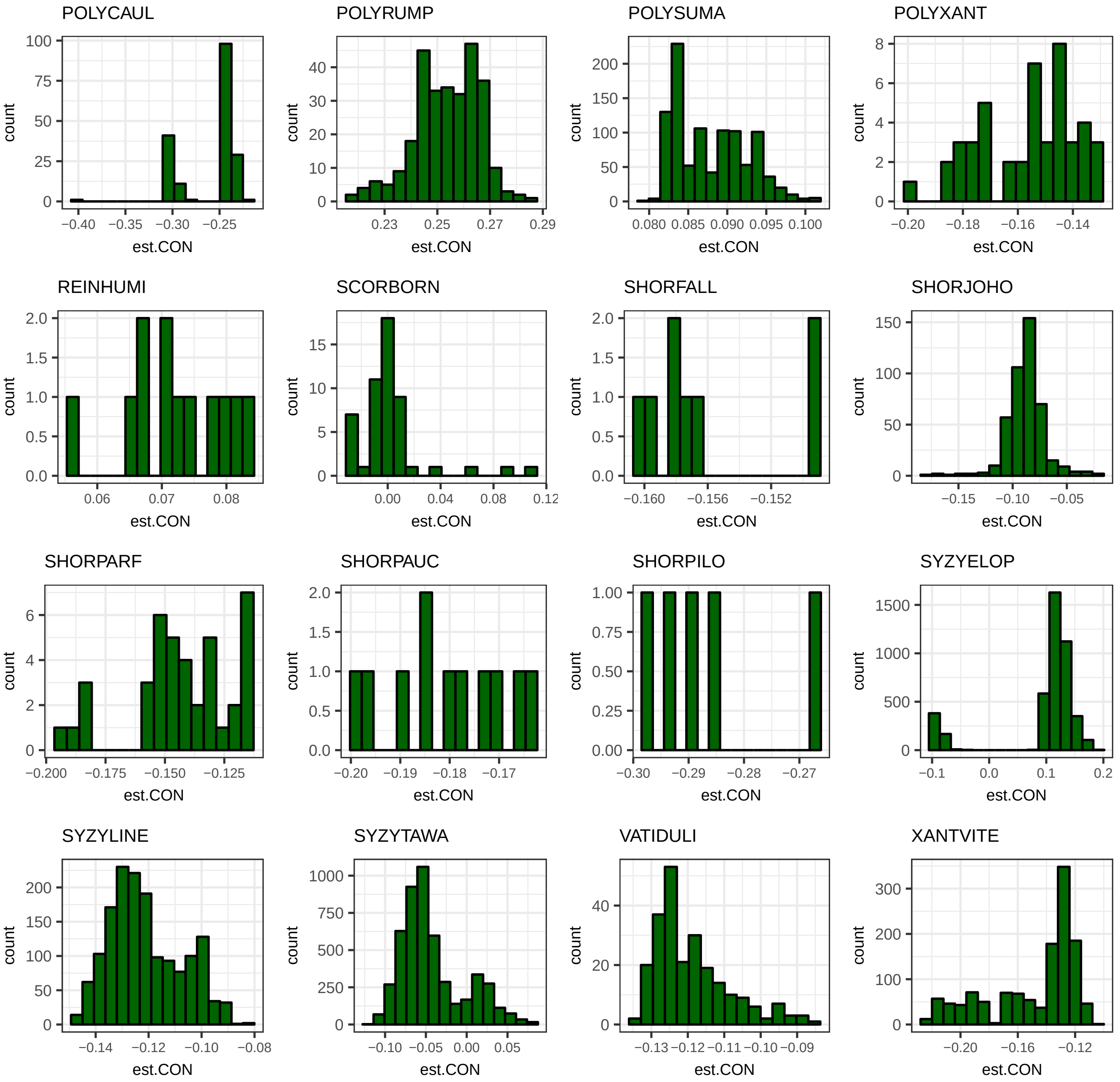

App_3:Fig_S2A (survival P1) − page 1

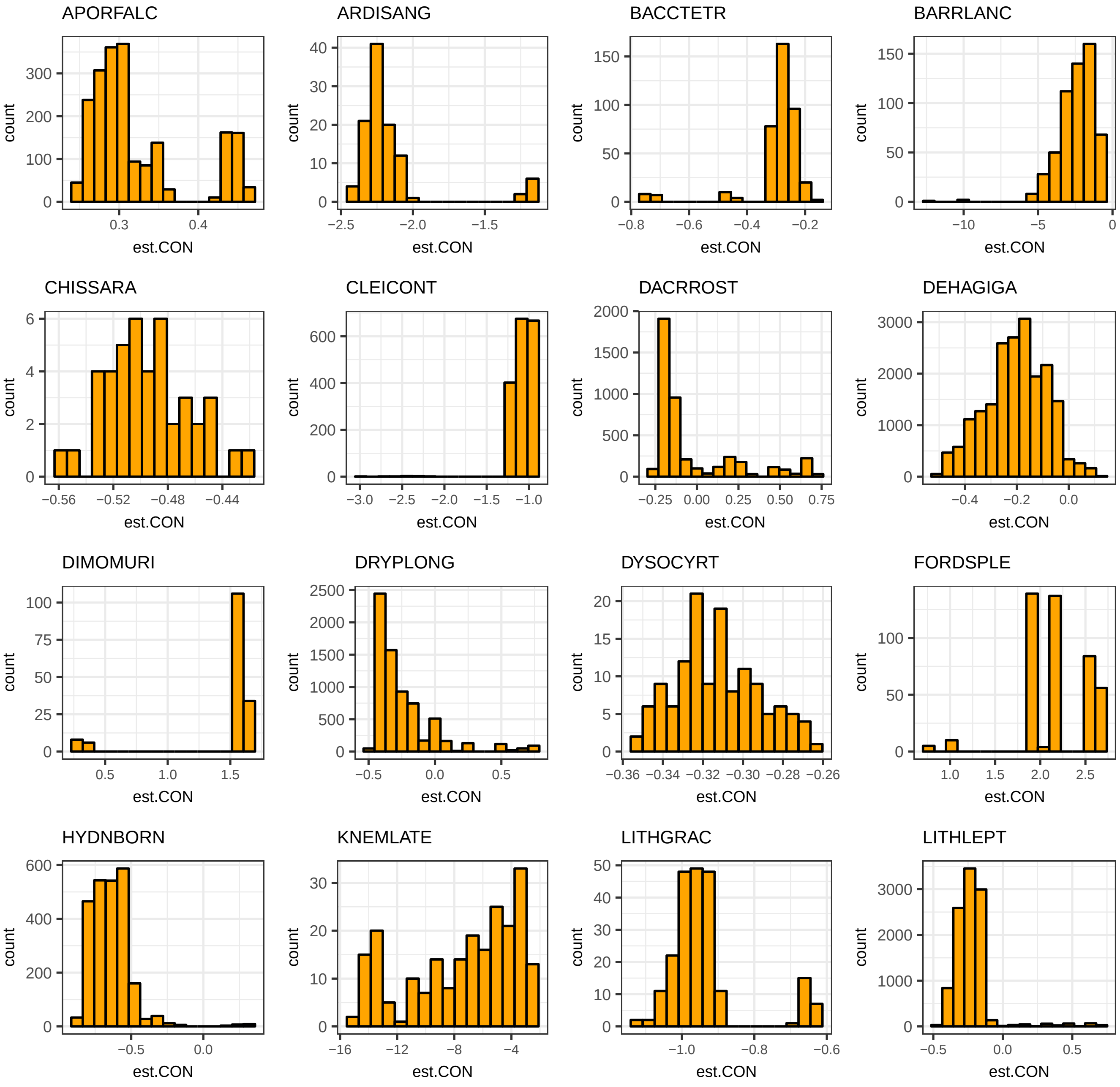

App_3:Fig_S2A (survival P1) − page 2

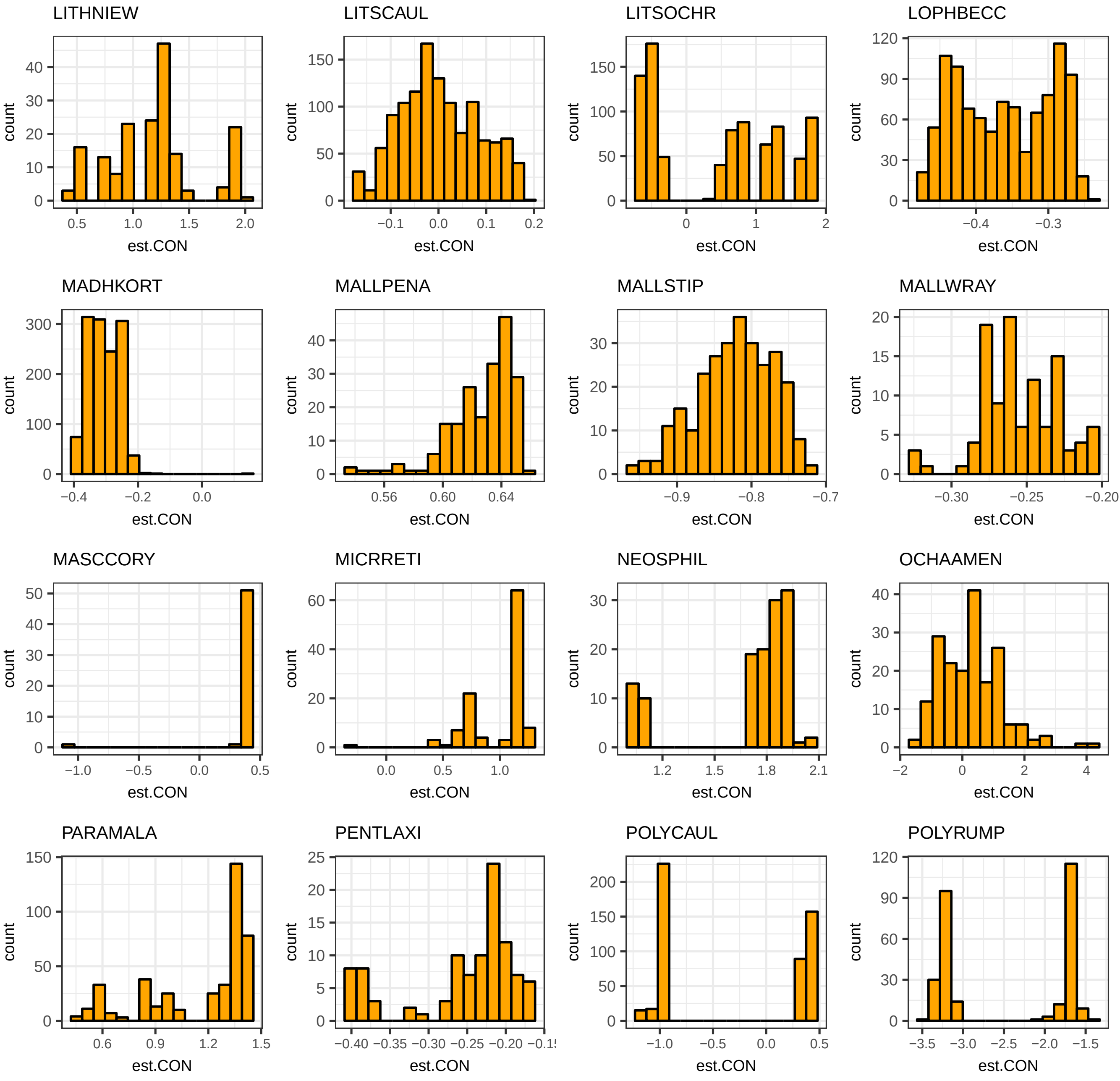

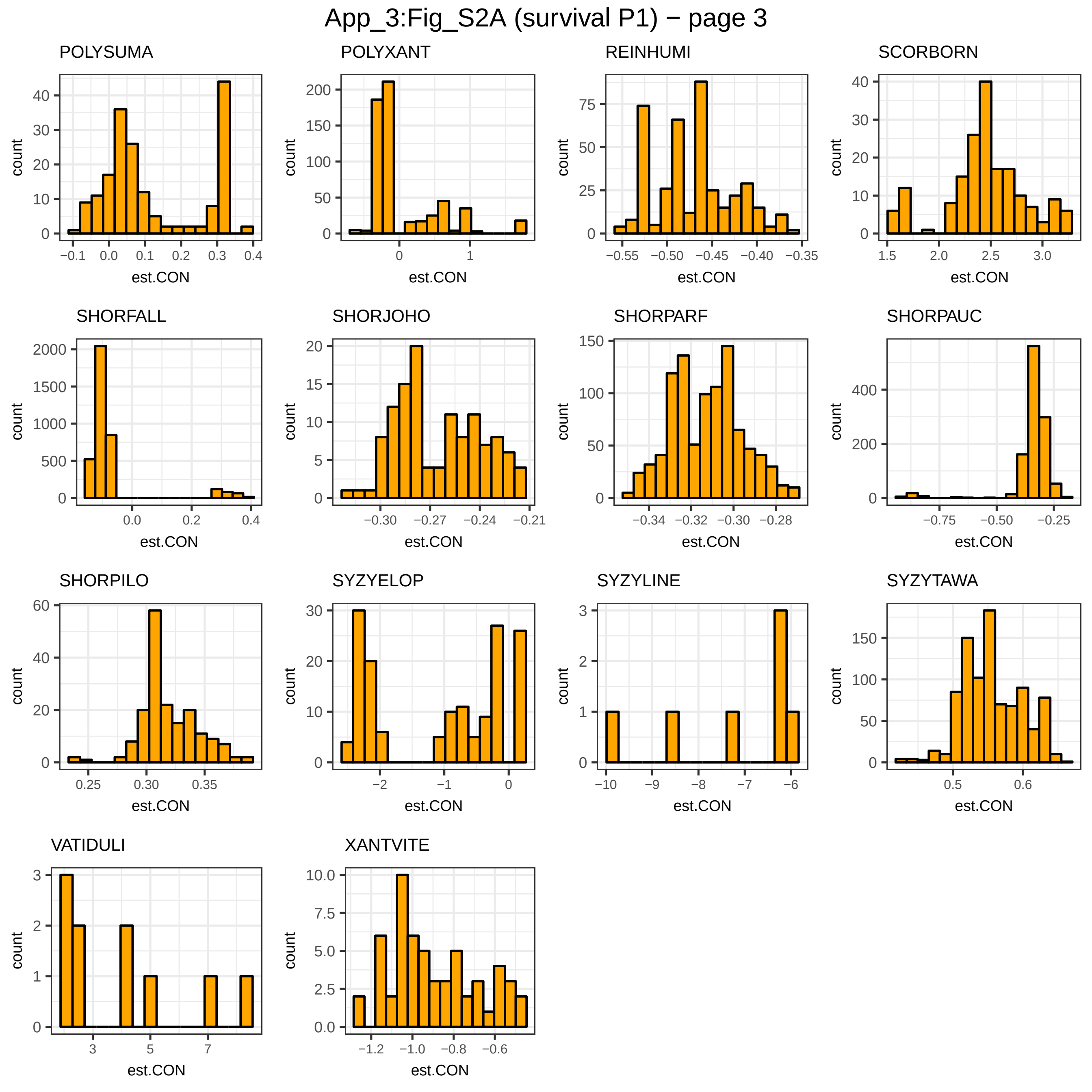
App_3:Fig_S2A (survival P1) − page 3
POLYSUMA
POLYXANT
REINHUMI
SCORBORN
SHORFALL
SHORJOHO
SHORPARF
SHORPAUC
SHORPILO
SYZYELOP
SYZYLINE
SYZYTAWA
VATIDULI
XANTVITE
count
est.CON

App_3:Fig_S2B (survival P2) − page 1

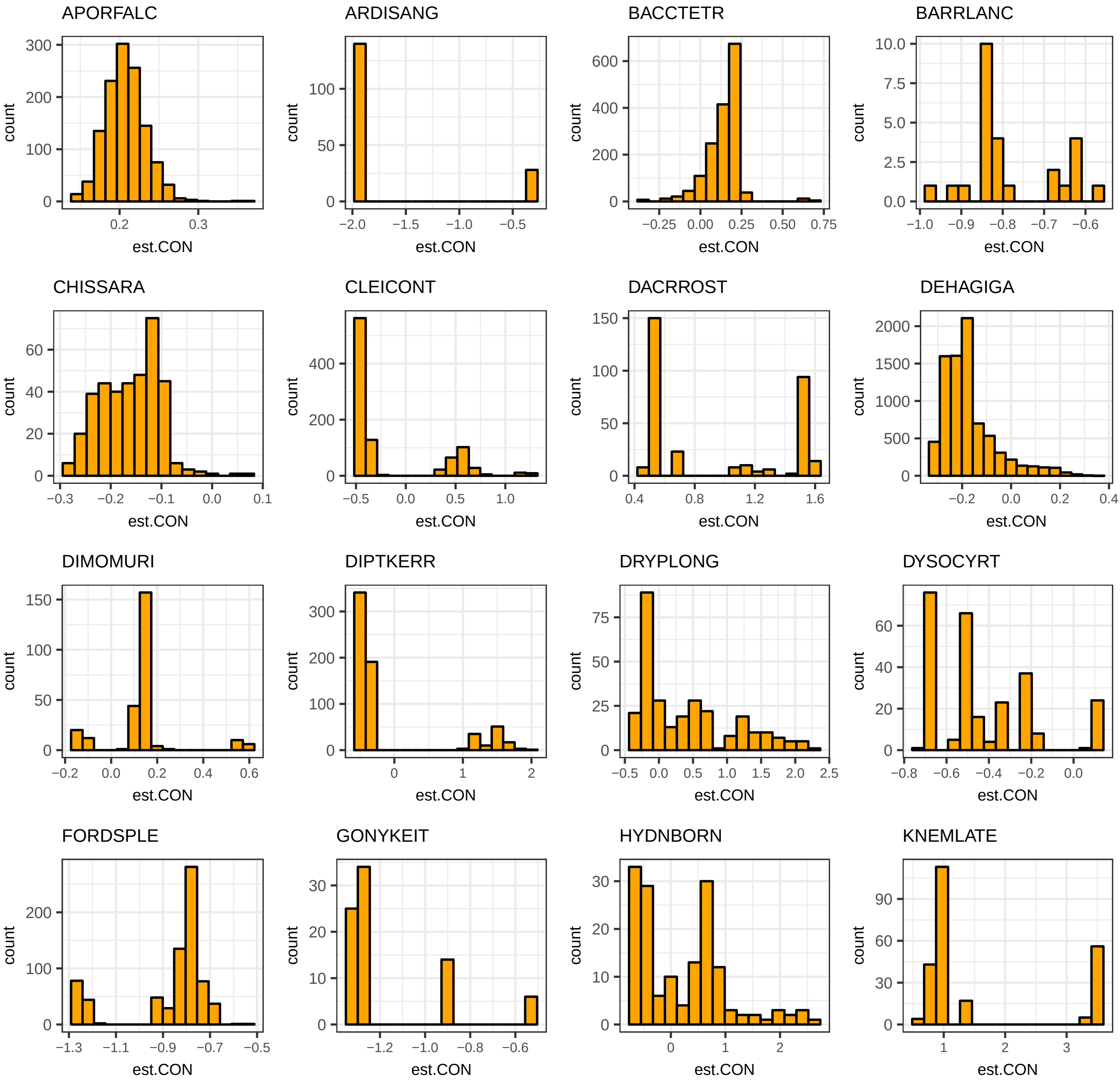

App_3:Fig_S2B (survival P2) − page 2

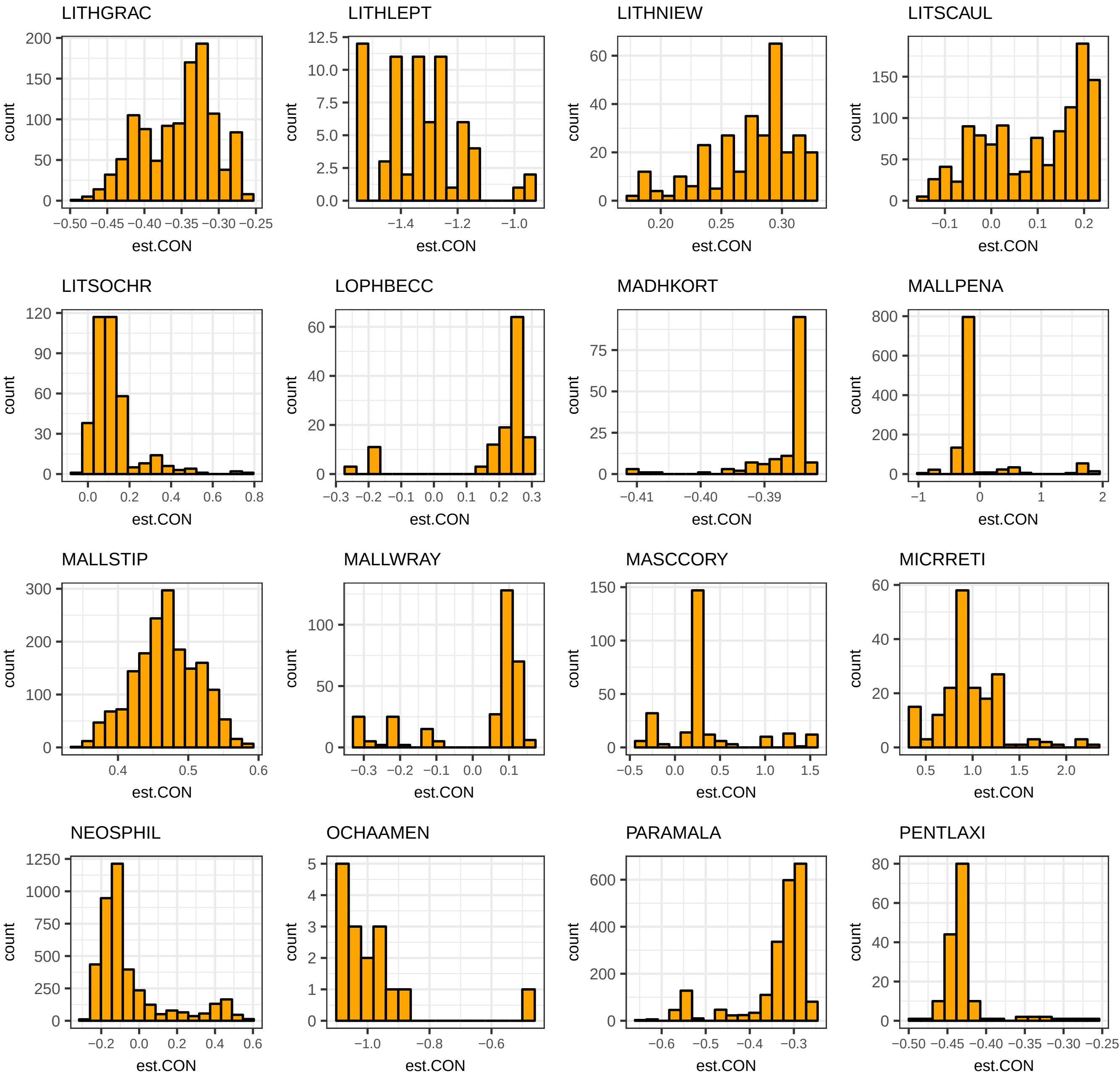

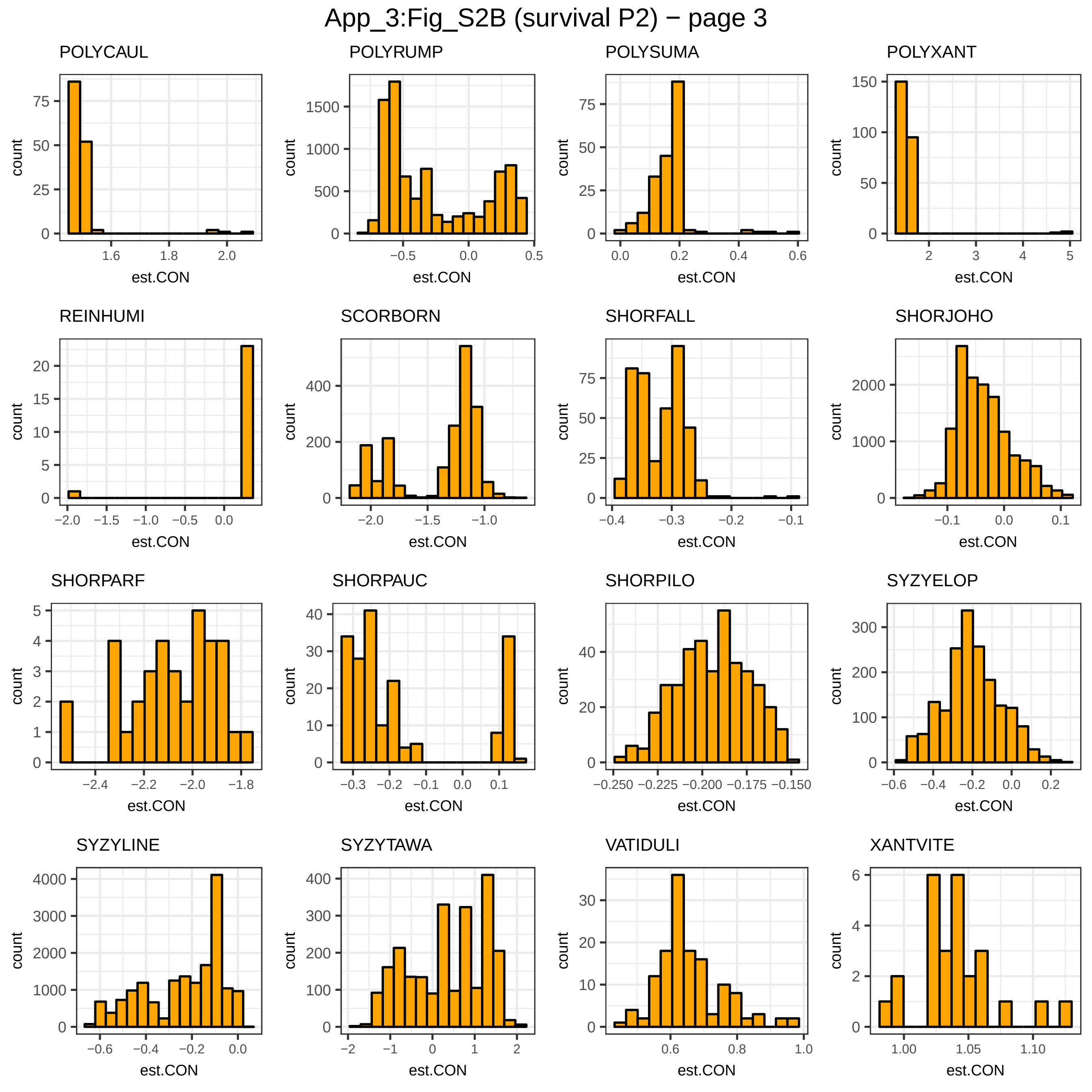
App_3:Fig_S2B (survival P2) − page 3
POLYCAUL
POLYRUMP
POLYSUMA
POLYXANT
REINHUMI
SCORBORN
SHORFALL
SHORJOHO
SHORPARF
SHORPAUC
SHORPILO
SYZYELOP
SYZYLINE
SYZYTAWA
VATIDULI
XANTVITE
count
est.CON

Appendix S4. Results of regression model fitting: non-spatial versus spatial.

Four tables of the coefficients, measures of statistical fit and significances, and best fitting radii, of the regression outcomes for all species, non-spatial and two ('removed' and 'relocated') spatial models, for growth and survival responses in periods 1 and 2 (Excel files).

One table of the effect sizes for CON and HET terms in the non-spatial and all eight combinations of spatial models, for all species, growth and survival responses in periods 1 and 2. (Excel file).

These multi-sheeted files are too large to reproduce in .pdf form as Supporting Information but are to be found at the Dryad Repository, with the raw data:

doi: 10.5061/dryad.q573n5tfx.

AppendixS4_GROWTH P1.xls
AppendixS4_GROWTH P2.xls
AppendixS4_SURVIVAL P1.xls
AppendixS4_SURVIVAL P2.xls

AppendixS4 Effect Sizes.xls

Appendix S5. Fitted CON coefficients versus corresponding best-fit radius for the 48 species neighbourhood (lin-decay) 'larsm/reloc/crown' models:

[Newbery/Stoll “Including tree spatial extension in the evaluation of neighborhood competition effects in Bornean rain forest”]

Fig. S1A growth in P_1
S1B growth in P_2
S2A survival in P_1
S2B survival in P_2

Number of points are fitted combinations of radius and Δd fits within the range of 2ΔAICc.

The five species referred to in the main text and footnote to Appendix S1: Fig. 1b are indicated with rad asterisks next to their names in Figure S2A.

App_5:Fig_S1A (growth P1) − page 1

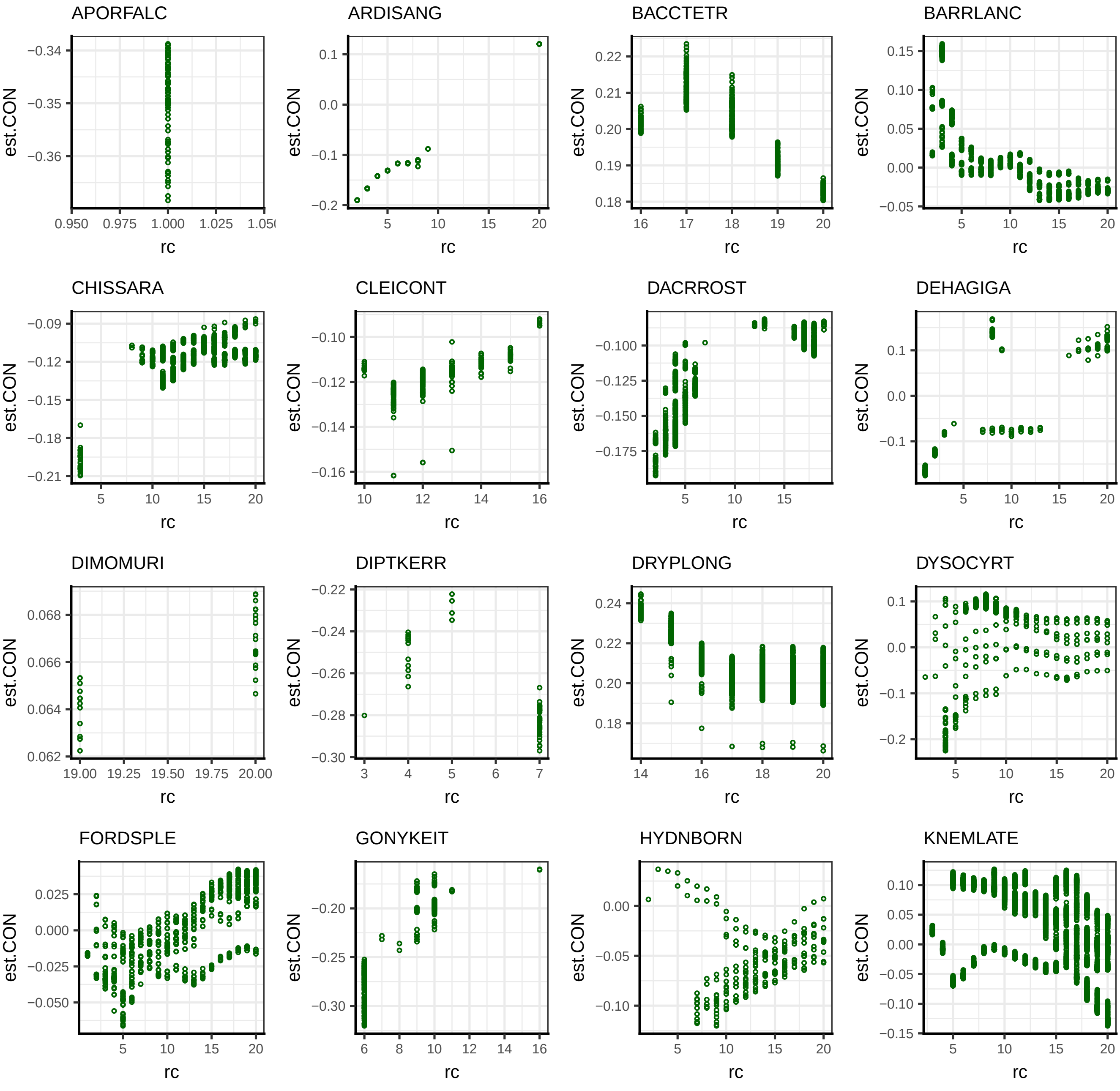

App_5:Fig_S1A (growth P1) − page 2

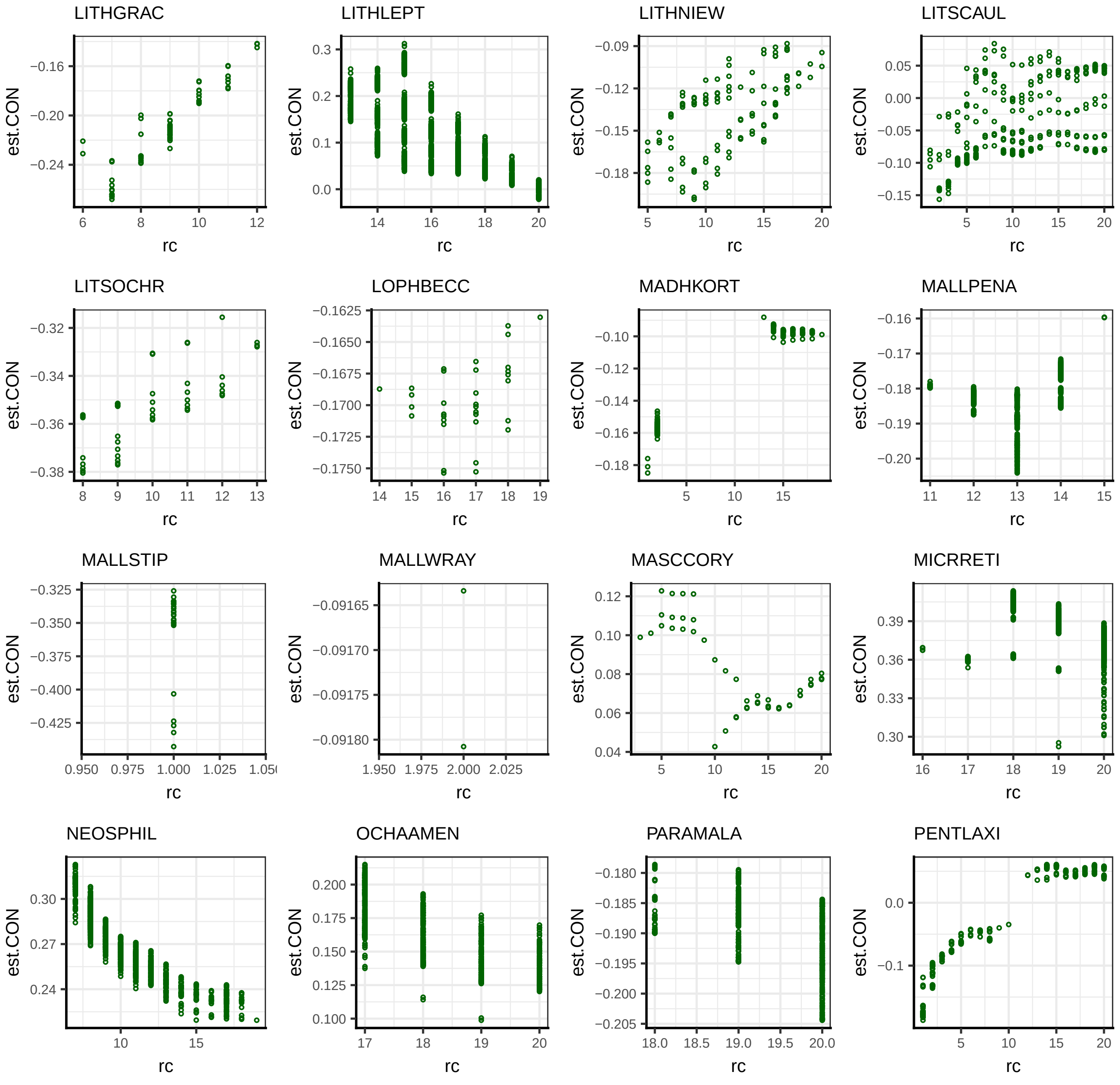

App_5:Fig_S1A (growth P1) − page 3

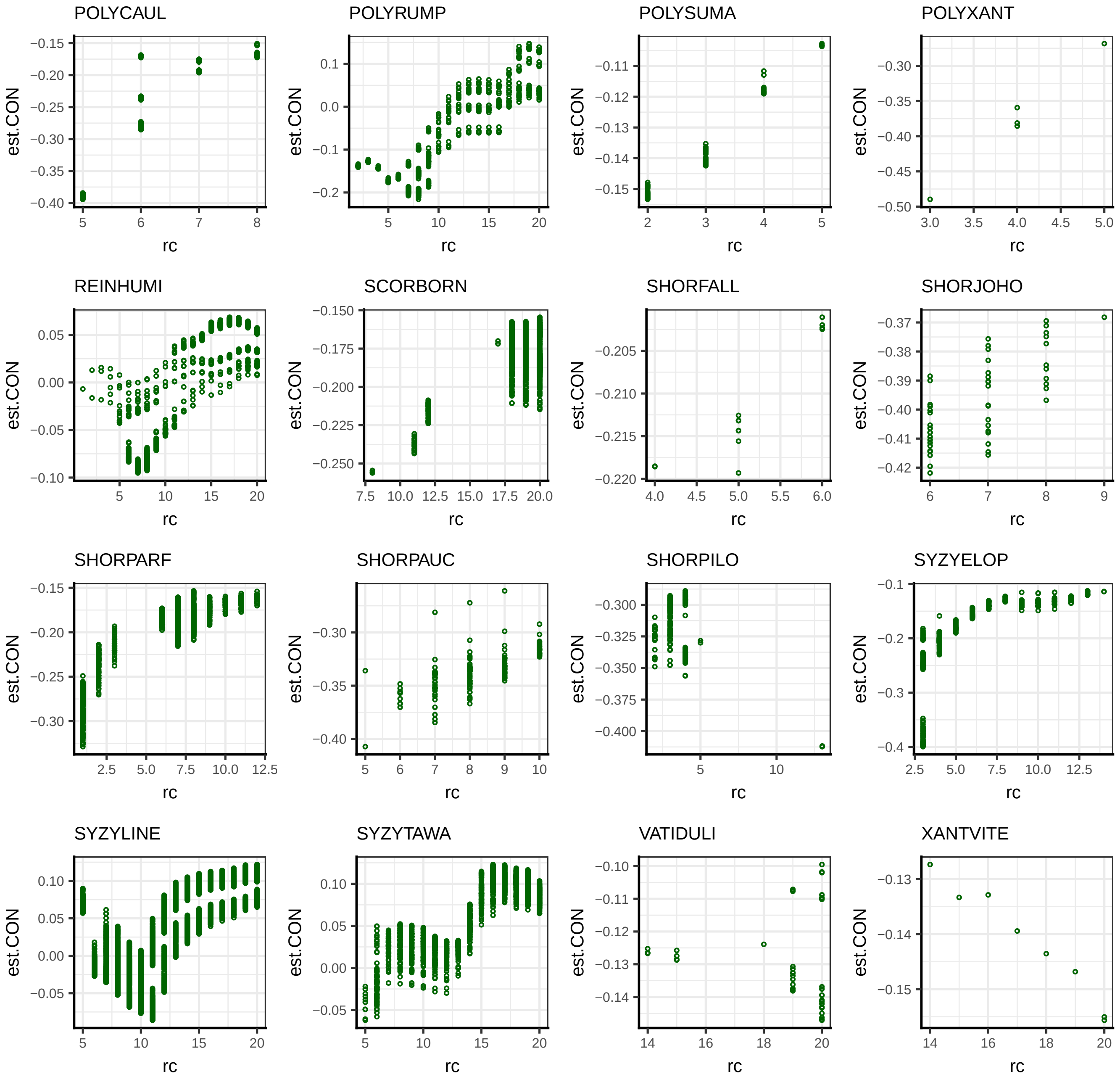

App_5:Fig_S1B (growth P2) − page 1

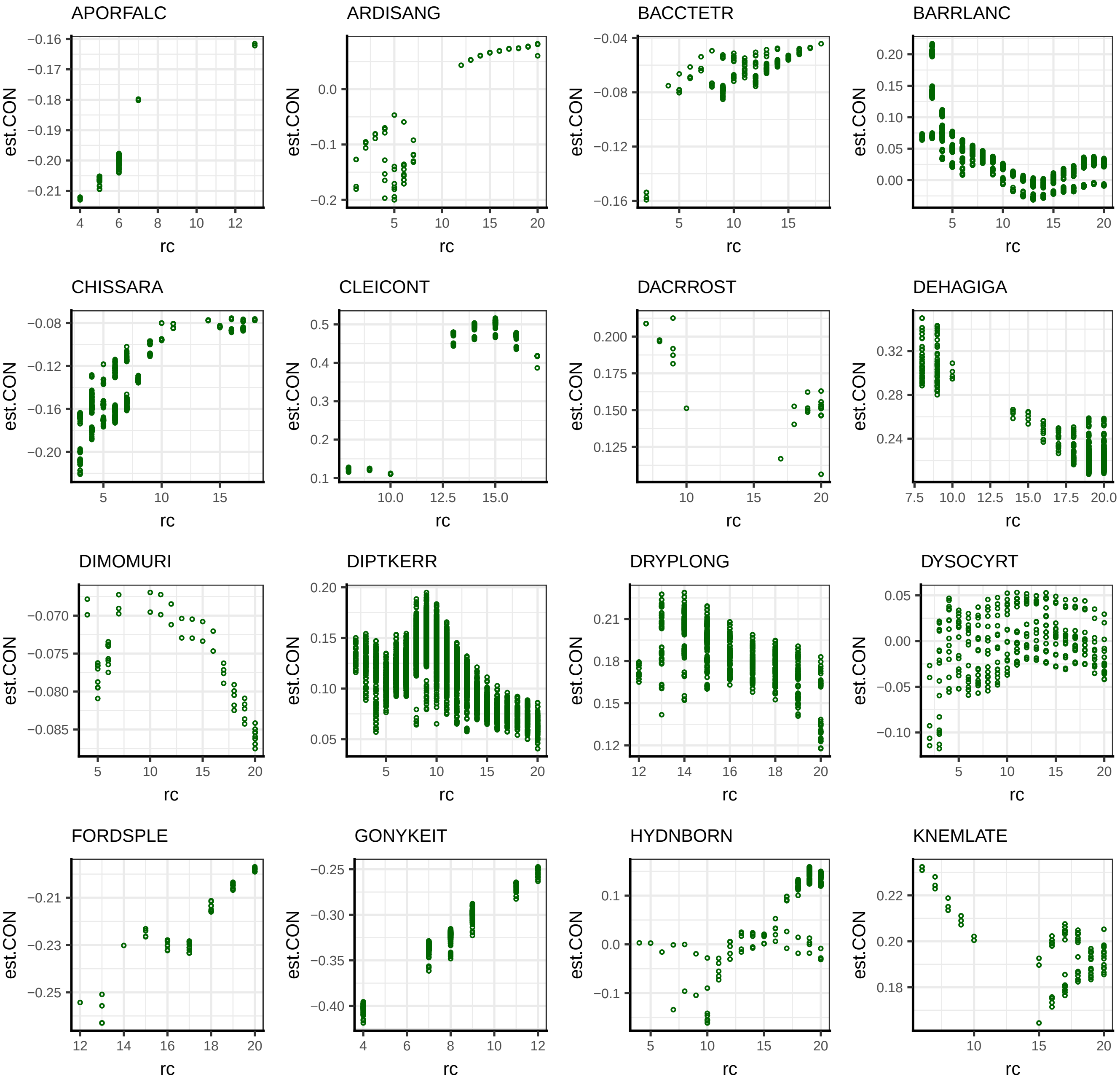

App_5:Fig_S1B (growth P2) − page 2

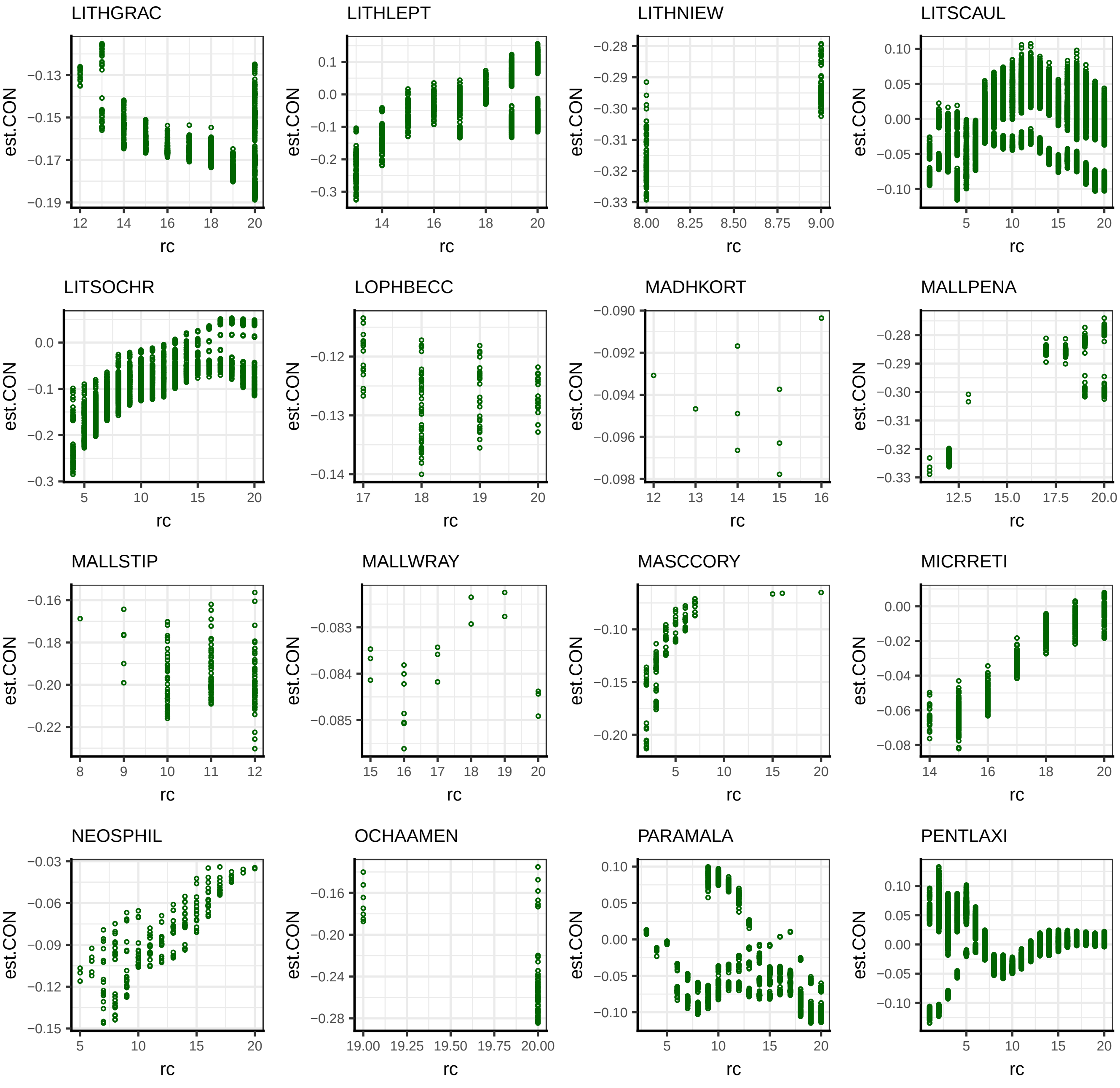

App_5:Fig_S1B (growth P2) − page 3

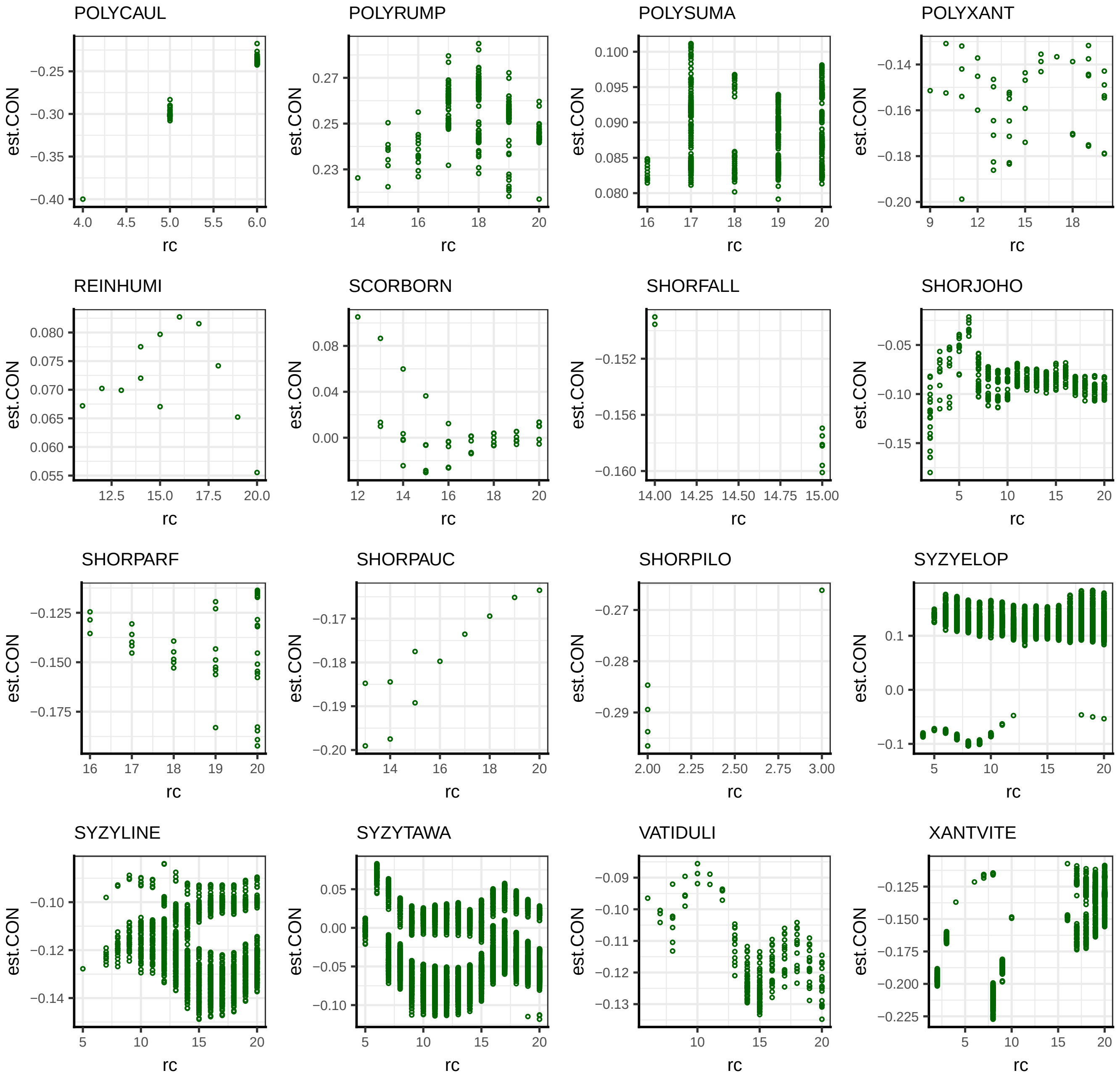

App_5:Fig_2A (survival P1) − page 1

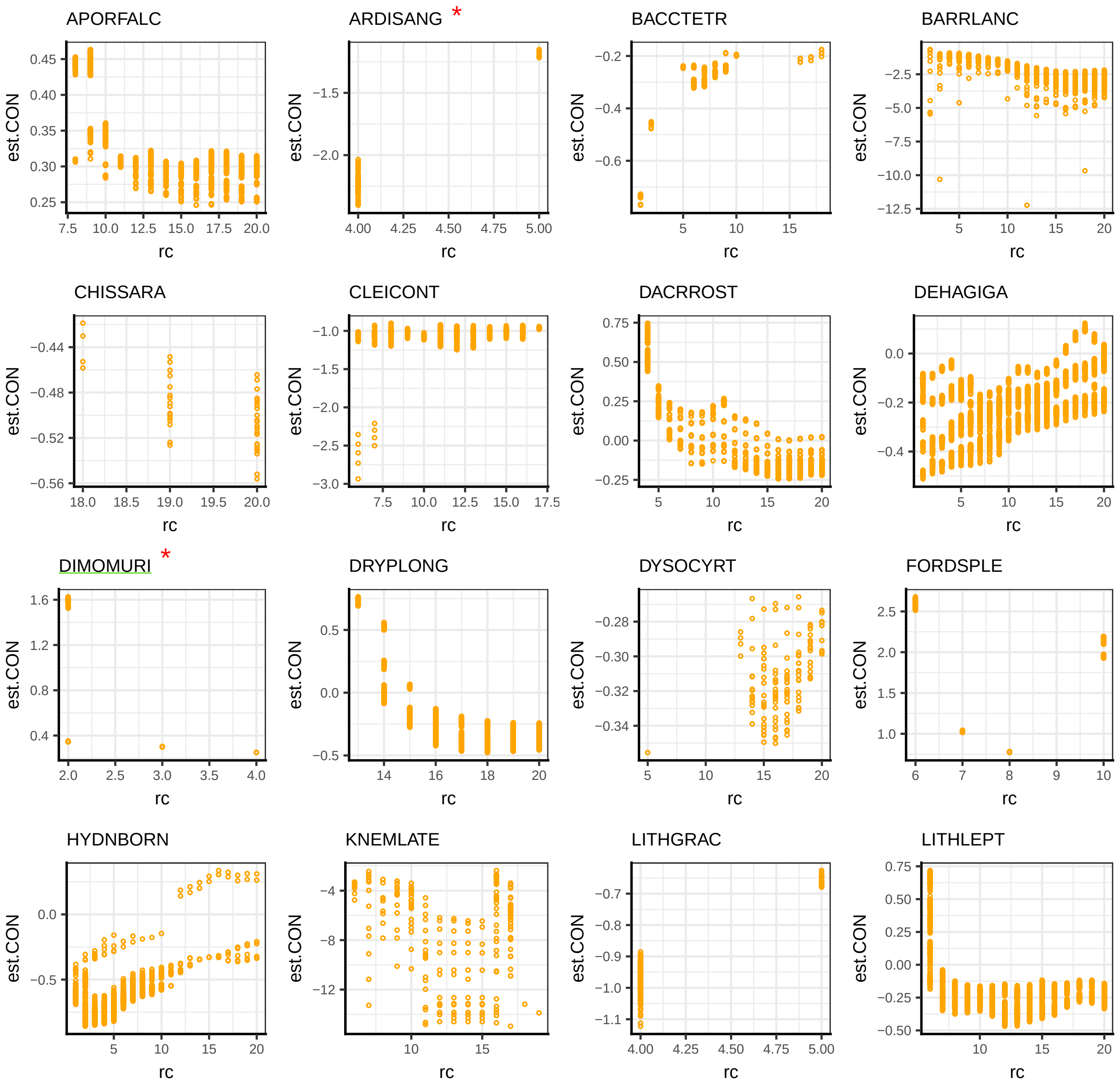

App_5:Fig_2A (survival P1) − page 2

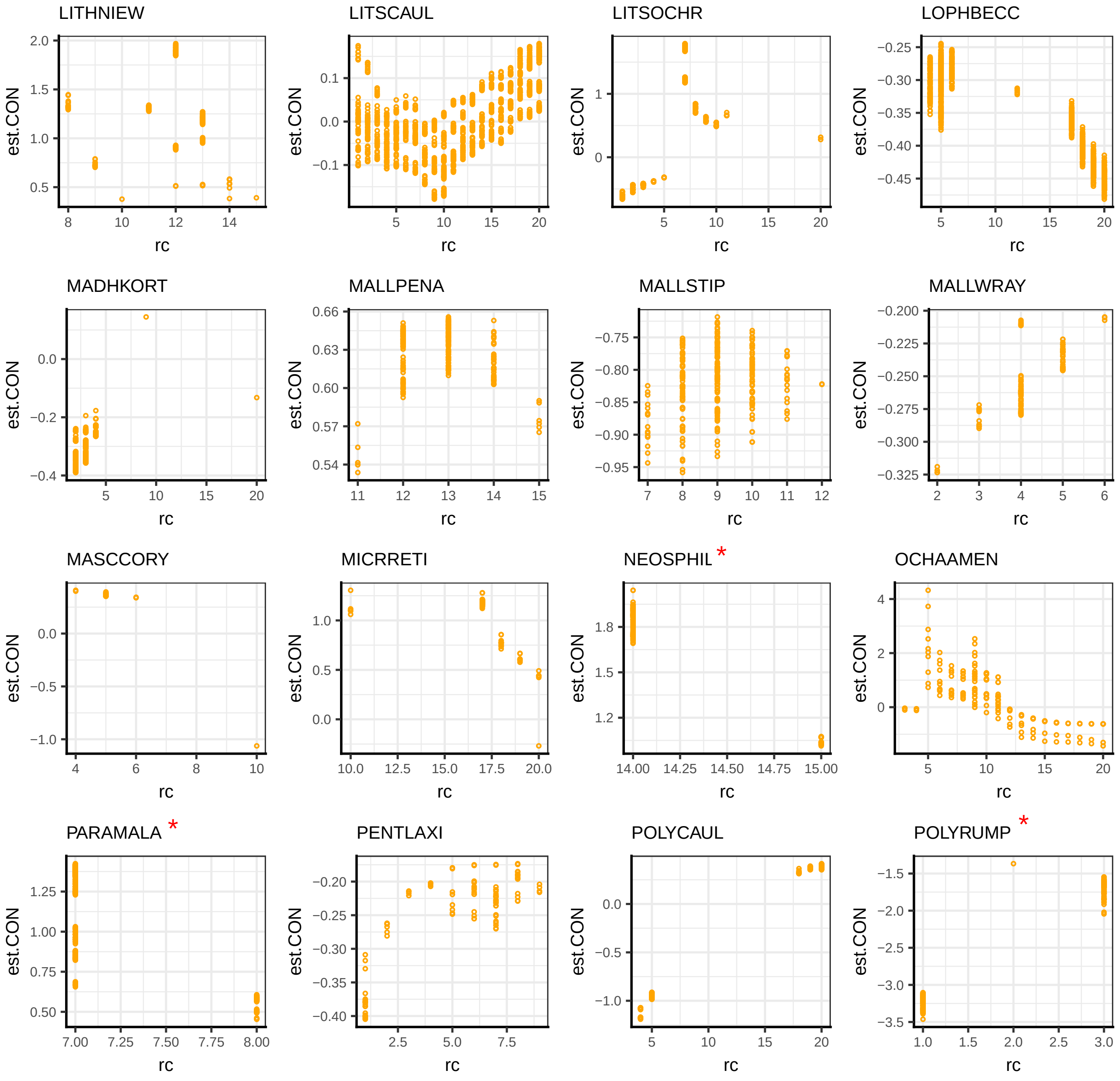

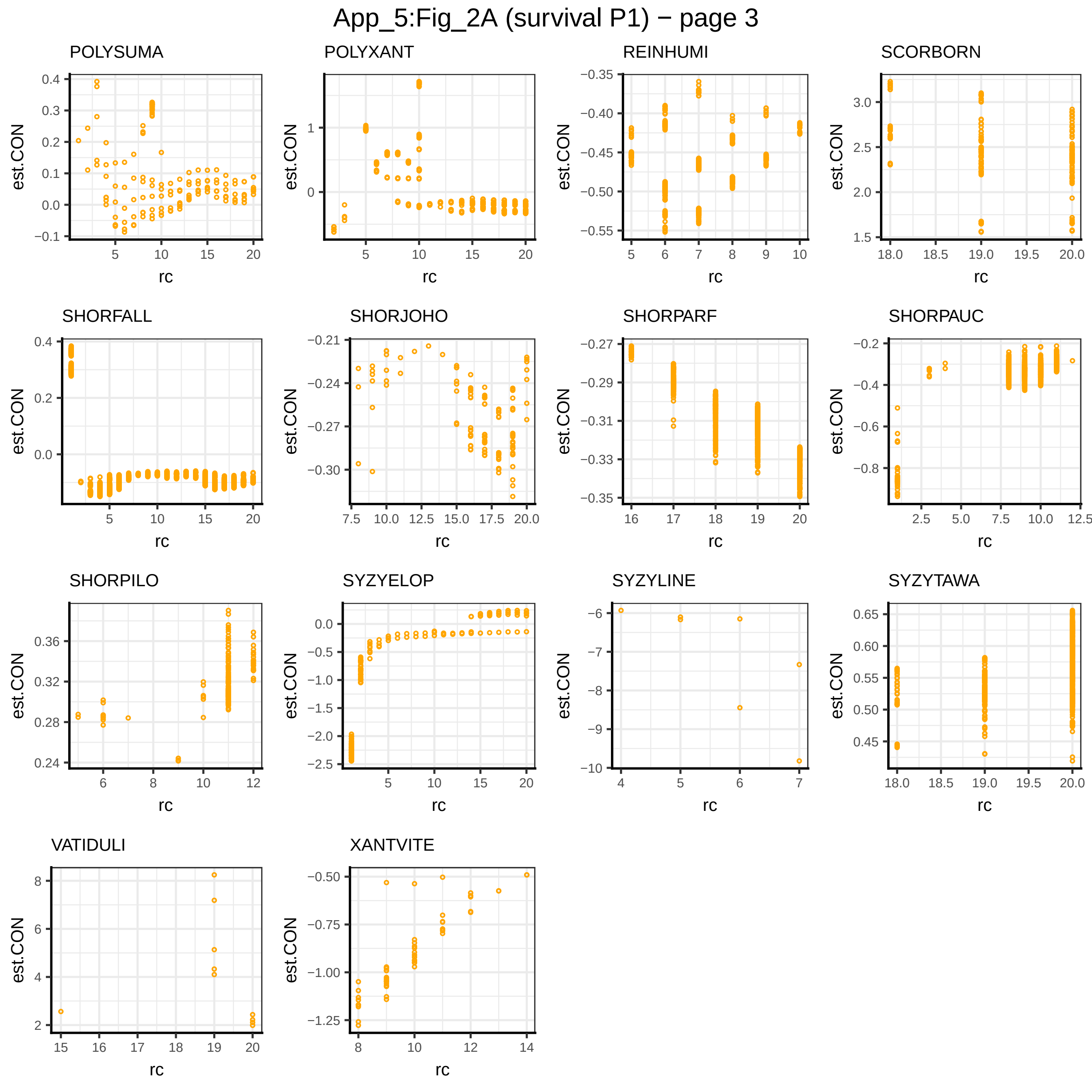
App_5:Fig_2A (survival P1) − page 3
POLYSUMA
POLYXANT
REINHUMI
SCORBORN
SHORFALL
SHORJOHO
SHORPARF
SHORPAUC
SHORPILO
SYZYELOP
SYZYLINE
SYZYTAWA
VATIDULI
XANTVITE
est.CON
rc

App_5:Fig_2B (survival P2) − page 1

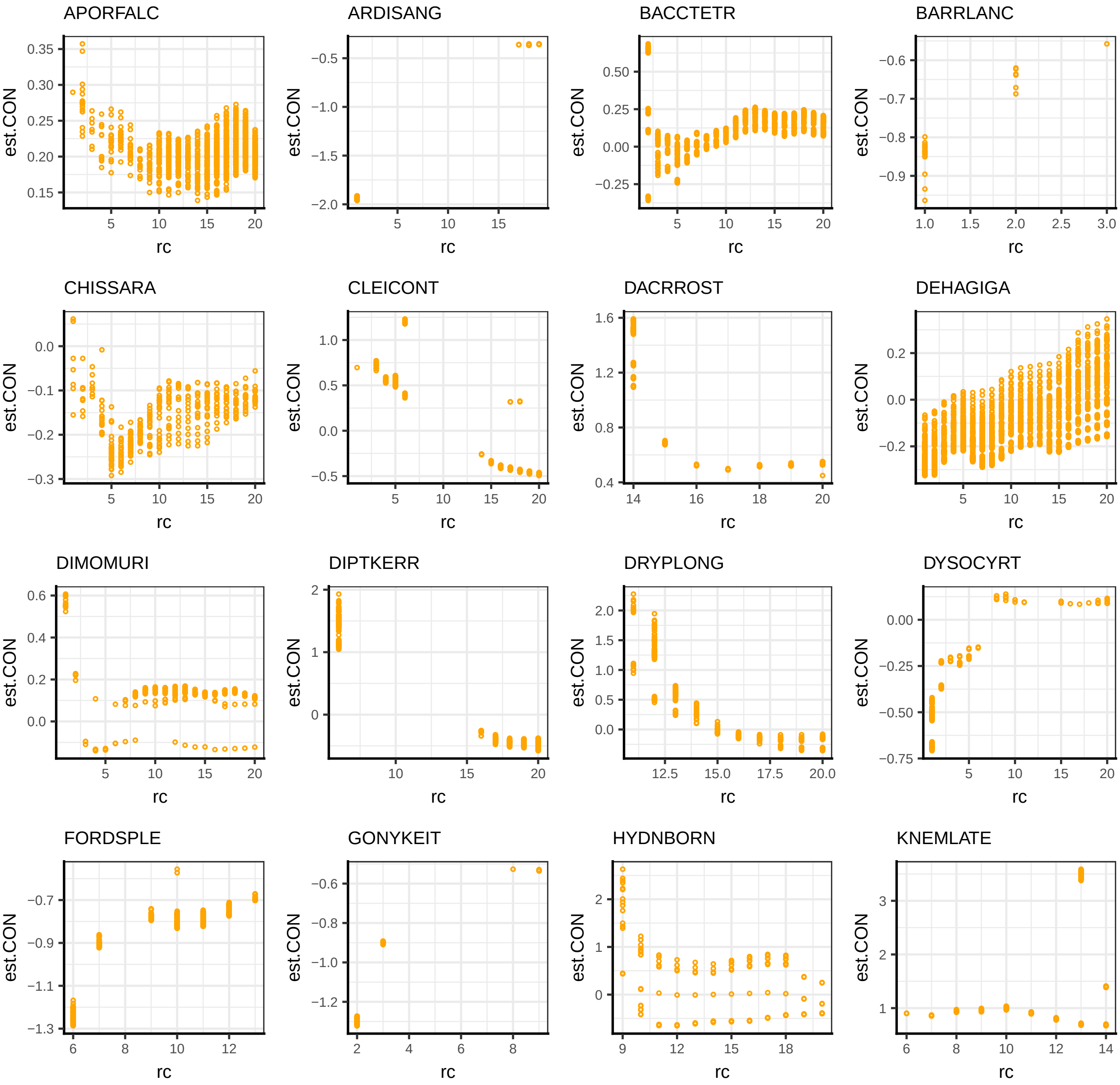

App_5:Fig_2B (survival P2) − page 2

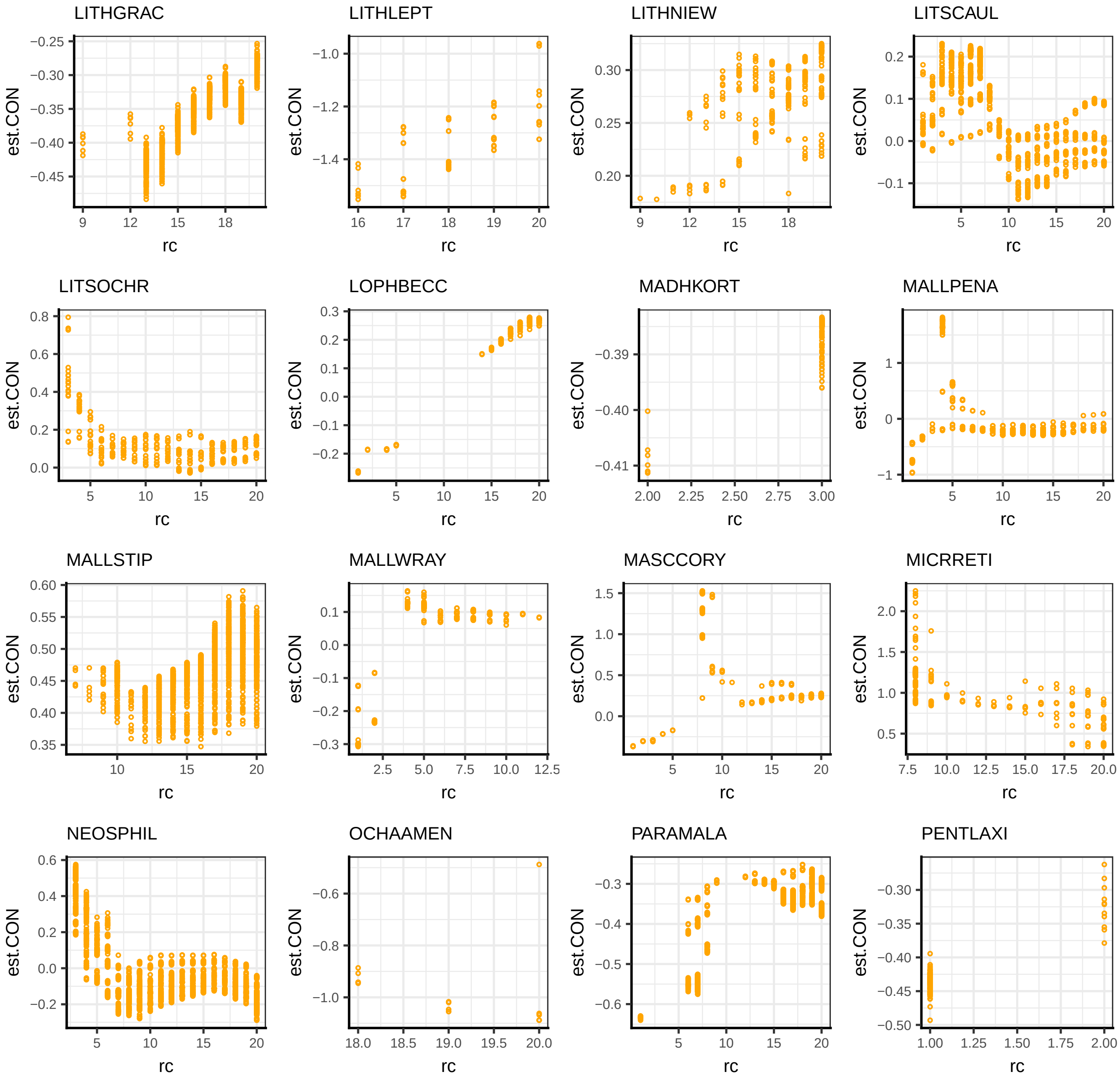

App_5:Fig_2B (survival P2) − page 3

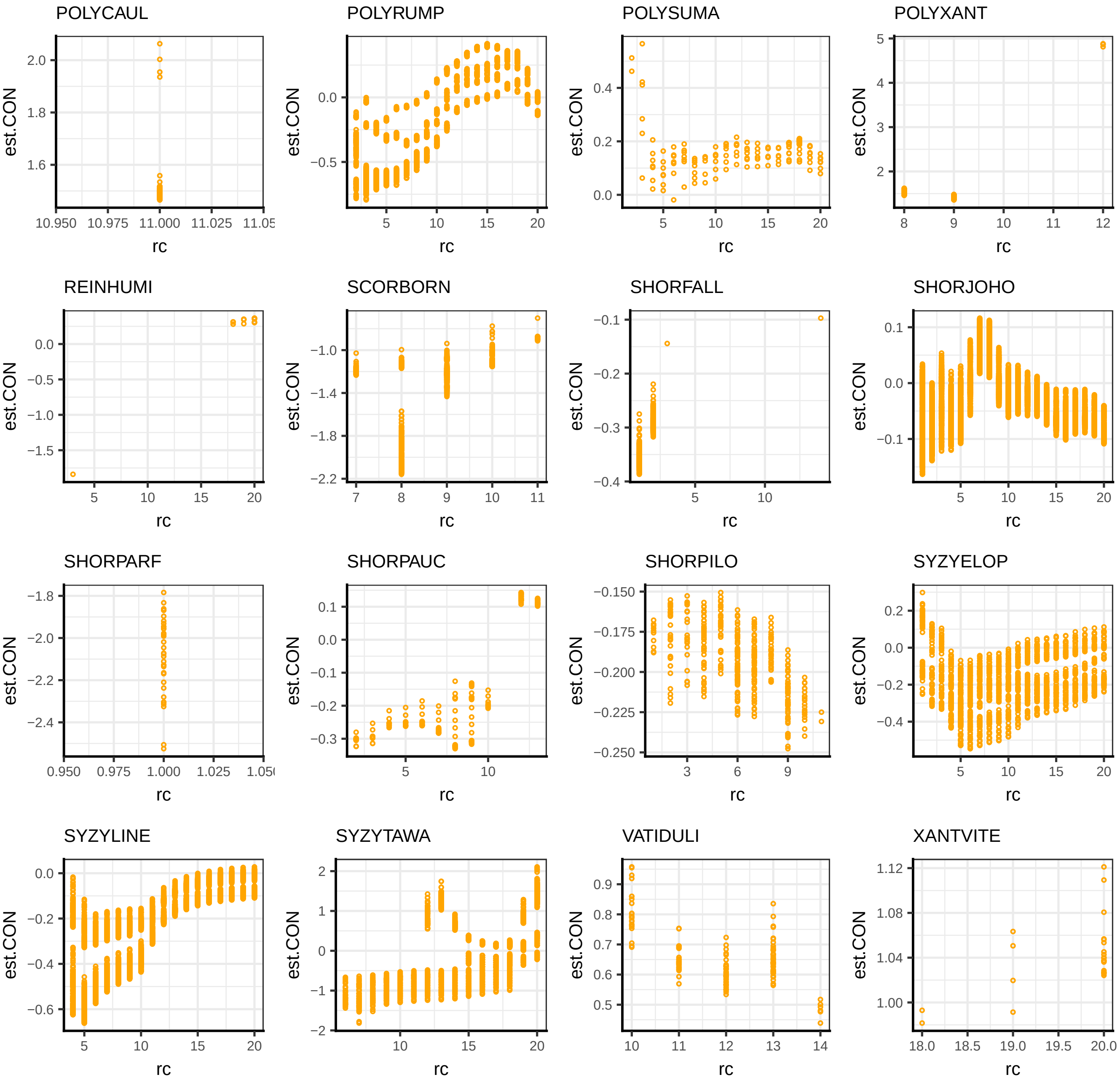

Appendix S6. Analysis of the role of structure, patterning and dynamics of the species studied in relation to CON and HET effect sizes

[Newbery/Stoll "Including tree spatial extension in the evaluation of neighborhood competition effects in Bornean rain forest"]

1. *Over-understorey structure*

The OUI was significantly negatively correlated with CON effects on growth in both P_1 and P_2 (r = −0.371, P = 0.022; r = −0.405, P = 0.012, respectively) and likewise for HET effects on growth (r = −0.388, P = 0.016; r = −0.432, P = 0.007). However, difference in CON and HET effects P_2−P_1 showed no correlation (r = 0.038 and 0.034, P > 0.8). By contrast CON effects on survival in P_1 and P_2 were positive yet insignificant or negatively weaker (r = 0.164, P = 0.33; r = −0.305, P = 0.063, respectively) with HET effects on survival slightly positive in P_1 but not P_2 (r = 0.281, P = 0.087; r = −0.249, P = 0.13). The means of the OUI-classes are shown in Table S1; Figs 2-4, main text, show the individual values color-coded to match the lines in Appendix 1: Table S1. The patterns of species' points in the community-level graphs (Figs 2b and 3b, main text, especially) are not therefore accountable by over-understorey structure: differencing CON effects on growth between periods almost completely removed any influence. It is, furthermore, noticeable that CON and HET effects on survival in P_1 were, on average, much more positive for the over-storey species and more negative for the under- and intermediate storeys, and this difference was reversed in P_2.

The correlations between difference in CON effect on growth P_2−P_1 versus CON or HET effect on survival when separated into the OUI classes were all insignificant (under-, intermediate, over-storey: CON −0.516, −0.113, −0.465; HET −0.448, −0.086, −0.512, respectively; range of P = 0.059 to 0.78)).

Appendix S6: Table S1. Mean values of the 38 selected species' effect sizes for the three over- understorey index (OUI) classes for the eight combinations of conspecific and heterospecific effect size (CON, HET), growth and survival variables (G, S), and for periods 1 and 2 (P_1, P_2). OUI classes: under-, < 20; intermediate, ≥ 20 – 55; and over-storey, > 55 (scale of 0 – 100). The classes are correspondingly color-coded in Figs 2-5, main text, and in Appendix 1: Table S1.

		OUI:	under-	intermediate	over-
		n	14	13	11
CON	G	P_1	−0.026	−0.058	−0.104
		P_2	−0.017	−0.029	−0.091
CON	S	P_1	−0.057	−0.450	0.235
		P_2	0.092	0.101	−0.396
HET	G	P_1	−0.031	−0.055	−0.118
		P_2	−0.028	−0.035	−0.081
HET	S	P_1	−0.210	−0.270	0.505
		P_2	0.363	0.131	−1.056

2. *Population dynamics*

Mortality, recruitment and stem relative growth rates, *rgr*, of small trees (10 - < 50 cm *gbh*) for the 38 selected species (D. M. Newbery and others, unpublished data) in P_1 were all strongly negatively correlated ($P \leq 0.01$) with CON effects on growth in that same period, but only *rgr* was similarly negatively correlated with CON effects on growth in P_2 (Table S2). Mortality for P_1 and P_2, and *rgr* just for $P_{2,}$ were also negatively correlated ($P \leq 0.05$) with HET effects on growth in the same periods. These growth rates were limited to ones that were valid, however. The negative correlations between *rgr* and CON effects on growth in P_1 were reaffirmed in more limited comparisons involving 22 and 32 species out of the 38, from two previously published analyses for the same plots, and also for P_{2a} (the first part of P_2, 1996-1999; [19, 20; main text]), the short span that included the 1998 ENSO drought, yet not in the P_{2b} following (1999-2001). With one exception, other variables were not significantly correlated; and all correlations between these variables small-tree population variables and CON and HET effects on survival, and differences in CON and HET effects on growth, were non-significant. Reactivity measured the relative change in *rgr* between periods or part periods, for instance between P_{2a} to P_{2b} (as *rea*), or how the *rgr* in these part periods (as rea_1 and rea_2) differed from the average *rgr* of the periods before (P_1) and after (P_{2b}) the ENSO disturbance (see Table S2). Surprisingly, *rea* was just weakly positively correlated with CON and HET effects on growth in P_1, and not in P_2 — the period in which the ENSO disturbance itself occurred. A PCA first axis though the community-level graph of difference in CON effects on growth versus CON effects on survival (Fig. 2b, main text) was not significantly correlated with any of the dynamics variables or reactivity.

Appendix S6: Table S2**.** Correlations (Pearson's *r*) between Danum plots' population dynamics variables and CON and HET effects on growth in periods 1 and 2 (P_1, P_2) from the 'larsm/reloc/crown' spatial nearest-neighbour model: (a) annualized mortality (m_a) and recruitment (r_a) and mean relative stem growth rate (*rgr*) for small trees [10 -< 50 cm gbh] in the full periods for which the effects estimates corresponded (D. M. Newbery, M. Lingenfelder & C. E. Ridsdale unpubl. data); (b) m_a and *rgr*, in two size classes of small tree as shown, for respectively the 22 species analyzed by Lingenfelder and Newbery [19, main text], and 32 of the species matching those of the present study analyzed by Newbery et al. [20, main text], in period 1 and in the first two of three parts of period 2 [a, 1996-1999; b, 1999-2001; c, 2001-2007], and part-period dynamics correlated with their corresponding full-period estimates; and (c) reactivity, in terms of *rgr* (*rea*, partitioned into rea_1 and rea_2), as calculated in Newbery et al.[20, main text].

(a) period	effect	$\ln(m_a)$	$\ln(r_a)$	ln(*rgr*)
P_1	CON	−0.454**	−0.425**	−0.482**
	HET	−0.339*	-0.200^{ns}	-0.234^{ns}
P_2	CON	-0.236^{ns}	-0.133^{ns}	−0.420**
	HET	−0.340*	-0.284^{o}	−0.387*

(b)	size:	10 - < 50 cm		12.5 - < 50 cm
		$\ln(m_a)$	ln(*rgr*)	ln(*rgr*)
period	effect			
P_1	CON	0.228^{ns}	−0.438*	−0.364*
	HET	−0.496*	-0.373^{o}	-0.071^{ns}
P_{2a}	CON	0.046^{ns}	-0.192^{ns}	−0.384*
	HET	-0.098^{ns}	0.201^{ns}	-0.129^{ns}
P_{2b}	CON	0.331^{ns}	0.241^{ns}	-0.296^{o}
	HET	-0.024^{ns}	0.104^{ns}	-0.253^{ns}

(c) period	effect	rea^a	rea_1	rea_2
P_1	CON	0.389*	0.296^{o}	0.148^{ns}
	HET	0.414*	0.292^{ns}	0.182^{ns}
P_2	CON	0.195^{ns}	0.215^{ns}	0.005^{ns}
	HET	-0.170^{ns}	-0.261^{ns}	0.073^{ns}

**, $P \leq 0.01$; *, $P \leq 0.05$; ^{o}, $P \leq 0.10$, ^{ns} $P > 0.05$.

[a], $rea = (rgr_{2b} - rgr_{2a})/mrgr_{1,2c}$; $rea_1 = (mrgr_{1,2c} - rgr_{2a})/mrgr_{1,2c}$; $rea_2 = (rgr_{2b} - mrgr_{1,2c})/mrgr_{1,2c}$.

3. *Spatial pattern*

The second-order spatial statistic 'L(r) – r' (Ripley 1977) was calculated for each of the 38 selected species, for the four combinations of plots 1 and 2 times P_1 and P_2, range r = 0 to 60 m, and for either trees of focal size (10 - < 100 cm *gbh*) or all trees (≥ 10 cm *gbh*). Most species showed very slight departures from randomness towards aggregation, more so in the range r = 40 – 60 m ($P \leq 0.05$), but differing often between plots, not periods. Five of 20 extended understorey species (OUI ≤ 30) had very strong aggregations in both plots (*Cleistanthus contractus, Dimorphocalyx muricatus, Lophopetalum beccarianum, Mallotus penangensis, M. stipularis*; see Appendix 1: Table S1 for full names). CON and HET effects on growth, and on survival, in P_1 and P_2 (eight *t*-tests), showed no significant differences in their means between aggregated and non-aggregated species, however (P = 0.17 – 0.76). Among the 18 intermediate-overstorey species (OUI > 30), just two were clearly aggregated (*Shorea fallax, S. pilosa*), four others more weakly so and not always consistently so across both plots (*Lithocarpus gracilis, Pentace laxiflora, Parashorea malaanonan, Syzygium elopurae*). Among the dipterocarps, comparing the two strongly aggregated dipterocarp species (*S. fallax, S. pilosa*) with the four others weakly or non-aggregated (*P. malaanonan, S. johorensis, S. parvifolia, S. pauciflora*), CON and HET effects (again the eight *t*-tests), showed no significant differences in their means (P = 0.09 – 0.89). The more outlying species' points in Figs 2b and 3b (main text), showed no particular associations with degree of aggregation. Taken together these comparisons provide little support for spatial patterning explaining the community-level relationships.

Reference*:* Ripley BD. Modelling spatial patterns. Journal of the Royal Statistical Society B. 1977; 39:172-212.

Appendix S6: Figure S1. Spatial pattern analysis of the 38 species used in the nearest-neighbour modelling (on the following pages).

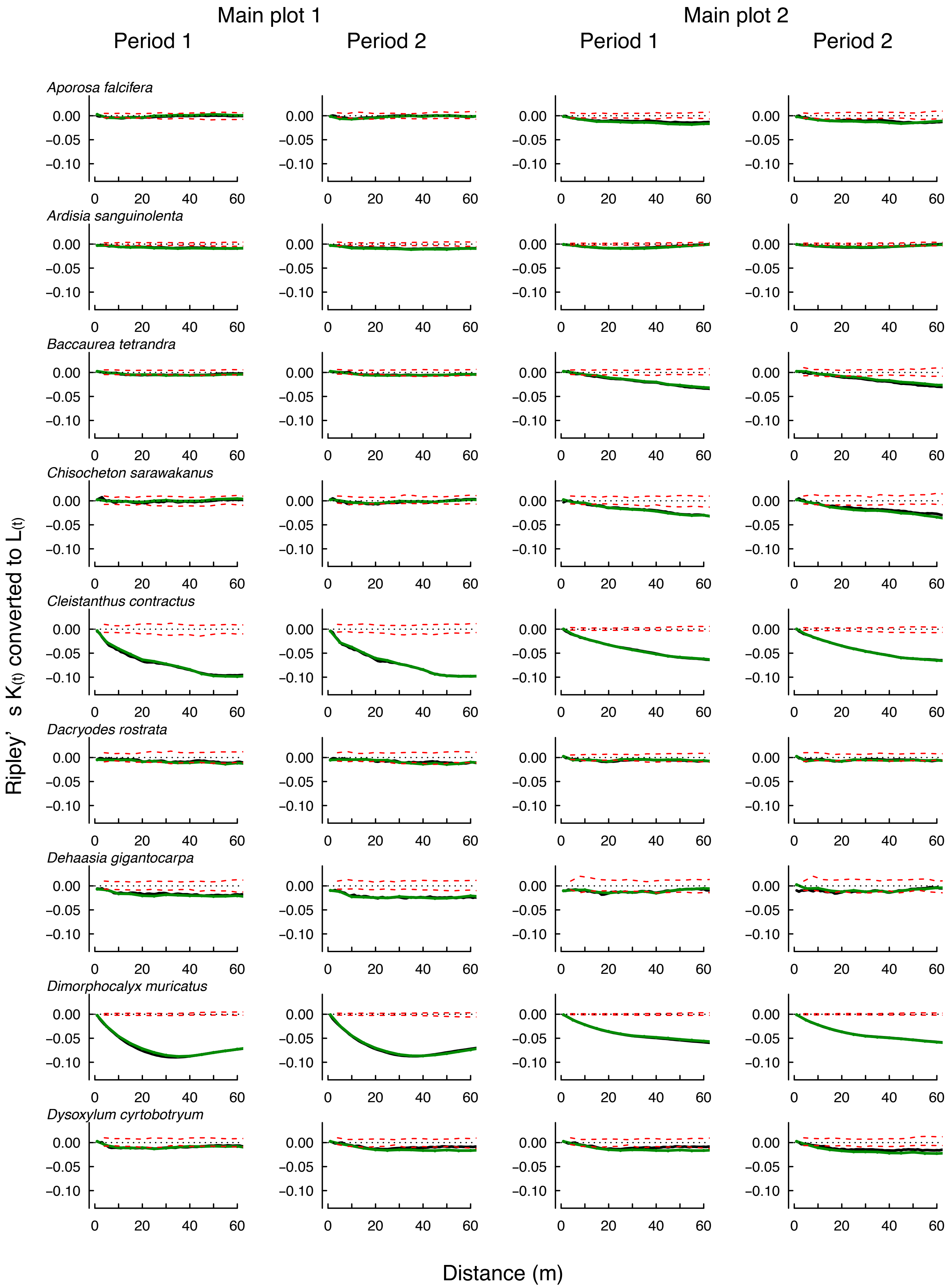

Main plot 1
Main plot 2
Period 1
Period 2
Period 1
Period 2
Aporosa falcifera
Ardisia sanguinolenta
Baccaurea tetrandra
Chisocheton sarawakanus
Cleistanthus contractus
Dacryodes rostrata
Dehaasia gigantocarpa
Dimorphocalyx muricatus
Dysoxylum cyrtobotryum
0.00
−0.05
−0.10
0
20
40
60
Ripley's K(t) converted to L(t)
Distance (m)

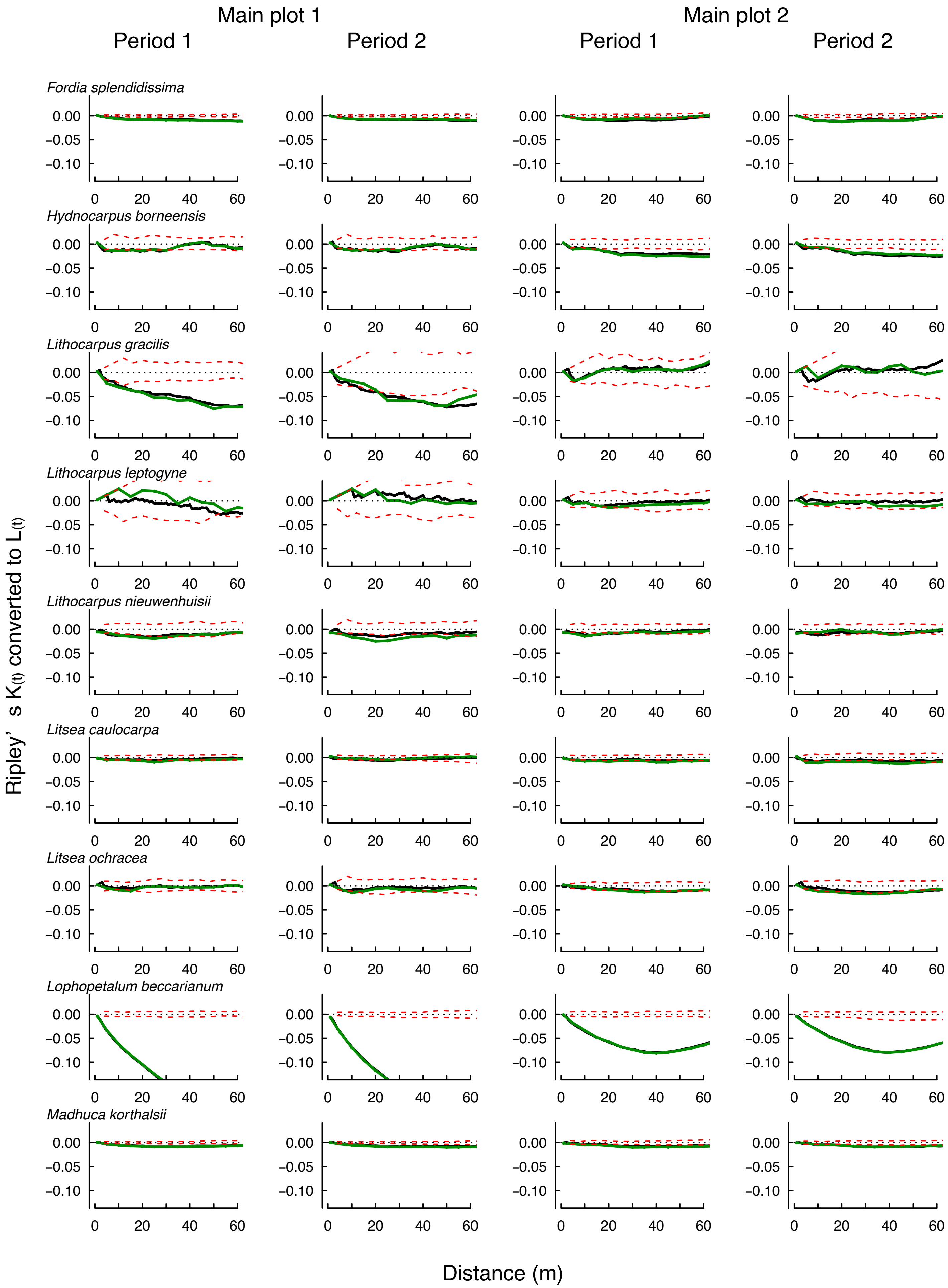
Main plot 1
Main plot 2
Period 1
Period 2
Period 1
Period 2
Fordia splendidissima
Hydnocarpus borneensis
Lithocarpus gracilis
Lithocarpus leptogyne
Lithocarpus nieuwenhuisii
Litsea caulocarpa
Litsea ochracea
Lophopetalum beccarianum
Madhuca korthalsii
0.00
−0.05
−0.10
0
20
40
60
Ripley’ s K(t) converted to L(t)
Distance (m)

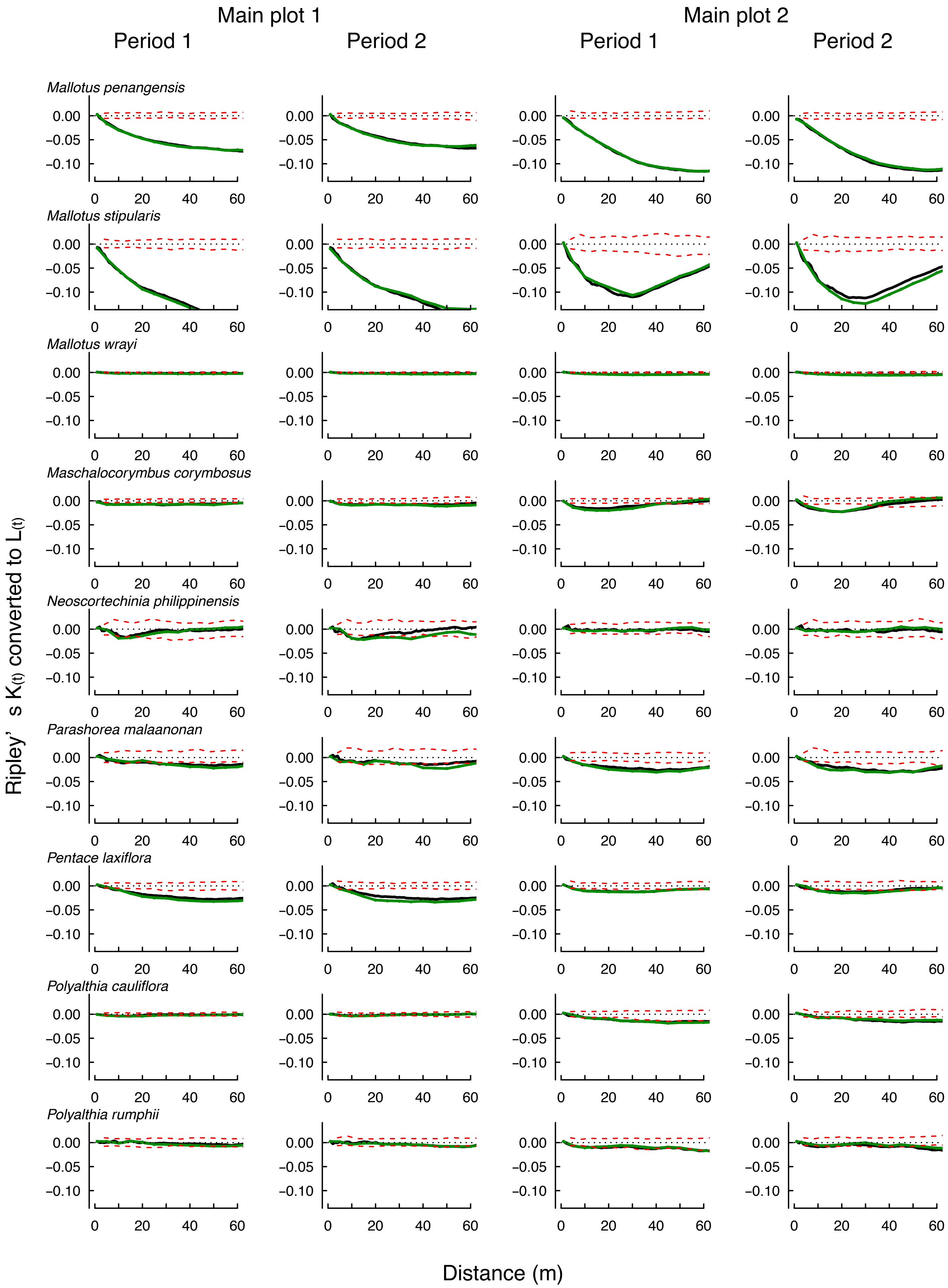

Main plot 1
Main plot 2
Period 1
Period 2
Period 1
Period 2
Mallotus penangensis
Mallotus stipularis
Mallotus wrayi
Maschalocorymbus corymbosus
Neoscortechinia philippinensis
Parashorea malaanonan
Pentace laxiflora
Polyalthia cauliflora
Polyalthia rumphii
0.00
−0.05
−0.10
0
20
40
60
Ripley's K(t) converted to L(t)
Distance (m)

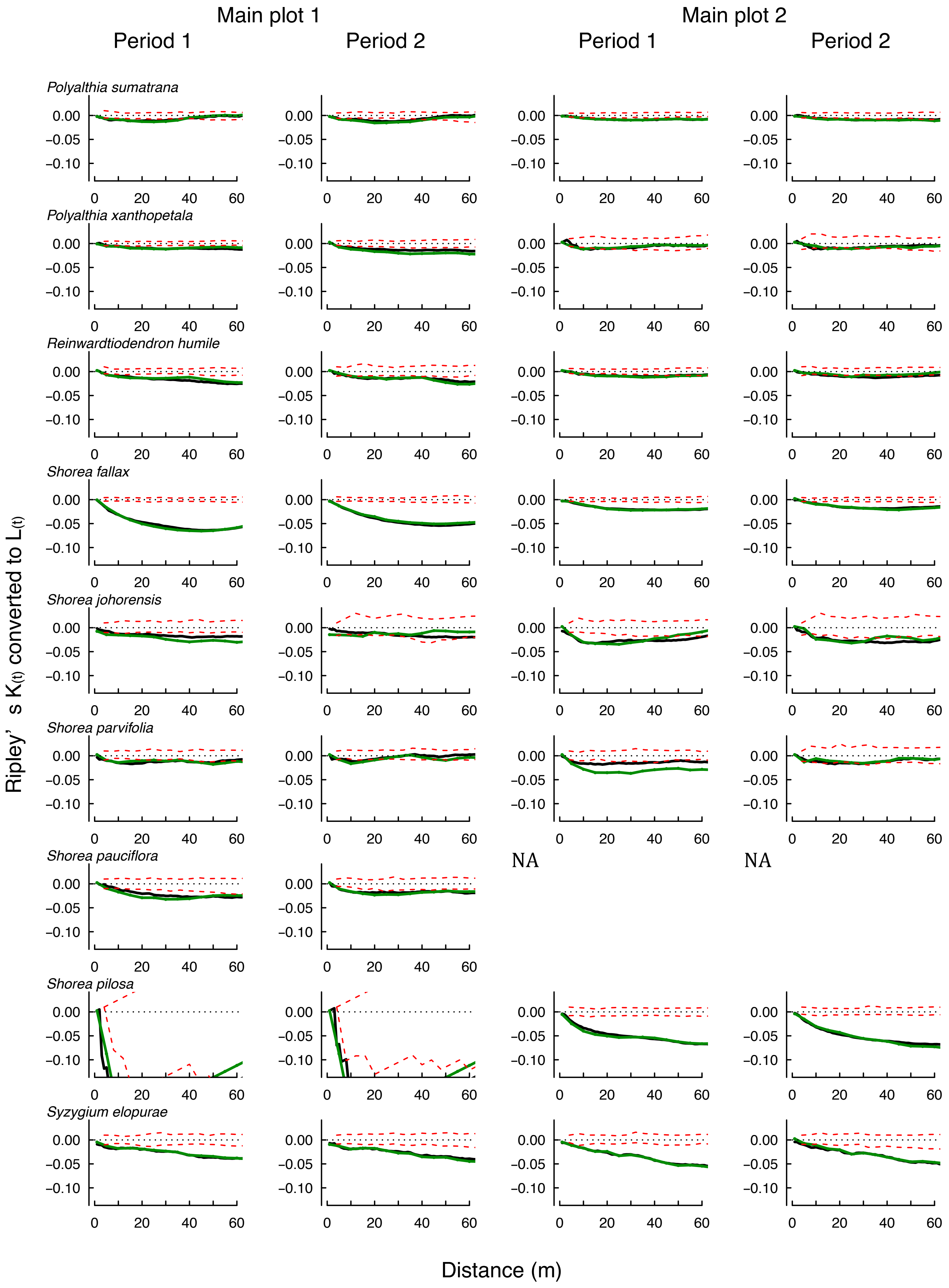

Main plot 1
Main plot 2
Period 1
Period 2
Period 1
Period 2
Polyalthia sumatrana
Polyalthia xanthopetala
Reinwardtiodendron humile
Shorea fallax
Shorea johorensis
Shorea parvifolia
Shorea pauciflora
Shorea pilosa
Syzygium elopurae
NA
NA
0.00
−0.05
−0.10
0
20
40
60
Ripley' s K(t) converted to L(t)
Distance (m)

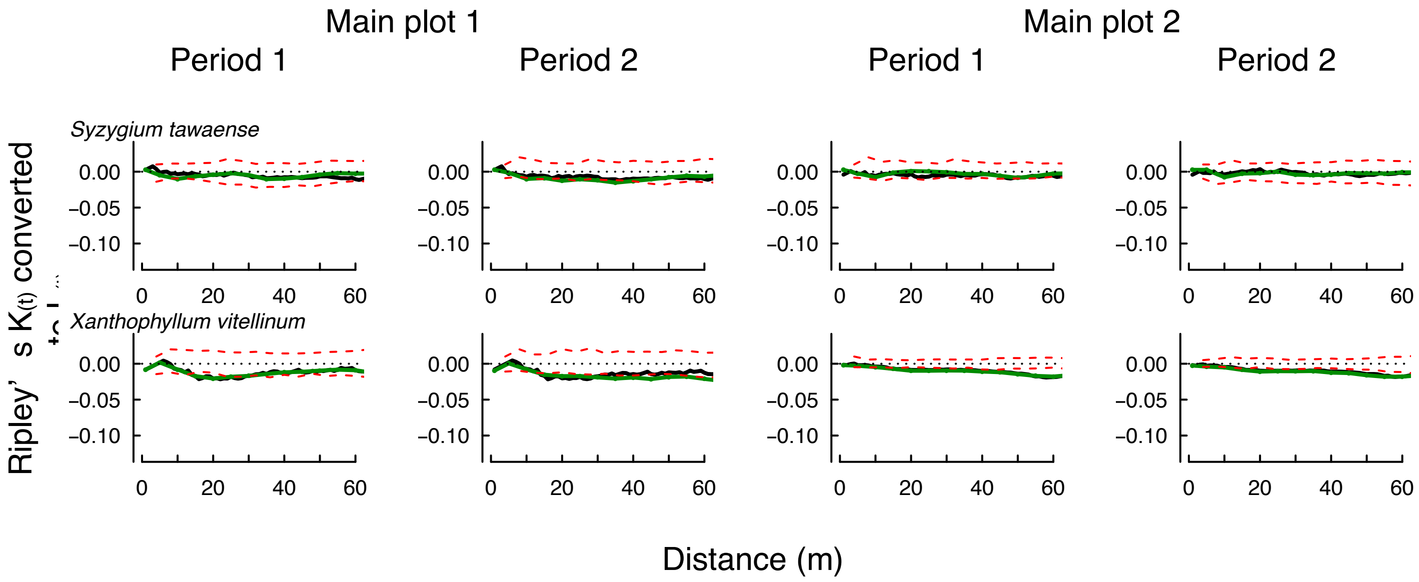

Main plot 1
Main plot 2
Period 1
Period 2
Period 1
Period 2
Syzygium tawaense
Xanthophyllum vitellinum
Ripley's K(t) converted
0.00
−0.05
−0.10
0
20
40
60
Distance (m)

Appendix S7: Randomization runs.

[Newbery/Stoll "Including tree spatial extension in the evaluation of neighborhood competition effects in Bornean rain forest"]

Appendix S7: Figure S1. Single species panel-graphs (CON)

Outcome of the 48 species' analyses using 100 randomizations of tree positions (method of Newbery & Stoll, 2013), for the spatial model 'larsm/reloc/crown' [linear decay], shown in alphabetical order. (The excluded 10 species have their names in grey.) Averages of the runs are shown as means ± 3 SE's, in green if the zero-value for the variables growth (P_1, P_2 and change P_2-P_1; CON effect size) and survival (P_1 and P_2; raw CON estimate, β) was within the implied confidence limits, otherwise in red to indicate a significant difference. The estimates from the empirical data are shown as black circles, open if they lay within the limits and closed if outside of them.

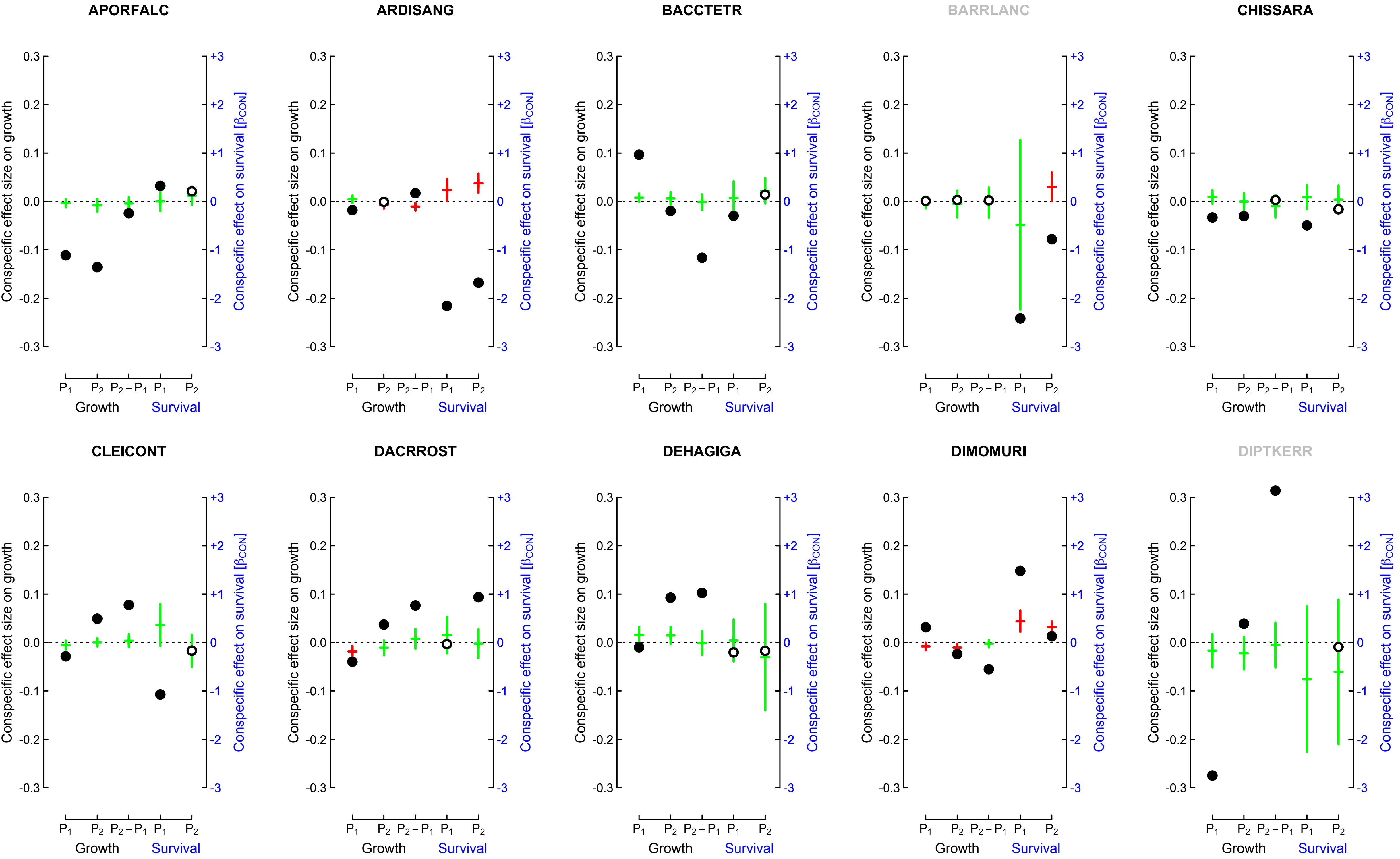
APORFALC
ARDISANG
BACCTETR
BARRLANC
CHISSARA
CLEICONT
DACRROST
DEHAGIGA
DIMOMURI
DIPTKERR
Conspecific effect size on growth
Conspecific effect on survival [βCON]
0.3
0.2
0.1
0.0
-0.1
-0.2
-0.3
+3
+2
+1
0
-1
-2
-3
P1
P2
P2 – P1
P1
P2
Growth
Survival

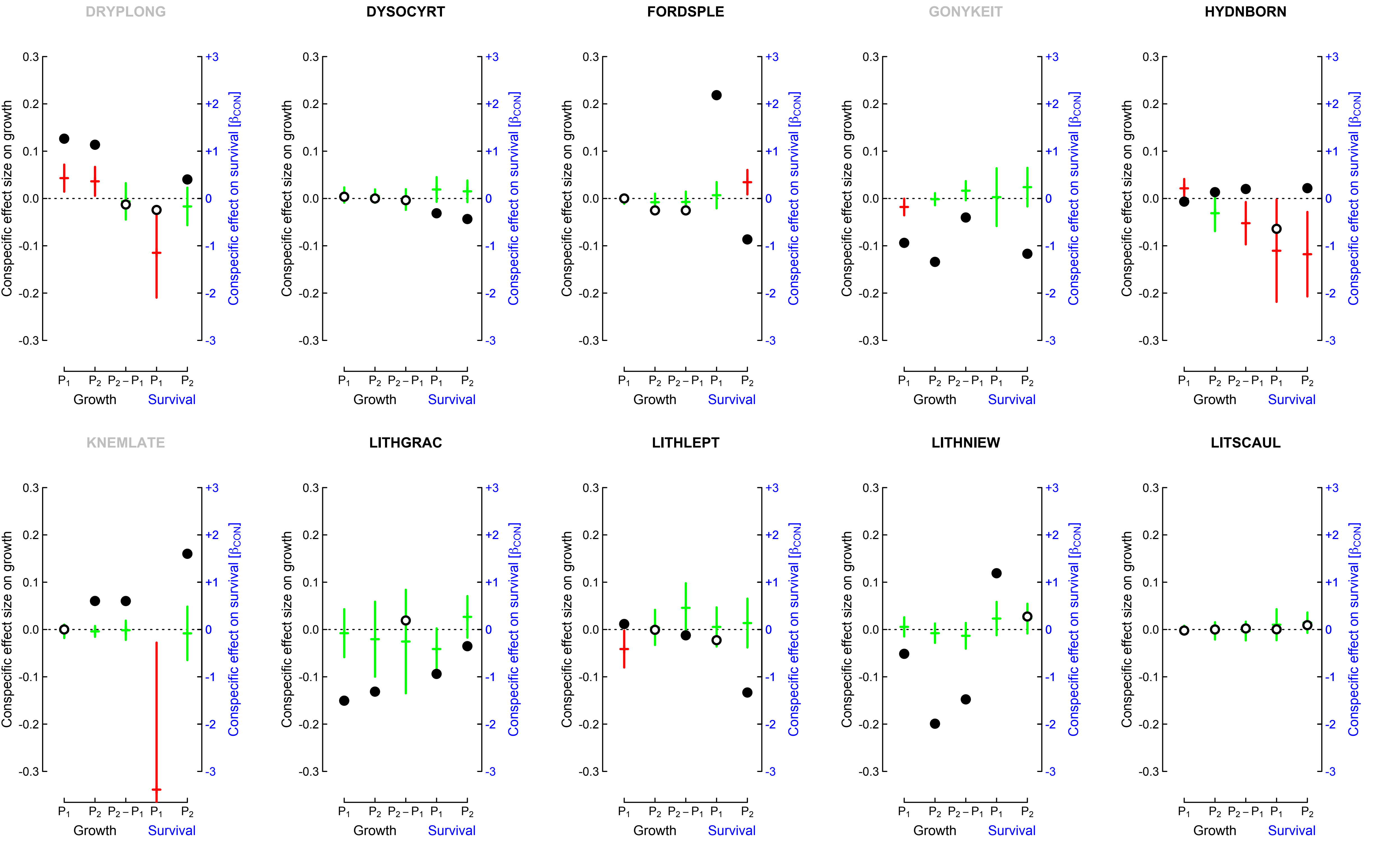

DRYPLONG
DYSOCYRT
FORDSPLE
GONYKEIT
HYDNBORN
KNEMLATE
LITHGRAC
LITHLEPT
LITHNIEW
LITSCAUL
Conspecific effect size on growth
Conspecific effect on survival [βCON]
P1
P2
P2 – P1
Growth
Survival

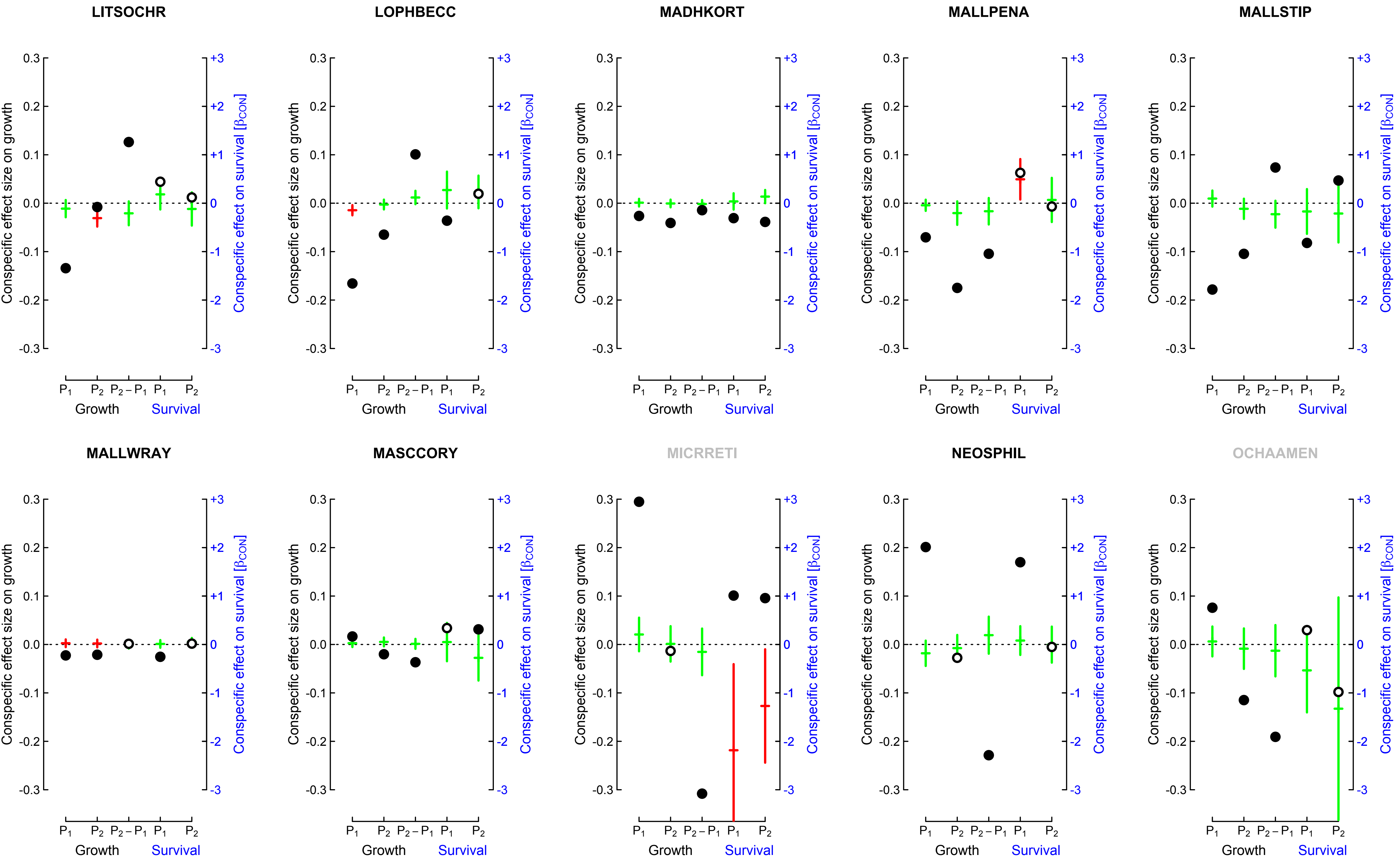
LITSOCHR
LOPHBECC
MADHKORT
MALLPENA
MALLSTIP
MALLWRAY
MASCCORY
MICRRETI
NEOSPHIL
OCHAAMEN
Conspecific effect size on growth
Conspecific effect on survival [βCON]
0.3
0.2
0.1
0.0
-0.1
-0.2
-0.3
+3
+2
+1
0
-1
-2
-3
P1
P2
P2 – P1
P1
P2
Growth
Survival

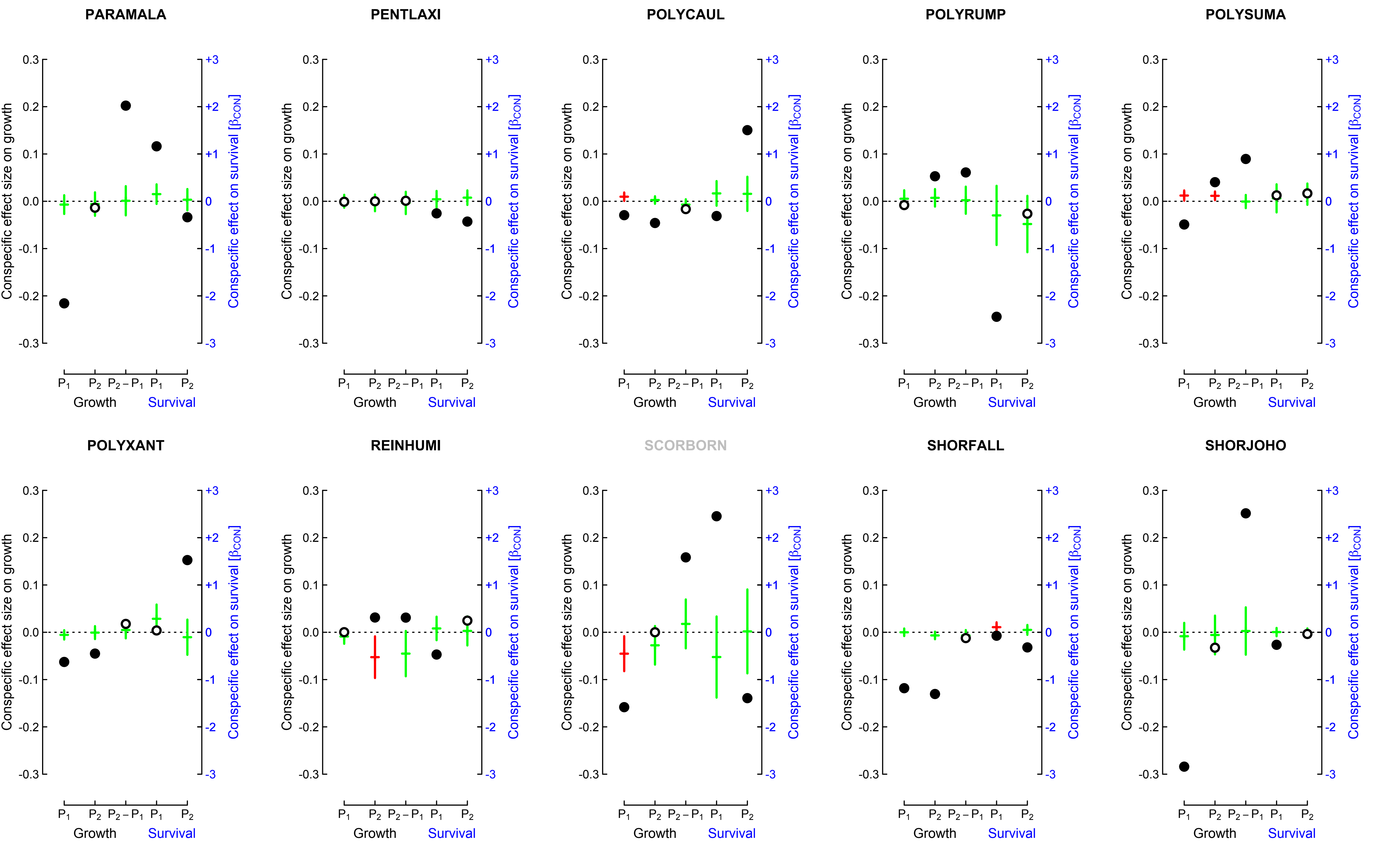
PARAMALA
PENTLAXI
POLYCAUL
POLYRUMP
POLYSUMA
POLYXANT
REINHUMI
SCORBORN
SHORFALL
SHORJOHO
Conspecific effect size on growth
Conspecific effect on survival [βCON]
P1
P2
P2 – P1
Growth
Survival

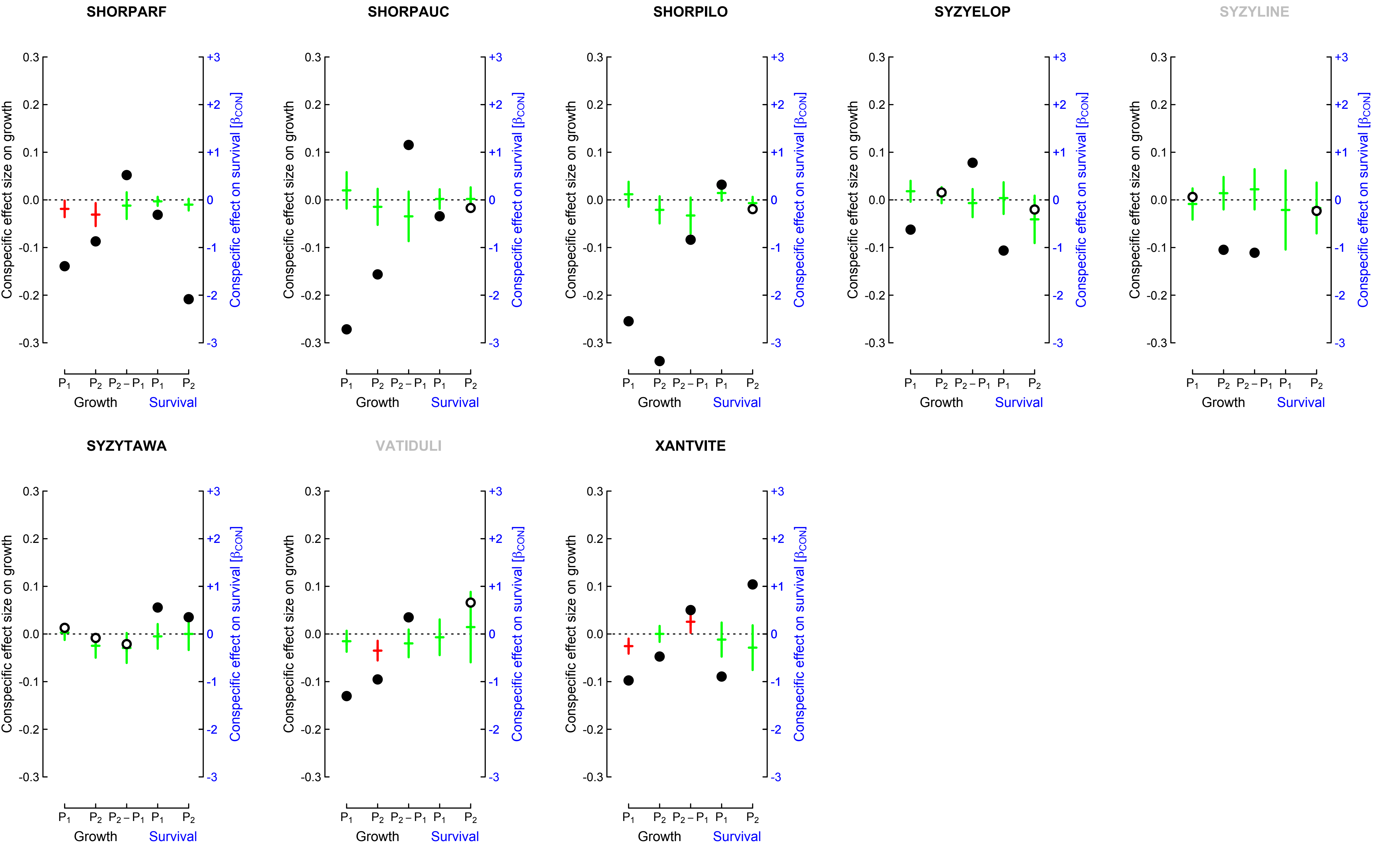
SHORPARF
SHORPAUC
SHORPILO
SYZYELOP
SYZYLINE
SYZYTAWA
VATIDULI
XANTVITE
Conspecific effect size on growth
Conspecific effect on survival [βCON]
0.3
0.2
0.1
0.0
-0.1
-0.2
-0.3
+3
+2
+1
0
-1
-2
-3
P1
P2
P2 – P1
P1
P2
Growth
Survival

Appendix S7: Figure S2. Single species panel-graphs (HET)

Outcome of the 48 species' analyses using 100 randomizations of tree positions (method of Newbery & Stoll, 2013), for the spatial model 'larsm/reloc/crown' [linear decay], shown in alphabetical order. (The excluded 10 species have their names in grey.) Averages of the runs are shown as means ± 3 SE's, in green if the zero-value for the variables growth (P_1, P_2 and change P_2-P_1; HET effect size) and survival (P_1 and P_2; raw HET estimate, β) was within the implied confidence limits, otherwise in red to indicate a significant difference. The estimates from the empirical data are shown as black circles, open if they lay within the limits and closed if outside of them.

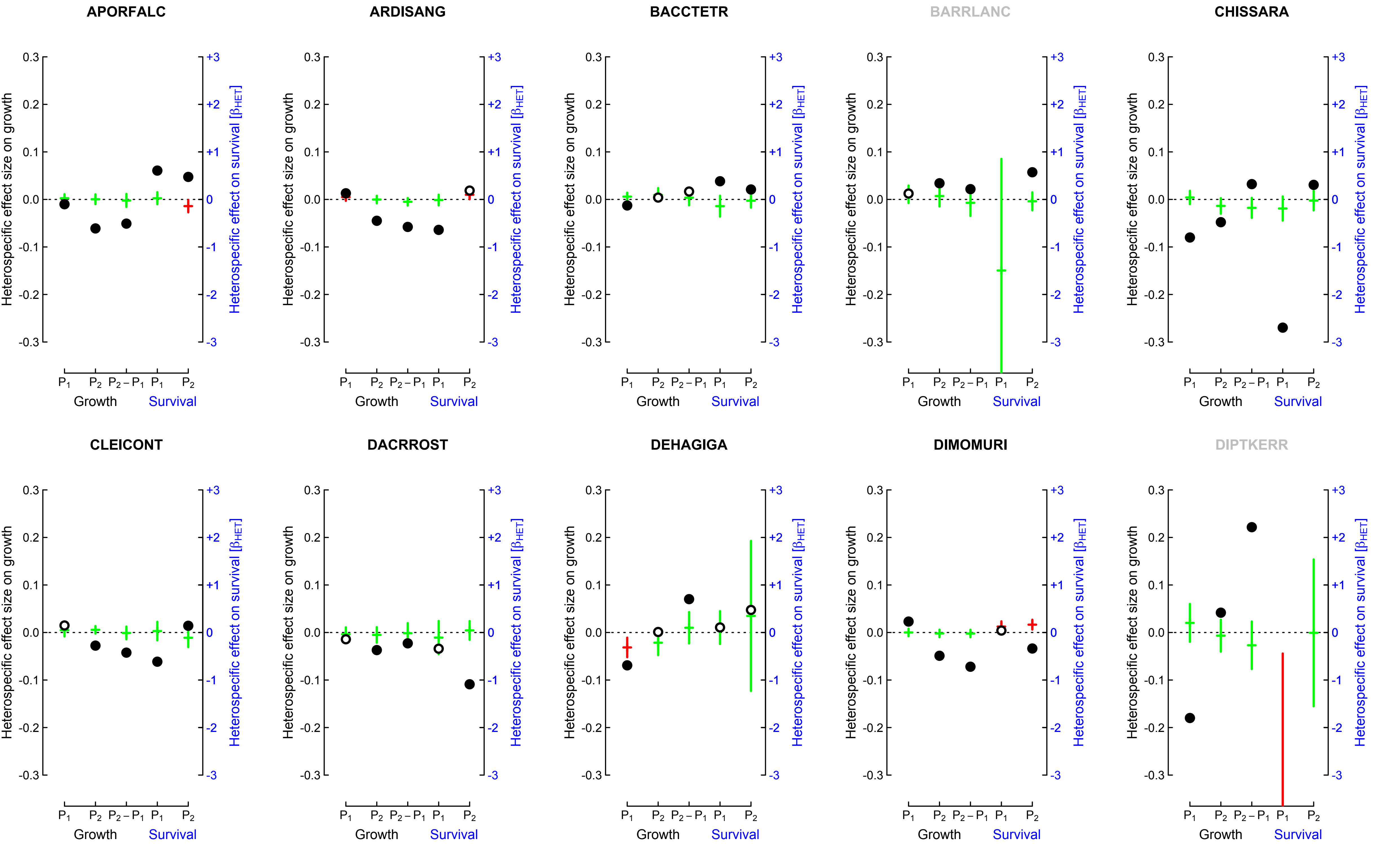
APORFALC
ARDISANG
BACCTETR
BARRLANC
CHISSARA
CLEICONT
DACRROST
DEHAGIGA
DIMOMURI
DIPTKERR
Heterospecific effect size on growth
Heterospecific effect on survival [βHET]
0.3
0.2
0.1
0.0
-0.1
-0.2
-0.3
+3
+2
+1
0
-1
-2
-3
P1
P2
P2 − P1
P1
P2
Growth
Survival

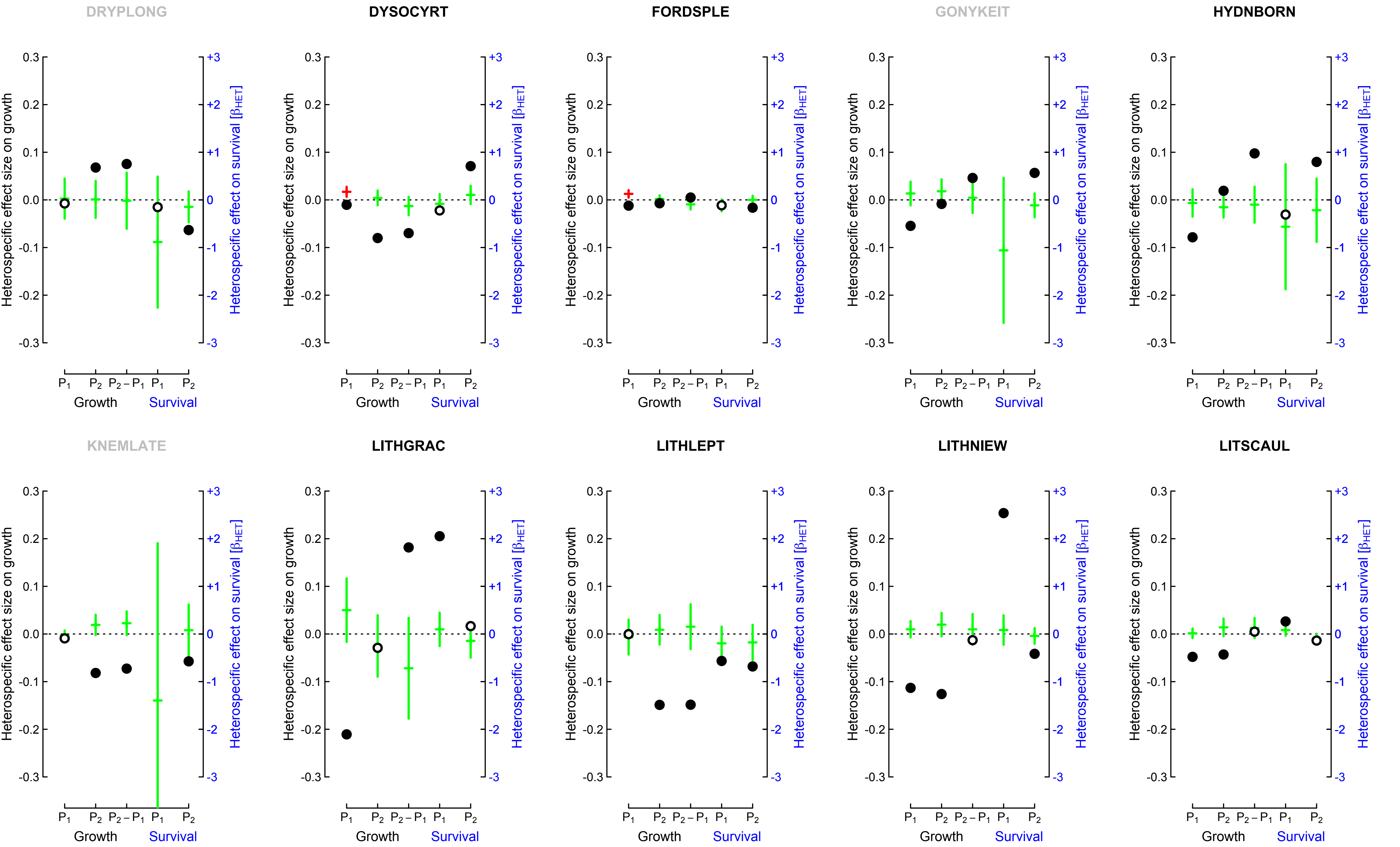
DRYPLONG
DYSOCYRT
FORDSPLE
GONYKEIT
HYDNBORN
KNEMLATE
LITHGRAC
LITHLEPT
LITHNIEW
LITSCAUL
Heterospecific effect size on growth
Heterospecific effect on survival [β_{HET}]
P_1 P_2 $P_2 - P_1$ P_1 P_2
Growth
Survival

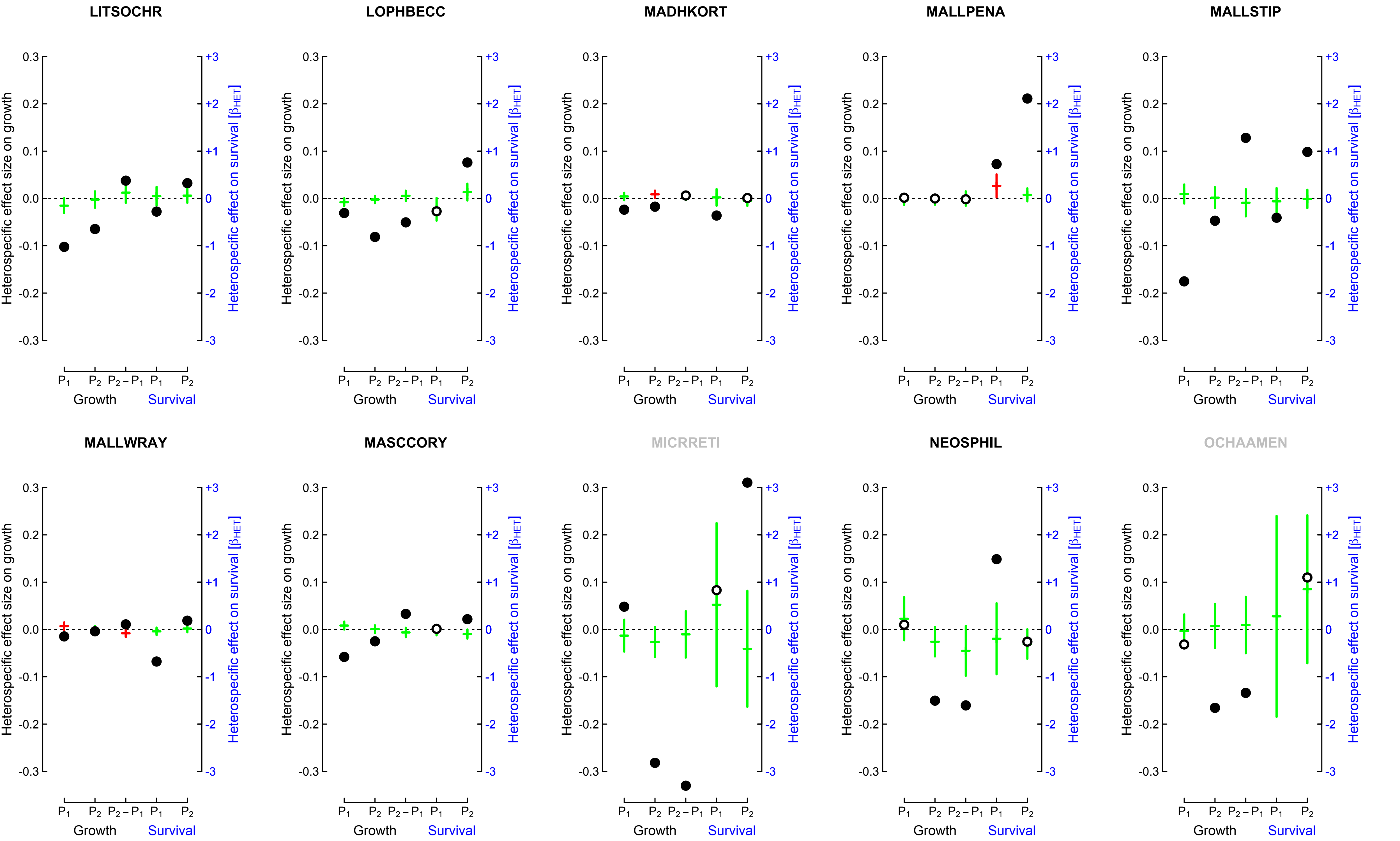
LITSOCHR
LOPHBECC
MADHKORT
MALLPENA
MALLSTIP
MALLWRAY
MASCCORY
MICRRETI
NEOSPHIL
OCHAAMEN
Heterospecific effect size on growth
Heterospecific effect on survival [β_{HET}]
0.3
0.2
0.1
0.0
-0.1
-0.2
-0.3
+3
+2
+1
0
-1
-2
-3
P_1
P_2
$P_2 - P_1$
P_1
P_2
Growth
Survival

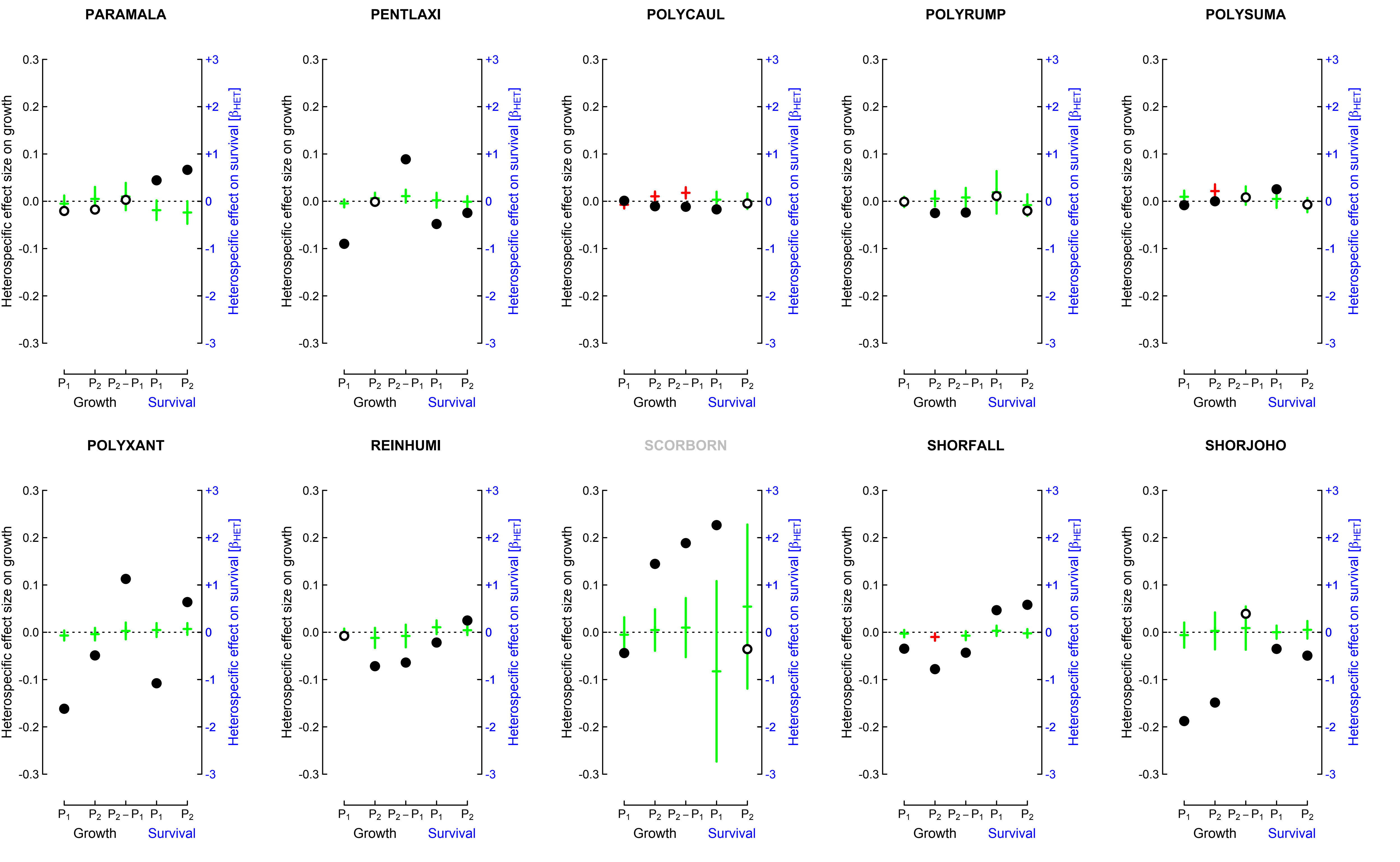
PARAMALA
PENTLAXI
POLYCAUL
POLYRUMP
POLYSUMA
POLYXANT
REINHUMI
SCORBORN
SHORFALL
SHORJOHO
Heterospecific effect size on growth
Heterospecific effect on survival [β_{HET}]
0.3
0.2
0.1
0.0
-0.1
-0.2
-0.3
+3
+2
+1
0
-1
-2
-3
P_1
P_2
$P_2 - P_1$
P_1
P_2
Growth
Survival

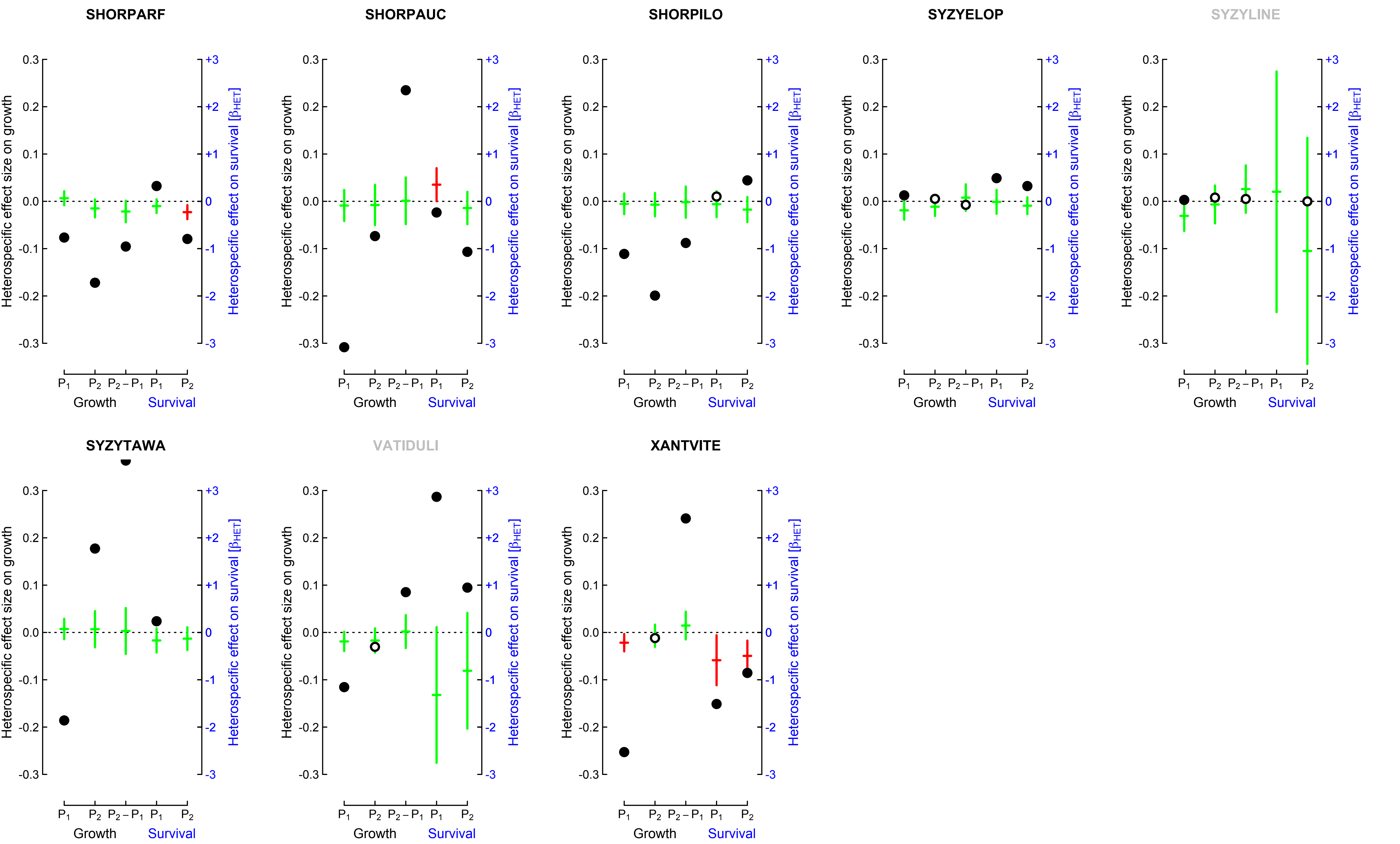
SHORPARF
SHORPAUC
SHORPILO
SYZYELOP
SYZYLINE
SYZYTAWA
VATIDULI
XANTVITE
Heterospecific effect size on growth
Heterospecific effect on survival [β_{HET}]
0.3
0.2
0.1
0.0
-0.1
-0.2
-0.3
+3
+2
+1
0
-1
-2
-3
P_1
P_2
$P_2 - P_1$
P_1
P_2
Growth
Survival

Appendix S7: Figure S3. The change in effect size on growth between P_1 and P_2 versus effect size on survival in P_1, as fitted regression lines from the 100 randomizations of tree positions (method of Newbery & Stoll, 2013), for the spatial model 'larsm/reloc/crown' [lin decay]: (a) CON effects based using all 38 selected species, (b) CON effects limited to the 24 species where P was < 0.1 for survival effects in $P_{1;}$ and (c) and (d) correspondingly the same for HET effects. Lines with positive and negative slopes are shown in green and blue respectively: continuous lines are significant at $P \geq 0.05$, dashed lines are not significant. The line from the regression using the empirical data is in red (continuous), as also shown for (a) on the last line of Table 5a and on Fig. 2b), for (b) on the last line of Table 5b, and for (c) on Fig. 3b.

(a) CON, 38 spp.

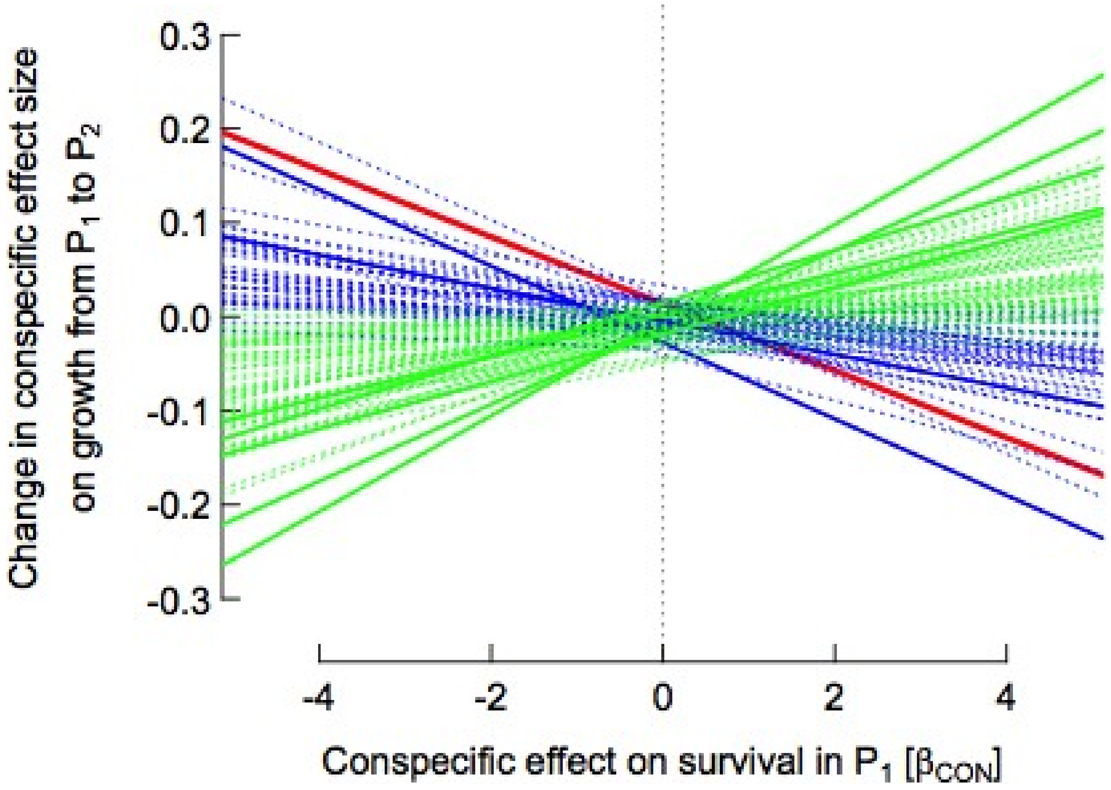

0.3
0.2
0.1
0.0
-0.1
-0.2
-0.3
Change in conspecific effect size on growth from P1 to P2
-4
-2
0
2
4
Conspecific effect on survival in P1 [βCON]

(b) CON, 24 spp.

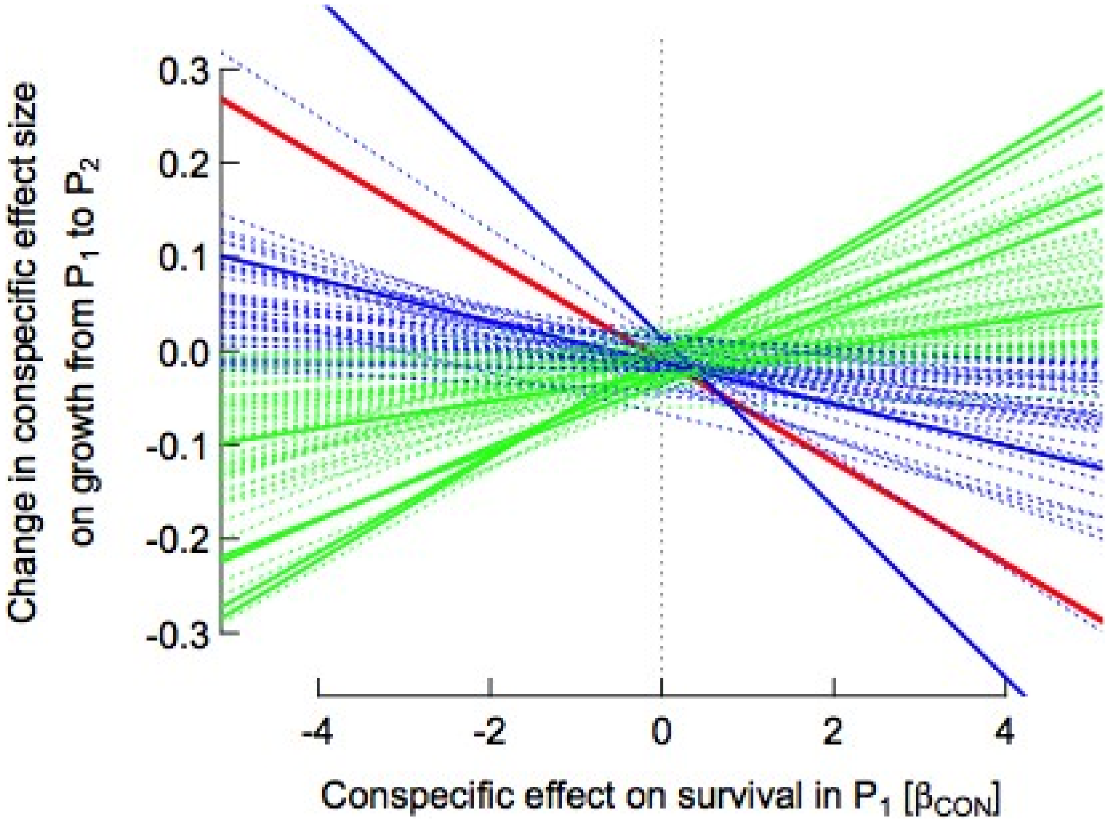

Change in conspecific effect size on growth from P1 to P2
0.3
0.2
0.1
0.0
-0.1
-0.2
-0.3
-4
-2
0
2
4
Conspecific effect on survival in P1 [βCON]

(c) HET, 38 spp.

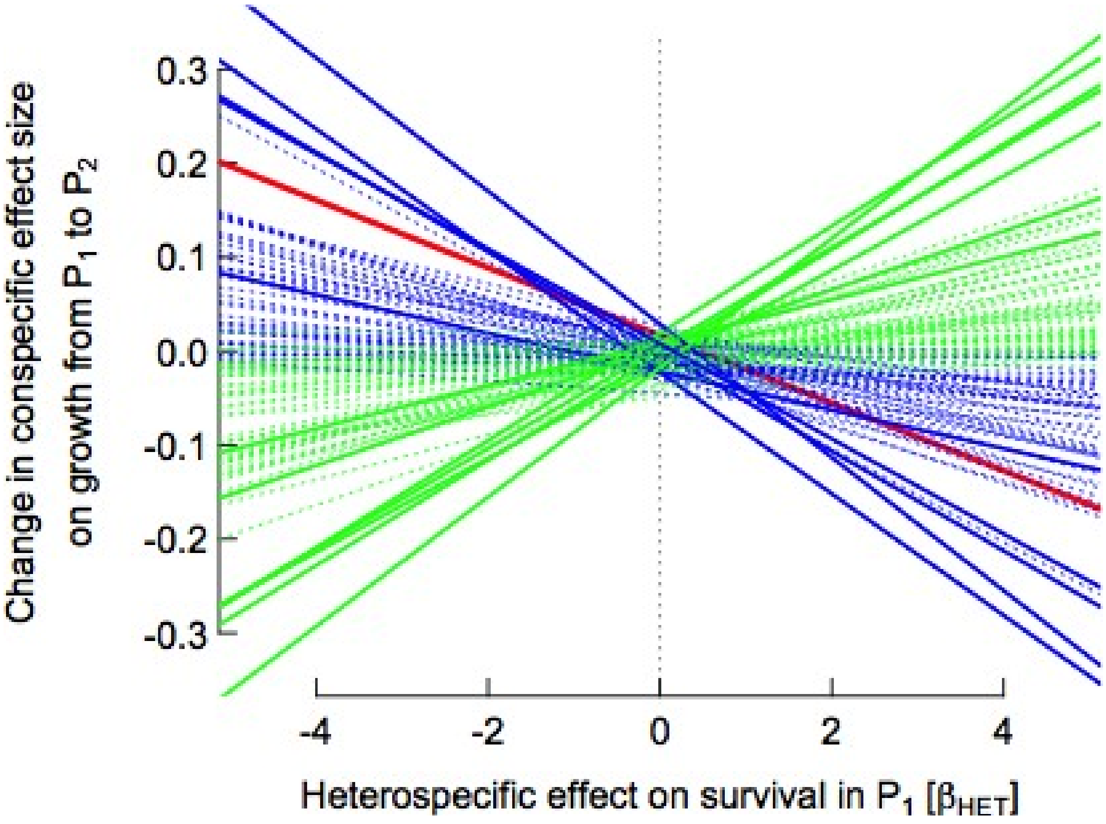
0.3
0.2
0.1
0.0
-0.1
-0.2
-0.3
Change in conspecific effect size on growth from P_1 to P_2
-4
-2
0
2
4
Heterospecific effect on survival in P_1 [β_{HET}]

(c) **HET, 24 spp.**

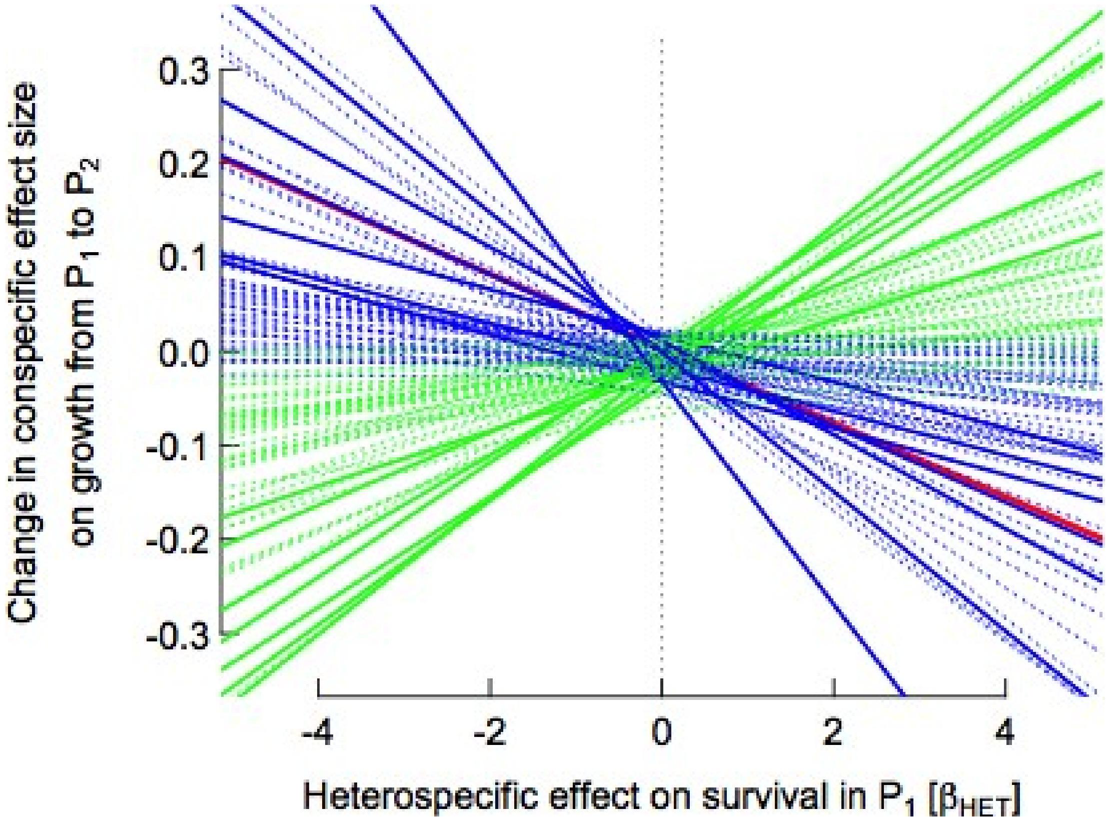

Change in conspecific effect size on growth from P1 to P2
0.3
0.2
0.1
0.0
-0.1
-0.2
-0.3
-4
-2
0
2
4
Heterospecific effect on survival in P1 [βHET]
